# Gold-Aluminyl and Gold-Diarylboryl Complexes: Bonding and Reactivity with Carbon Dioxide

**DOI:** 10.1021/acs.inorgchem.2c00174

**Published:** 2022-05-05

**Authors:** Diego Sorbelli, Elisa Rossi, Remco W.A. Havenith, Johannes E.M.N. Klein, Leonardo Belpassi, Paola Belanzoni

**Affiliations:** † Department of Chemistry, Biology and Biotechnology, University of Perugia, Via Elce di Sotto, 8, 06123 Perugia, Italy; ‡ CNR Institute of Chemical Science and Technologies “Giulio Natta” (CNR-SCITEC), Via Elce di Sotto, 8, 06123 Perugia, Italy; § Chemistry of (bio)Molecular Materials and Devices, Stratingh Institute for Chemistry, Faculty of Science and Engineering and Zernike Institute for Advanced Materials, University of Groningen, Nijenborgh 4, 9747 AG Groningen, The Netherlands; 4 Molecular Inorganic Chemistry, Stratingh Institute for Chemistry, Faculty of Science and Engineering, University of Groningen, Nijenborgh 4, 9747 AG Groningen, The Netherlands; ∥ Zernike Institute for Advanced Materials, University of Groningen, Nijenborgh 4, 9747 AG Groningen, The Netherlands; ⊥ Ghent Quantum Chemistry Group, Department of Chemistry, Ghent University, Krijgslaan 281 (S3), B-9000 Gent, Belgium

## Abstract

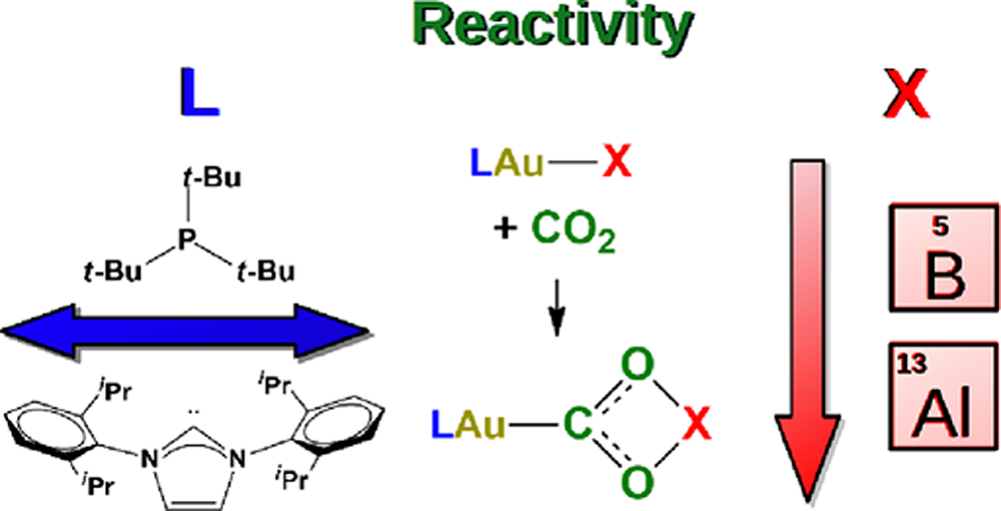

The unconventional carbon dioxide insertion reaction of a gold-aluminyl [^t^Bu_3_PAuAl(NON)] complex has been recently shown to be related to the electron-sharing character of the Au–Al bond that acts as a nucleophile and stabilizes the insertion product through a radical-like behavior. Since a gold-diarylboryl [IPrAuB(*o*-tol)_2_] complex with similar reactivity features has been recently reported, in this work we computationally investigate the reaction of carbon dioxide with [LAuX] (L = phosphine, N-heterocyclic carbene (NHC); X = Al(NON), B(*o*-tol)_2_) complexes to get insights into the Al/B anionic and gold ancillary ligand effects on the Au–Al/B bond nature, electronic structure, and reactivity of these compounds. We demonstrate that the Au–Al and Au–B bonds possess a similar electron-sharing nature, with diarylboryl complexes displaying a slightly more polarized bond as Au(δ^+^)–B(δ^–^). This feature reduces the radical-like reactivity toward CO_2_, and the Al/B anionic ligand effect is found to favor aluminyls over boryls, despite the greater oxophilicity of B. Remarkably, the ancillary ligand of gold has a negligible electronic trans effect on the Au–X bond and only a minor impact on the formation of the insertion product, which is slightly more stable with carbene ligands. Surprisingly, we find that the modification of the steric hindrance at the carbene site may exert a sizable control over the reaction, with more sterically hindered ligands thermodynamically disfavoring the formation of the CO_2_ insertion product.

## Abstract 2

The Au−Al and Au−B bonds have both an electron-sharing nature, with the diarylboryl gold complexes displaying a more polarized Au^δ+^−B^δ−^ bond. The gold ligand (phosphine or N-heterocyclic carbene) has a negligible electronic effect on the Au−X bond, consistently with a radical-like reactivity of the complexes with carbon dioxide, which favors the gold-aluminyl over the gold-diarylboryl complexes.

## Introduction

**Scheme 1:**
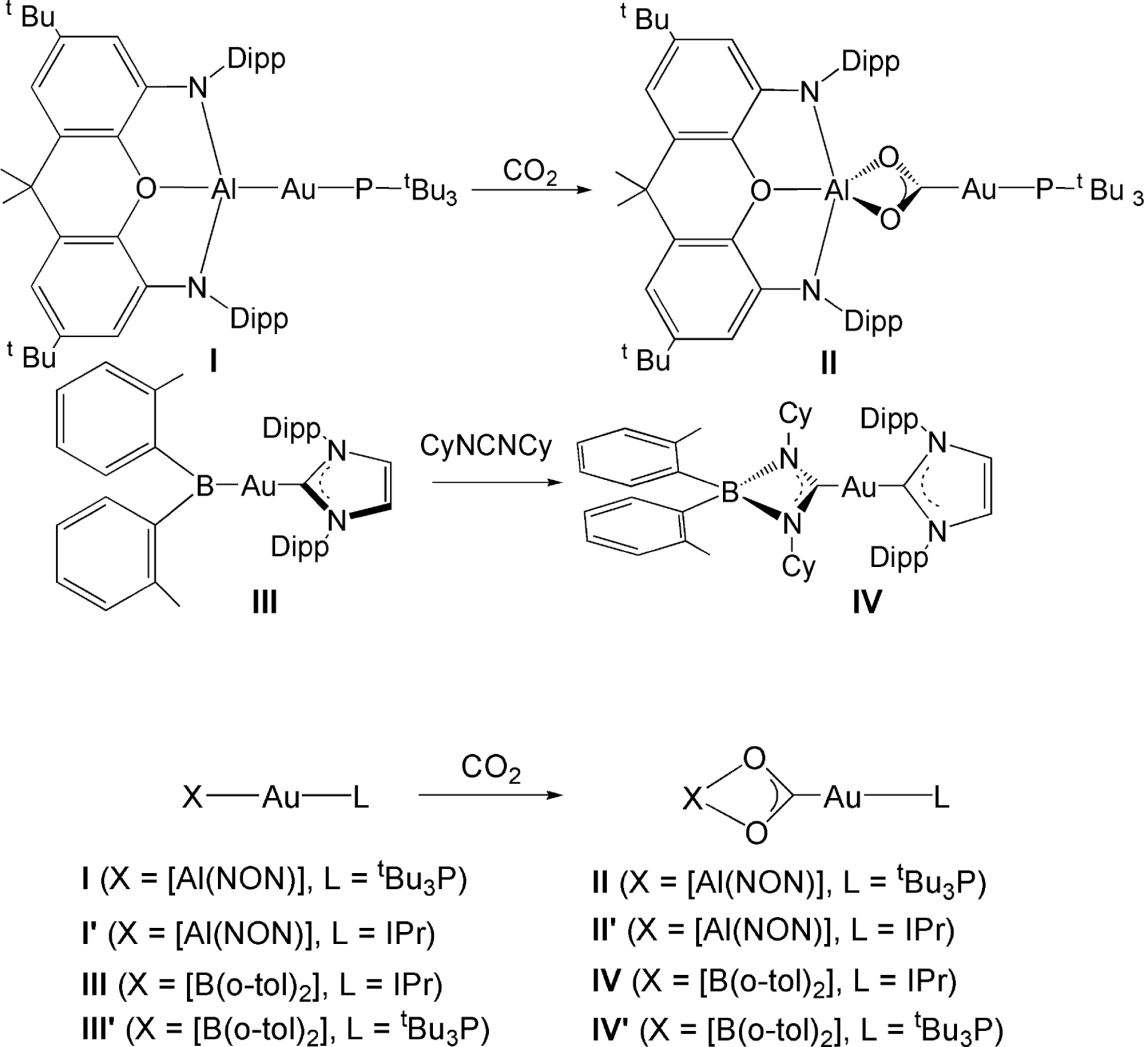
Examples of “Nucleophilic” Gold (**I** and **III**) Complexes and Their Characteristic Insertion Products (**II** and **IV**) CO_2_ insertion reaction into the Au–Al bond in the experimental (**I**) and model (**I′**) aluminyl-gold compounds and into the Au–B bond in the experimental (**III**) and model (**III′**) diarylboryl-gold compounds and their corresponding reaction products (**II**, **II′**, **IV**, and **IV′**, respectively).

Insertion of carbon dioxide into the Au–Al bond in the aluminyl [^t^Bu_3_PAuAl(NON)] (NON = 4,5-bis(2,6-diisopropylanilido)-2,7-di-*tert*-butyl-9,9-dimethylxanthene) complex **I**, leading to [^t^Bu_3_PAuCO_2_Al(NON)] product **II** (Scheme 1), where the CO_2_ carbon atom is coordinated to gold, was reported in 2019.^1^ This system has been recently investigated by some of us to shed light into the reaction mechanism and the key features of the Au–Al bond.^2^ A bimetallic reactivity has been shown, where the Au–Al bond behaves as the actual nucleophile, and the stability of the insertion product is strictly related to the stability of the [^t^Bu_3_AuCO_2_]· and [CO_2_Al(NON)]· radicals, consistently with an electron-sharing, weakly polarized Au–Al bond. The electrophilic behavior of Al also contributes to the interaction with CO_2_.

As a general result arising from our study, the reactivity of metal-aluminyl complexes with CO_2_ leading to the M-CO_2_ coordination mode cannot be considered as a probe for a highly polarized M(δ^–^)–Al(δ^+^) bond and for a nucleophilic behavior of the metal center. A strictly related diarylboryl gold complex, [IPrAuB(*o*-tol)_2_] (IPr = *N*,*N*′-bis(2,6-diisopropylphenyl)imidazole-2-ylidene) **III** (Scheme 1), has been more recently reported by Yamashita and co-workers to display a nucleophilic reactivity at the gold atom.^3^ The reaction of [IPrAuB(*o*-tol)_2_] with isocyanides and C=O- or C=N-containing compounds results in the formation of Au–C and B–O/N bonds (complex **IV**), which has suggested, analogously to the gold-aluminyl complex **I**, a nucleophilic behavior of the Au center. Mechanistic DFT studies on the diarylboryl gold complex [IPrAuB(*o*-tol)_2_] reaction with *N*,*N*-dimethylcarbodiimide CyNCNCy have been carried out.^3^ A three-step path has been proposed consisting of (i) an initial coordination of the C=N moiety to the B center to form a B···N=C=N intermediate followed by (ii) a migration of the gold center to attack the carbon atom of the carbodiimide functionality (this step has been considered as the revealing of a nucleophilic behavior of gold) and, finally, (iii) the formation of a B-containing four-membered ring (**IV** in Scheme 1). Notably, gold-boryl complex **III** involving aryl substituents is expected to differ from typical dioxy- and diamino-boryls such as Bpin (pin = pinacolate: 2,3-dimethyl-2,3 butanediolate), Bcat (cat = 1,2-O_2_C_6_H_4_), Bneop (neop = (OCH_2_)_2_CMe_2_), Bdan (dan = 1,8-diaminonaphthalene), etc., mainly in the role played by boron’s “empty” p orbitals and to exhibit stronger Lewis acidity at the boron center.^4^ Although experimental evidence for the reaction of complex **III** with carbon dioxide has not been reported, the reduction of CO_2_ to CO catalyzed by a copper boryl complex [IPrCu(Bpin)] has been observed to occur in solution under mild conditions,^5^ and the reaction mechanism has been computationally studied.^6^ Very recently, some of us have computationally investigated the analogous reactivity with isostructural gold-aluminyl, gold-gallyl, and gold-indyl complexes, [^t^Bu_3_PAuX(^Si^NON)]^−^ (X = Al, Ga, and In, ^Si^NON = [O(SiMe_2_NDipp)_2_]^2–^, Dipp = 2,6-^i^Pr_2_C_6_H_3_), demonstrating that this is kinetically and thermodynamically favorable only for the gold-aluminyl complex.^7^ The highly electron-sharing nature of the Au–Al bond compared to the increasingly polar Au–Ga and Au–In bonds has been shown to single out the aluminyl ligand among Group 13 analogues. Given the unique behavior of the gold-aluminyl complexes and their peculiar features with respect to gold-gallyl and gold-indyl analogues, insertion of carbon dioxide into the Au–B bond in the strictly related gold-boryl complex **III** is definitely worth exploring to advance our knowledge on the nature of this new type of bond and on the supposed nucleophilicity of the gold center. The nature of the ancillary gold ligand (phosphine-type in complex **I** or carbene-type in complex **III**) is also expected to have an influence on both the metal-boryl/aluminyl bond features and reactivity. On this issue, we should mention that, recently, for the copper-aluminyl [IPrCuAlSiN^Dipp^] (SiN^Dipp^ = {CH_2_SiMe_2_NDipp}_2_) complex, where the metal bears a carbene-type ancillary ligand, the reaction with carbon dioxide allowed the isolation and characterization of an insertion product similar to **II**.^8^ Conversely, the same reactivity has been explored with the phosphine-copper [^t^Bu_3_PCuAl(NON)] complex and the isolation of a **II**-type insertion product was not possible due to its extremely fast evolution to a copper-carbonate complex (resulting from CO extrusion).^9^ These findings suggest that the gold ancillary ligand may have a role in the reactivity that, due to the unprecedented gold chemistry displayed by these heterobinuclear complexes, needs to be yet undisclosed.

In this work, we precisely investigate the mechanism of the CO_2_ insertion into the [IPrAuB(*o*-tol)_2_] complex and the actual nucleophilic ability of Au within the interpretative framework provided in ref (2). To directly compare the aluminyl [Al(NON)]^−^ and boryl [B(*o*-tol)_2_]^−^ bonding properties toward Au and the reactivity of the corresponding complexes with carbon dioxide, a common [^t^Bu_3_PAu]^+^ metal fragment has been initially chosen (model complexes **III′** and **IV′**; Scheme 1). Successively, the experimental [IPrAu]^+^ metal fragment has been considered (complexes **III** and **IV**) and compared to the aluminyl model complexes **I′** and **II′** (Scheme 1) to get insight into the gold ancillary ligand effect.

Based on a comparative mechanistic and electron structure analysis, we show that gold-diarylboryl complexes feature a slightly more polarized covalent Au(δ^+^)–B(δ^–^) bond, which is responsible for a kinetically and thermodynamically less favored CO_2_ insertion for boryls than aluminyls. The main difference between the two Al/B anionic ligands lies in the reduced ability of the boryls to stabilize the insertion product, which is related to the reduced ability of the [B(*o*-tol)_2_]· radical to stabilize CO_2_. The gold ligand (phosphine or NHC) only slightly affects the reactivity, with the carbene-type ligand moderately favoring the insertion of CO_2_ into the Au–X bond for both the Al/B anionic ligands. The gold ligand effect is remarkably negligible on the electronic features of the covalent Au–X bond. However, preliminary results presented here suggest that, instead, the steric hindrance at the NHC site may have a sizable impact and may be used to control the CO_2_ insertion reaction.

## Results and Discussion

We start the study of complexes **I**, **III′**, **I′**, and **III** by quantitatively analyzing the nature of the Au–Al/Au–B bond since, precisely, the features of the Au–Al bond were shown to be key in determining the reactivity of **I** with CO_2_.^2^ The analysis is carried out following the same computational protocol already employed in our previous study.^2^ At first, we assess the best possible fragmentation of the complexes into the gold and boryl/aluminyl fragments, according to refs (10) and (11), which is based on a comparative energy decomposition analysis (EDA) approach.^12,13^ As discussed in the Supporting Information, the energy values reported in Tables S1–S4 clearly indicate that, in all the complexes, the doublet neutral [LAu]· and [X]· (L = ^t^Bu_3_P, IPr ; X = B(*o*-tol)_2_, Al(NON′)) fragments provide the best suitable fragmentation for the description of the Au–X bond. Then, we resort to the use of the charge displacement (CD) analysis^14−16^ in the framework of the natural orbitals for chemical valence^17,18^ scheme (CD-NOCV), coupled with the extended transition state NOCV (ETS-NOCV)^19^ approach, to quantitatively assess the features of the Au–X bond. In addition, we analyze the nature of the bonding interaction between [LAu]· and [X]· using the intrinsic bond orbital (IBO) analysis^20^ and the nucleophilic/electrophilic regions in the complexes by employing the dual descriptor for chemical reactivity.^21^

Subsequently, we combine mechanistic studies with the electronic structure analysis to explore the mechanism of the CO_2_ insertion into the Au–Al bond of **I′** and Au–B bond of **III′** and **III**. We note that the computational setup is exactly the same as in ref (2), that is, density functional theory (DFT) with the inclusion of relativistic effects, solvation (toluene), and dispersion corrections (see the Computational Details section) for a consistent comparison with the gold-aluminyl complex **I** results.

Results are presented and discussed so as to separately deal with the boryl and aluminyl anionic ligand effect and the gold ancillary ligand (namely, the *tert*-butyl phosphine (^t^Bu_3_P) and the N-heterocyclic carbene (IPr)) effect issues.

### Aluminyl vs Boryl – [^t^Bu_3_PAu]: Effect on the Au–X Bond

In this section, we show and discuss the Au–Al and Au–B bond analyses for complexes **I** and **III′**, which allows us to study in detail the aluminyl/boryl ligand effect for the same gold fragment (*i.e.*, [^t^Bu_3_PAu]).

**Figure 1:**
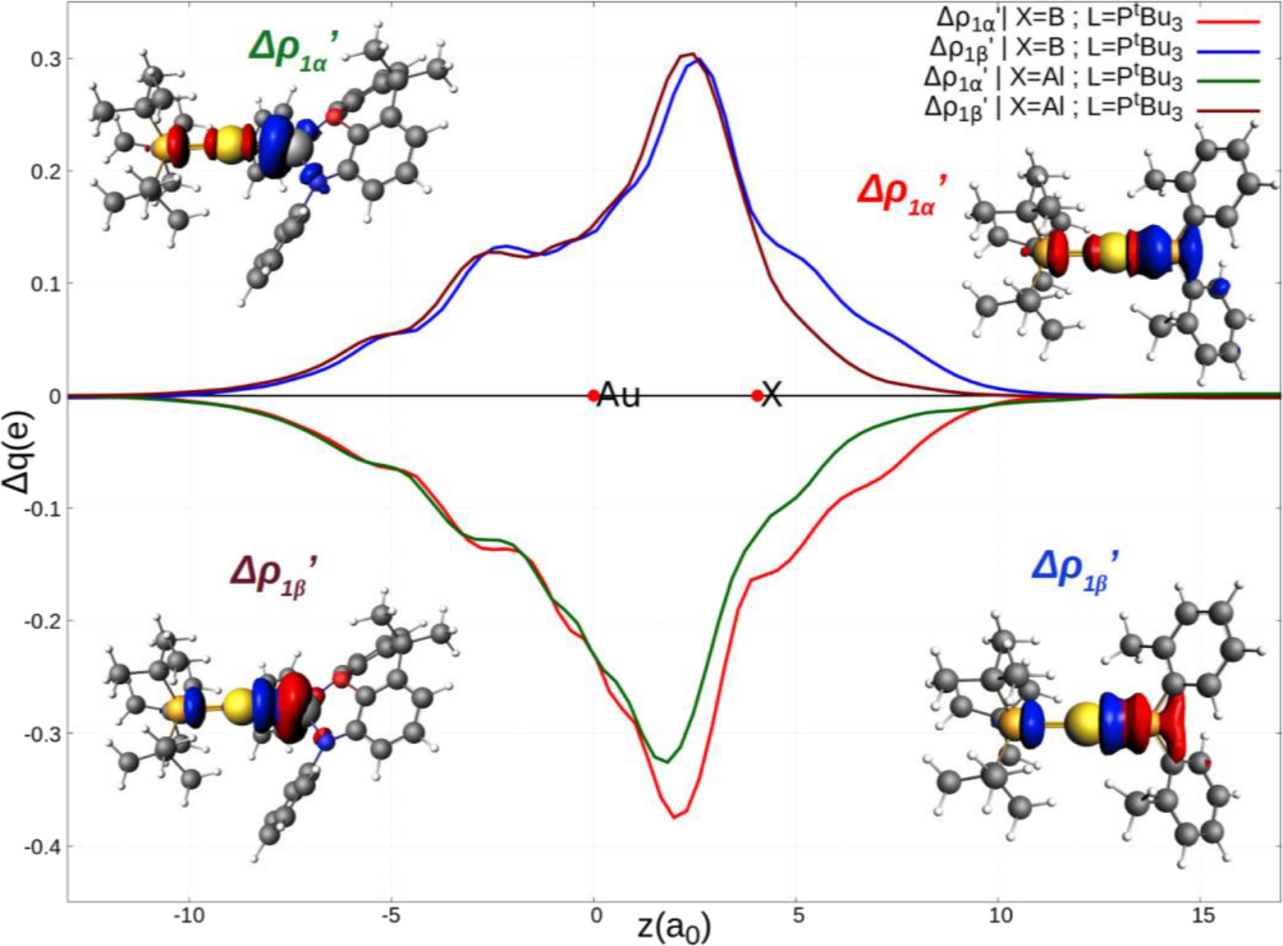
Charge displacement (CD-NOCV) curves associated with the Δρ_1α_′ and Δρ_1β_′ NOCV deformation densities for the interaction between doublet [^t^Bu_3_PAu]· and [X]· (X = Al(NON′), B(*o*-tol)_2_) fragments for complex **I** and **III′**, respectively. Red dots indicate the average position of the nuclei along the *z* axis. Positive (negative) values of the curve indicate right-to-left (left-to-right) charge transfer. Insets: isodensity surfaces of the Δρ_1α_′ and Δρ_1β_′ NOCV deformation densities for complex **I** (top left and bottom left, respectively) and for complex **III′** (top right and bottom right, respectively). The charge flux is red-to-blue. The isodensity value is 2 me/a_0_^3^ for all the surfaces. Results for **I** have been taken and adapted with permission from ref (2). Copyright 2021 American Chemical Society.

The main results of the CD-NOCV analysis for **I** and **III′** are summarized in Figure 1 and Table 1. The complete results can be found in Figures S1–S3 and Table S5 in the Supporting Information.

**Table 1:** Orbital Interaction Energies (Δ*E*_oi_^*k*^) (in kcal/mol) and Charge Transfer (CT^*k*^) (in Electrons, e) Associated with the First Two NOCV Deformation Densities for the Interaction between Neutral Doublet [^t^Bu_3_PAu]· and [X]· Fragments (X = Al(NON′), B(*o*-tol)_2_) for Complexes **I** and **III′**^a^

	Δ*E*_oi_^1α^	CT^1α^	Δ*E*_oi_^1β^	CT^1β^	Δ*E*_oi_^2^	CT^2^
**I**	–32.7	–0.272	–24.5	0.299	–4.3	–0.030
**III′**	–57.5	–0.354	–24.8	0.296	–7.1	–0.064

a Data for **I** are taken and adapted with permission from ref (2). Copyright 2021 American Chemical Society.

From a qualitative perspective, the CD-NOCV curves displayed in Figure 1, together with the corresponding NOCV isosurfaces, point out that the [Al(NON′)]· and the [B(*o*-tol)_2_]· fragments form an overall qualitatively analogous bond with the [P^t^Bu_3_Au]· fragment. The Au–X bond consists mainly of two opposite charge transfers (CTs): an X-to-gold charge flux (Δρ_1α_′) and an inverse Au-to-X charge flux (Δρ_1β_′*).* On a quantitative ground, the Au–Al and Au–B bonds exhibit some small differences. Indeed, while the Δρ_1β_′ NOCV component is quantitatively similar for the two complexes (CT values are 0.299 and 0.296 e for **I** and **III′**, respectively, see also the overlapping corresponding curves in Figure 1), the magnitude of the Au-to-X charge transfer differs substantially. The boryl fragment is more capable of accepting charge from the gold moiety, resulting in a more negative CT value associated with the Δρ_1α_′ component with respect to the aluminyl fragment (CT values are −0.272 and −0.354 e for **I** and **III′**, respectively). The associated Δ*E*_oi_^*k*^ values vary accordingly: While the Δ*E*_oi_^1β^ values are comparable in the two cases (−24.5 and −24.8 for **I** and **III′**, respectively, see Table 1), the Δ*E*_oi_^1α^ component is almost twice as stabilizing for the boryl with respect to the aluminyl (−32.7 and −57.5 kcal/mol for **I** and **III′**, respectively). The enhanced ability of the boryl fragment of accepting charge from gold translates into a slightly reduced electron sharing character of the Au–B bond with respect to the Au–Al. This is substantiated by the molecular electronegativity of the fragments (Table S6 in the Supporting Information), which is higher for the boryl fragment than for the aluminyl (2.98 vs 2.54 eV), supporting the boryl’s higher tendency to form a more polarized Au(δ^+^)–B(δ^–^) bond.

The Δρ_2_′ component identifies a small dative Au-to-X π back-donation toward the valence empty *n*p_z_ orbital of B/Al (see Figure S3 in the Supporting Information for the corresponding isodensity pictures), and it highlights additional differences between the two systems. Both CT^2^ (−0.030 vs −0.064 e for **I** and **III′**, respectively) and Δ*E*_oi_^2^ values (−4.3 vs −7.1 kcal/mol for **I** and **III′**, respectively) clearly suggest a stronger Au-to-B π back-donation. Upon inspection of the acceptor molecular orbitals involved in this interaction (LUMO for the boryl and LUMO+1 for the aluminyl, see Figure S4 in the Supporting Information), the 2p_z_ orbital of B is more prone to be populated, as clearly indicated by the composition of the LUMO of the boryl fragment (more than 40% B 2p_z_ character, in contrast to a less than 25% contribution from the 3p_z_ orbital of Al for the LUMO+1 of the aluminyl fragment), with their energies varying accordingly (−2.7 vs −1.5 eV, respectively). Additionally, the different sizes of boron and aluminum atoms may play a significant role on the strength of this interaction. In particular, the smaller size of boron should favor a stronger interaction with oxygen, which is consistent with the larger contribution of the Δρ_2_′ component for complex **III′**. This is an interesting result in light of the reported significant role of the electrophilicity of the Al 3p_z_ orbital in the reactivity of **I** with CO_2_.^2^

Very importantly, the analyses of the Au–Al and Au–B bonds in **I** and **III′** complexes do not support evidence of (strongly) polarized Au(δ^–^)–Al(δ^+^) and Au(δ^–^)–B(δ^+^) bonds, which were supposed to be probed by the experimental observation of the nucleophilic behavior of gold in **I** and **III′**, resulting in Au–C and Al/B–O bonds in the carbon dioxide insertion products.^1,3^ Instead, the Au–B bonding picture in **III′** is consistent with an electron-sharing bond type, very much analogous to that of Au–Al in **I**, with a slightly larger polarization as Au(δ^+^)–B(δ^–^). This is also reflected in the binding picture that emerges when inspecting the IBOs for these complexes. For both **I** and **III′** (see Figures S5 and S8, respectively), five well-localized doubly occupied d-orbitals are identified alongside the two Au–X (X = B and Al) and Au–P bonds from the ligands. Analogously, a high electron sharing character is found for the Au–Al and Au–B bonds, as confirmed by the partial charge distributions (1.129/0.850 e on Al/Au, respectively, in **I** and 1.100/0.824 e on B/Au, respectively, in **III′**) for these bonds, which are fully consistent with the covalent and weakly polar Au−Al/B bonds.

### Aluminyl vs Boryl – [^t^Bu_3_PAu]: Effect on the Reaction Mechanism

**Figure 2:**
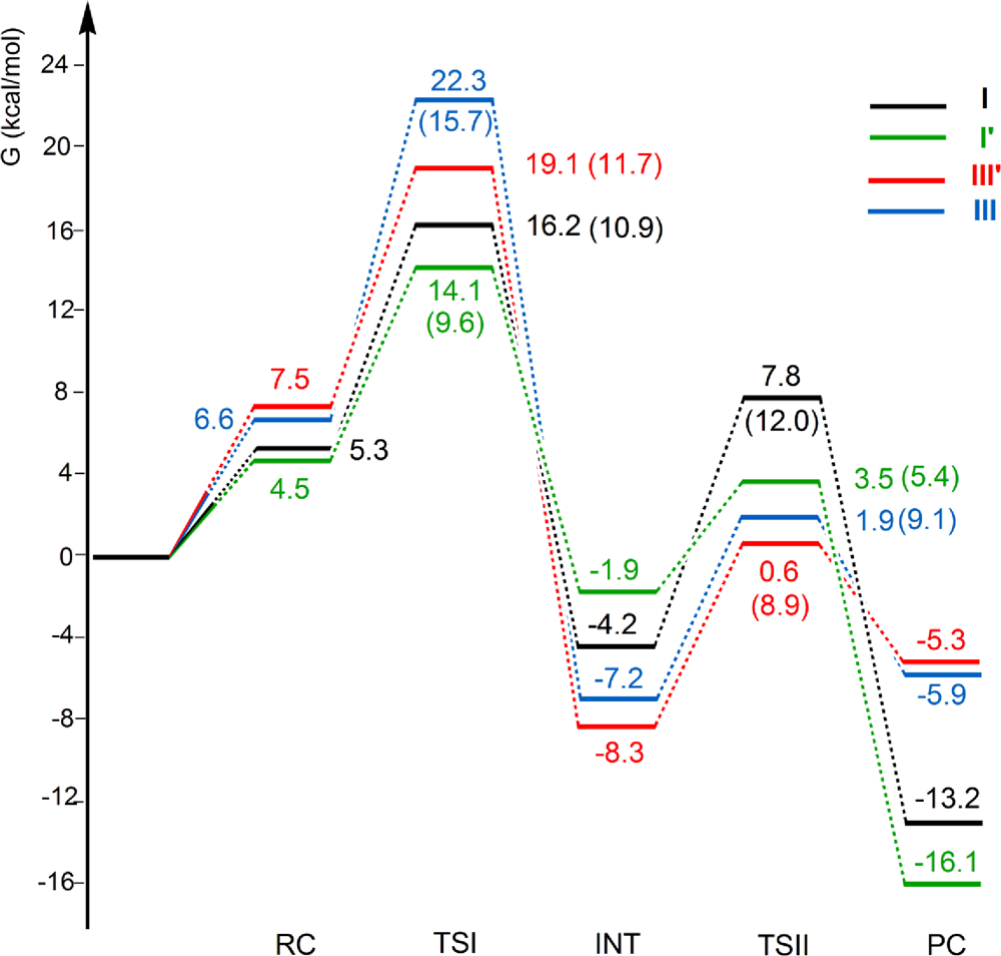
Free energy reaction profiles for the CO_2_ insertion into the Au–Al bond in the [^t^Bu_3_PAuAl(NON′)] complex **I** (black lines) and [IPrAuAl(NON′)] complex **I′** (green lines) and into the Au–B bond in the [IPrAuB(*o*-tol)_2_] complex **III** (blue lines) and [^t^Bu_3_PAuB(*o*-tol)_2_] complex **III′** (red lines). Δ*G* values refer to the energy of the separated reactants taken as zero. Activation free energy barriers are reported in parentheses. Results for **I** have been taken and adapted with permission from ref (2). Copyright 2021 American Chemical Society.

**Figure 3:**
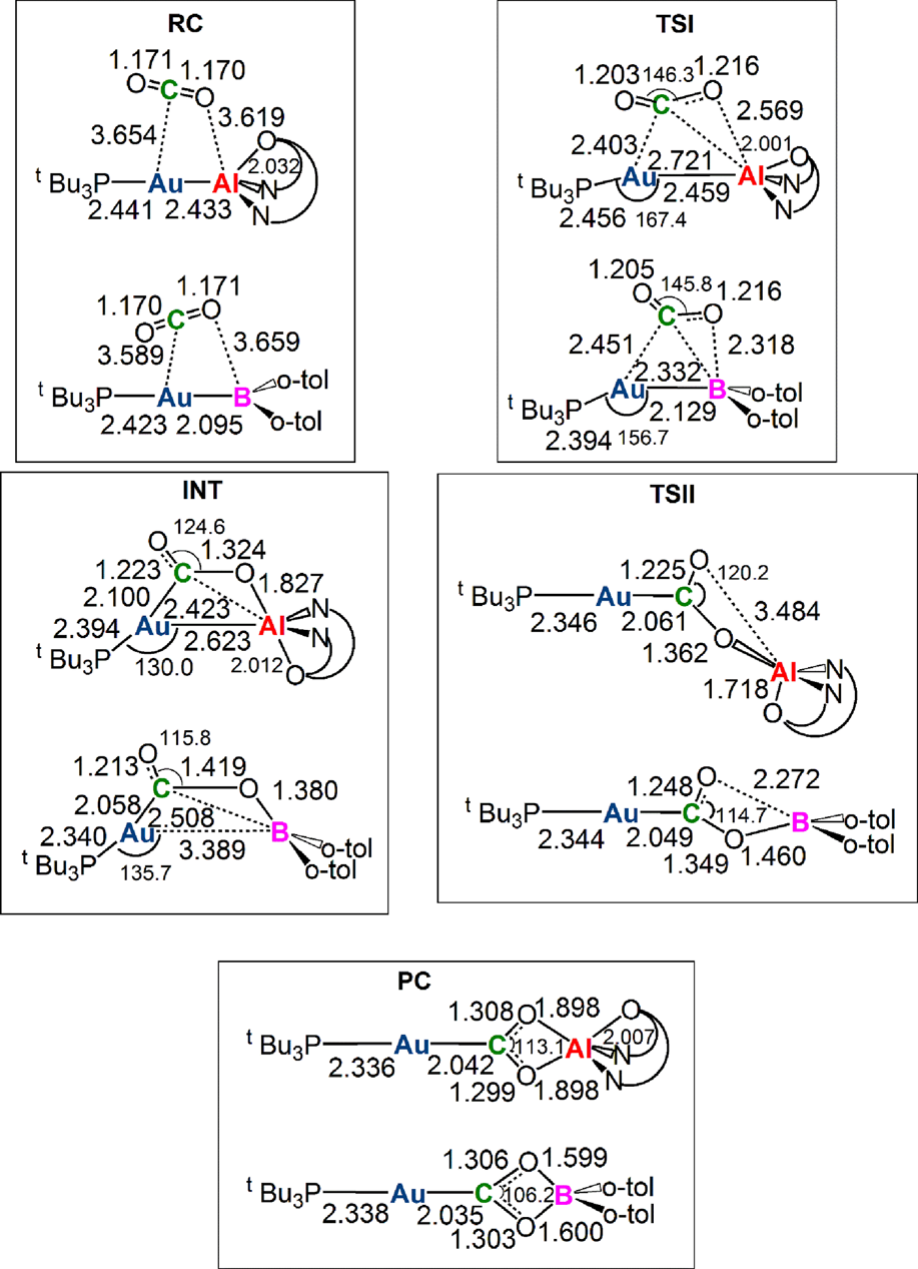
Sketched RC, TSI, INT, TSII, and PC structures for the [^t^Bu_3_PAuAl(NON′)] complex **I** and the [^t^Bu_3_PAuB(*o*-tol)_2_] complex **III′**. Selected interatomic distances (in Å) and bond angles (degrees) are given. Structures for **I** have been taken and adapted with permission from ref (2). Copyright 2021 American Chemical Society.

In this section, the mechanism for the CO_2_ reaction with the complexes under study is presented. The free energy profiles for the CO_2_ insertion into the Au–Al bond of complex **I** (taken from ref (2)) and the Au–B bond of **III′** are illustrated in Figure 2, together with those of **I′** and **III**, which will be discussed in the next section. Optimized structures of stationary points along the path for **I** and **III′** are also sketched with selected geometrical parameters in Figure 3, whereas fully optimized geometries are reported in the Supporting Information (Figures S9 and S10).

The reaction profiles depicted in Figure 2 for **I** and **III′** (black and red lines, respectively) are qualitatively very similar. In the first step, the nucleophilic attack to the CO_2_ carbon atom has a comparatively low activation free energy barrier (Δ*G*^≠^ = 10.9 and 11.7 kcal/mol for **I** and **III′**, respectively). The two TSI geometries are also very similar. In particular, a very similar bending of CO_2_ and asymmetry between the two C–O bonds can be observed for both complexes. Notably, however, one oxygen atom of CO_2_ is closer to B than to Al (2.318 Å vs 2.569 Å). Remarkably, since for complex **I**, a very flat potential energy surface (PES) around TSI has been observed^7^ and a concerted TSI is involved where more than two different molecular events are interlaced, the IRC approach fails here to probe the reaction pathway, precisely due to the complex PES topology (see Figure S11 in the Supporting Information and ref (22)).

Formation of intermediate INT is more favorable for boryl than aluminyl (27.4 kcal/mol vs 20.4 kcal/mol). We should note here that, for complex **I**, rotation of the [Al(NON)] Al–O bond in the pathway from TSI to INT is barrierless, as shown in Figures S12 and S13 in the Supporting Information. Inspection of INT structures and bond orders (BOs, see Table S7 in the Supporting Information) points out a first noticeable difference between the two systems. While the Au–Al bond length slightly increases (2.623 Å, BO 0.54), leading to a four-member (Au–C–O–Al) cyclic structure, the Au–B bond is substantially broken (3.389 Å, BO 0.07) and a larger bending of CO_2_ and asymmetry between the two C–O bonds is observed for complex **III′**. This is also consistent with the lower Au–X homolytic dissociation energy for **III′** with respect to **I** (77.2 vs 82.6 kcal/mol, respectively, see Table S8 in the Supporting Information) and with the reduced ability of B to achieve high coordination numbers with respect to Al. Indeed, upon coordination of the oxygen of CO_2_ to Al/B, we observe a cleavage of the Au–B bond, allowing the boron atom to maintain a three-coordinated structure and an sp^2^ hybridization (see Figure S14 in the Supporting Information). Conversely, in the case of complex **I**, a larger deviation from planarity in the initial complex is already seen, which evolves at INT with Al having a high coordination number, particularly since no Au–Al bond cleavage occurs.

To explain the difference between the two intermediate species of **I** and **III′**, both in structure and stability, we decompose the first part of the reaction path using the activation strain model (ASM) approach, which allows us to disentangle the contributions of the distortion of the reactants toward their in-adduct geometries and of their stabilizing interaction. The results of this analysis reveal that the larger stability of the intermediate of **III′** originates from a high distortion penalty, which is more efficiently counterbalanced by the stabilizing interactions between **III′** and CO_2_ with respect to **I** (Figure S15 and Tables S9 and S10 in the Supporting Information).

**Figure 4:**
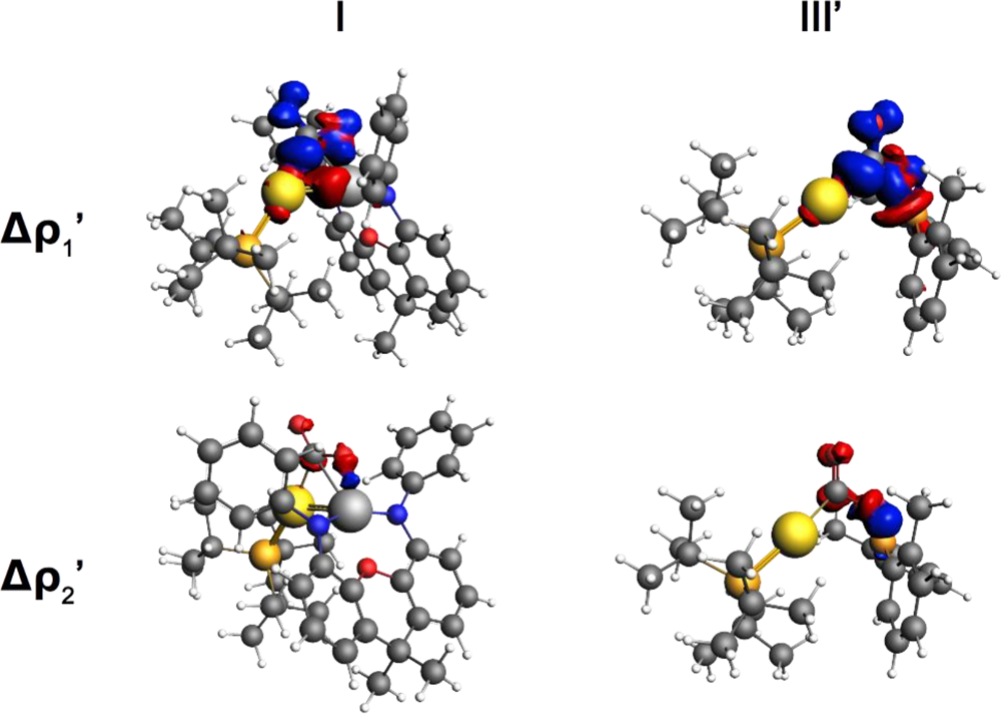
Isodensity surfaces associated with the Δρ_1_′ and Δρ_2_′ NOCV deformation densities for the intermediate INT structure of **I** (left column) and **III′** (right column). The charge flux is red-to-blue. The isodensity value is 5 me/a_0_^3^ for all surfaces.

Application of the ETS-NOCV approach to the TSI and INT structures allows us to get insights into the nature and extent of these stabilizing interactions. The isodensity pictures associated with the main interactions taking place at INT are shown in Figure 4. All the results of the ETS-NOCV analysis are reported in the Supporting Information (Tables S11 and S12 and Figures S16–S23).

The results of the ETS-NOCV analysis clearly indicate that the driving force of the first step of the reaction is qualitatively similar for the two systems. Both **I** and **III′** mainly interact with CO_2_ through electron donation from the Au–X bond into the LUMO of CO_2_ (Δρ_1_′, upper side in Figure 4), revealing that the nucleophilic character is captured in the Au–X bonds. This is further confirmed by the computation of the Fukui function and the dual descriptor^21^ for both complexes, which reveals that the nucleophilic character is identified in the Au–Al/B regions (see Figure S24 and Table S13 in the Supporting Information). This clearly indicates that it originates from the σ bond and can thus be expected to be released along the reaction coordinate.

In addition, electron donation from the HOMO of CO_2_ into the Al/B vacant valence atomic *n*p_z_ orbital is observed (Δρ_2_′, lower side; Figure 4). The stabilizing orbital interaction energy associated with Δρ_1_′ at the TSI is comparable (−41.2 and −42.5 kcal/mol for **I** and **III′**, respectively; Table S11), but at the INT, the interaction with CO_2_ is stronger for **III′** than for **I** (−389.3 vs −215.8 kcal/mol, respectively; Table S12), as also indicated by the corresponding calculated charge transfer (0.66 and 0.71 electrons transferred for **I** and **III′**, respectively). Remarkably, the orbital interaction associated with Δρ_2_′ is almost twice as large for **III′** already at TSI (−7.7 vs −4.0 kcal/mol), consistently with the strong oxophilicity of boron, the larger electrophilicity of the B 2p_z_ orbital, and, in general, the smaller size of boron and its orbitals (see the previous section). This difference becomes even more pronounced at INT, where both the orbital interaction energy and the CT value associated with Δρ_2_′ clearly point out a much stronger B–O interaction (−10.8 kcal/mol and 0.07 e for **I** and −47.1 kcal/mol and 0.18 e for **III′**, respectively; Table S12). Notably, for complex **I**, the orbitals involved in the interaction with CO_2_ do not reveal any contribution from the aluminyl Al–O σ* molecular orbital (see isodensity surfaces in Figure 4 and in Figures S16 and S17 in the Supporting Information), which is consistent with the high degree of flexibility of the [Al(NON)] ligand along the path.

The more stable INT structure of **III′** with respect to **I** can be rationalized in terms of three different features: (i) the greater lability of the Au–B bond, which, combined with the smaller size of B, allows the CO_2_ insertion to form an “open” insertion intermediate instead of a cyclic structure, as in **I**; (ii) the greater affinity of B for oxygen that allows the formation of a shorter and stronger B–O bond at the intermediate; and (iii) the greater electrophilicity of B due to the boryl LUMO nature (mainly a 2p_z_ orbital localized on boron). Indeed, inspection of the BOs highlights that, while the Al–O bond is weak at INT for **I** (BO = 0.22), the B–O bond for **III′** at INT already possesses a slight double-bond character (BO = 1.10).

The different nature of the intermediate for **I** and **III′** becomes even more clear in the second step of the reaction. The reaction proceeds *via* a INT rearrangement where an attack of the oxygen atom of CO_2_ to the electrophilic B/Al center occurs (the activation free energy barriers are 8.9 and 12.0 kcal/mol for **III′** and **I**, respectively), resulting in the formation of the insertion products **II** and **IV′** (PC in Figure 2). Despite the first step being thermodynamically favored for **III′**, the overall CO_2_ insertion is less exergonic for **III′** than for **I** (−5.3 vs −13.2 kcal/mol, respectively), and while **II** is more stable than the corresponding **INT** (ΔΔ*G* = −9.0 kcal/mol), the insertion product **IV′** is less stable (ΔΔ*G* = +3.0 kcal/mol). Noticeably, the INT-to-PC conversion is predicted to be endergonic for **III′**.

**Figure 5:**
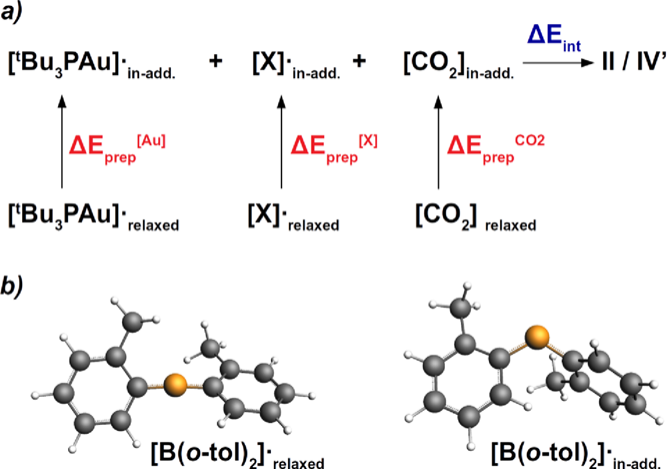
(a) Scheme for the formation of PCs **II**/**IV′** from [Al(NON′)]· and [B(*o*-tol)_2_]· radicals and CO_2_. (b) Geometries of the relaxed boryl radical (left) and the corresponding in-adduct geometry in **IV′** (right).

This difference in the second step can be explained by discussing the formation of **PC** in terms of the potential radical species involved, as already discussed in ref (2). Upon homolytic Au–X bond breaking, the two moieties are likely to display a radical-like behavior when forming the corresponding PC since the stability of the insertion product has been shown to be in relation with the stabilization induced by radical gold and aluminyl fragments.^2,7^ Here, we investigate the formation of the PC from the gold, aluminyl/boryl, and CO_2_ fragments. As reported in Table S14 and briefly discussed in the Supporting Information, the fragmentation of the PC into radical fragments appears to be the most convenient in this framework, thus supporting the radical-like behavior of the different moieties. On this basis, we study the formation of the CO_2_ insertion products according to the scheme reported in Figure 5a. The numerical results for **II** and **IV′** are shown in Table 2.

**Table 2:** Interaction Energy (Δ*E*_int_) and Preparation Energy of the [P^t^Bu_3_Au] (Δ*E*_prep_^[Au]^), Boryl/Aluminyl (Δ*E*_prep_^[Al]/[B]^), and CO_2_ (Δ*E*_prep_^CO2^) Fragments Considered for the Formation of PCs **II**/**IV′**^a^

	Δ*E*_int_	Δ*E*_prep_^CO2^	Δ*E*_prep_^[Al]/[B]^	Δ*E*_prep_^[Au]^	Δ*E*_prep_	Δ*E*
**I**	–200.7	94.4	0.1	0.3	94.8	–105.9
**III′**	–222.8	114.4	9.6	0.2	124.2	–98.5

a The overall preparation (Δ*E*_prep_) and formation (Δ*E*) energies are also reported. All energies are expressed in kcal/mol.

Based on the large oxophilicity (and electrophilicity) of the boryl fragment, one would expect the formation of **IV′** to be more favorable with respect to **II**. However, while the interaction energy (Δ*E*_int_) between the three fragments favors **IV′** over **II** (−222.8 vs −200.7 kcal/mol), the overall preparation energy (Δ*E*_prep_), *i.e.*, the energy required to distort the relaxed fragments to their in-adduct geometries, disfavors **IV′** (124.2 vs 94.8 kcal/mol), resulting in a more stabilizing formation energy Δ*E* for **II** (−105.9 vs −98.5 kcal/mol). A close inspection of the preparation energies associated to each fragment, apart from the most disfavoring contribution concerning CO_2_, due to the much distorted structure of CO_2_ in **IV′**, an additional penalty arises from the preparation energy for the boryl fragment (9.6 kcal/mol) since the relaxed geometry of the radical is substantially different, as it is shown in Figure 5b. While the in-adduct boryl fragment possesses a bent angular geometry, upon geometrical relaxation, the radical adopts an almost linear structure, with an sp hybridization on B that favors the delocalization of the unpaired electron into the (*o*-tol) substituents (see Figure S25 for the spin density distribution). This analysis unveils a really peculiar feature of boron in this type of reactivity. The sp^2^ hybridization of boron is essential for the first part of the reaction, where the readily available 2p_z_ orbital of B gets easily populated by CO_2_, resulting in a very stable intermediate. In the second step, however, the tendency toward sp^2^ hybridization appears to be unfavorable for the insertion product formation. Despite the great oxophilicity of boron, the tendency of the radical to undergo an sp hybridization and to delocalize the unpaired electron makes the boryl fragment less reactive toward the insertion of CO_2_, resulting in a less stable insertion product.

Investigation of the ligand (aluminyl vs boryl) effect on the Au–X bond and reaction mechanism for complexes **I′** and **III**, where the gold ligand is the N-heterocyclic carbene IPr ([IPrAu]), has been carried out within the same computational and methodological framework. Results are available in the Supporting Information (see Table S15 and Figures S26–S28) and are further discussed in the next sections.

### Phosphine vs Carbene – [Al(NON′)]: Gold Ligand Effect on the Au–Al Bond and Reaction Mechanism

The CD-NOCV results for the aluminyl complexes **I** and **I′** have been discussed in the previous section and in the Supporting Information where both the Au–Al bonds have been shown to be qualitatively described within the same electron-sharing, low-polar bonding picture. However, it is interesting to comparatively discuss the numerical results of the CD-NOCV bond analysis, which are reported in Table 3.

**Table 3:** Orbital Interaction Energies (Δ*E*_oi_^*k*^) (in kcal/mol) and Charge Transfer (CT^*k*^) (in Electrons, e) Associated with the First Two NOCV Deformation Densities for the Interaction between Neutral Doublet [LAu]· and [Al(NON′)]· Fragments (L = ^t^Bu_3_P, IPr) for Complexes **I** and **I′**^a^

	Δ*E*_oi_^1α^	CT^1α^	Δ*E*_oi_^1β^	CT^1β^	Δ*E*_oi_^2^	CT^2^
**I**	–32.7	–0.272	–24.5	0.299	–4.3	–0.030
**I′**	–33.6	–0.307	–24.2	0.275	–4.3	–0.046

a Data for **I** are taken and adapted with permission from ref (2). Copyright 2021 American Chemical Society.

Comparison between the two complexes is surprising, particularly considering that the two ancillary ligands, phosphine and carbene, commonly induce different electronic trans effects in “canonical” Au(I) complexes and, in general, in coordination chemistry and catalysis.^23−26^ In these unconventional complexes, however, this remarkable difference appears to be quenched. Based on the data shown in Table 3, we could safely say that the ligand effect on the Au–Al bond is almost negligible. The two main components of the Au–Al bond (Δρ_1α_′ and Δρ_1β_′) in **I** and **I′** only differ in terms of charge transfer (0.272 vs 0.307 e for Δρ_1α_′ and 0.299 vs 0.275 e for Δρ_1β_′) and stabilizing orbital interactions (−32.7 vs −33.6 kcal/mol for Δρ_1α_′ and −24.5 vs −24.2 kcal/mol for Δρ_1β_′) by fractions of electrons and of kcal/mol, respectively. Notably, also, the back-donation component Δρ_2_′ is overall similar in the two complexes in terms of orbital interaction energy (−4.3 kcal/mol for both **I** and **I′**). The IBO analysis corroborates this picture, confirming an electron-sharing Au–Al bond for I, which is negligibly affected by the different ancillary ligand at gold (partial charges related to the Au–Al bond are 1.120/0.768 e on Al/Au, respectively, see Figure S6 in the Supporting Information).

The free energy profiles for the CO_2_ insertion into the Au–Al bond of **I** and **I′** can be also compared in Figure 2 (black and green lines, respectively). As a consequence of the analogous features of the Au–Al bond, the first activation barrier is very similar for the two complexes in terms of Δ*G*^≠^ (10.9 and 9.6 kcal/mol for **I** and **I′**, respectively) and even closer in terms of Δ*E*^≠^ (9.0 and 8.6 kcal/mol for **I** and **I′**, respectively, see Table S9 and Figure S11). The effect of the gold ligand nature becomes, however, slightly more evident in the second step of the reaction, starting from a less stabilized INT species for complex **I′** (carbene-gold fragment) than that for complex **I** (phosphine-gold fragment). The reduced stability of the INT featuring the IPr ligand is consistent with the reduced Au–Al dissociation energy of **I** with respect to **I′** (82.6 vs 97.1 kcal/mol), resulting in a less advanced insertion of carbon dioxide into the bond. A slightly more stabilized PC complex for **I′** is formed (−16.1 vs −13.2 kcal/mol for **I′** and **I**, respectively) through transition state TSII, with Δ*G*^≠^ values amounting to 5.4 and 12.0 kcal/mol for **I′** and **I**, respectively. Thus, a moderate effect of the gold ancillary ligand can be detected only on the formation of the insertion product. By relying on the scheme shown in Figure 5a, we are able to rationalize this behavior again in terms of a radical-like reactivity, as shown by the data reported in Table 4.

**Table 4:** Interaction Energy (Δ*E*_int_) and Preparation Energy of the [LAu] (Δ*E*_prep_^[Au]^), Aluminyl (Δ*E*_prep_^[Al]^), and CO_2_ (Δ*E*_prep_^CO2^) Fragments Considered for the Formation of PCs **II**/**II′**^a^

	Δ*E*_int_	Δ*E*_prep_^CO2^	Δ*E*_prep_^[Al]^	Δ*E*_prep_^[Au]^	Δ*E*_prep_	Δ*E*
**I**	–200.7	94.4	0.1	0.3	94.8	–106.0
**I′**	–216.7	95.2	1.0	0.7	96.9	–119.8

a The overall preparation (Δ*E*_prep_) and formation (Δ*E*) energies are also reported. All energies are expressed in kcal/mol.

From Table 4, the preparation energy penalty (Δ*E*_prep_) does not influence the overall stability of the formed product. Instead, the stabilizing interaction between the in-adduct fragments favors **I′** over **I** (−216.7 vs −200.7 kcal/mol), resulting in an overall more favorable formation energy for **I′** (−119.8 vs −106.0 kcal/mol), coherently with the slightly more stabilized insertion product **II′**. The greater ability of the [IPrAu] fragment to stabilize the product can be explained in terms of localization of the spin density. As shown in Figure S21 in the Supporting Information, for the [^t^Bu_3_PAu]· fragment, the unpaired electron is more delocalized on the P atom (0.73 e on Au), whereas for the [IPrAu]·, it is more localized on the gold atom (0.86 e) (probably due to the more diffuse P 3sp than the C 2sp hybrid orbital, which is able to more efficiently delocalize the unpaired electron), which can be related to an increased reactivity of the [IPrAu] radical.

Overall, the comparative mechanistic study suggests an only moderate ligand influence on the reactivity, with a slightly beneficial effect of the [IPrAu] fragment for the CO_2_ insertion into the Au–Al bond in the aluminyl [LAuAl(NON′)] (L = IPr, ^t^Bu_3_P) complex. For the sake of completeness, we briefly explore the feasibility of the complete reduction of CO_2_ to CO and the possible ligand effect on this process. For complex **I**, we already reported that the reaction is highly unlikely to proceed to CO elimination (the resulting oxide complex [^t^Bu_3_PAuOAl(NON′)] [CO] has been calculated to be thermodynamically highly unstable with Δ*G* = 16.6 kcal/mol).^2^ For **I′**, the situation is very similar: The oxide complex [IPrAuOAl(NON′)] [CO] is calculated to be also highly unstable (Δ*G* = 13.7 kcal/mol), thus suggesting that the CO extrusion reaction is unfeasible and that a ligand control on the reactivity of the gold-aluminyl complex with carbon dioxide is not achievable.

### Phosphine vs Carbene – [B(*o*-tol)_2_]: Gold Ligand Effect on the Au–B Bond and Reaction Mechanism

The Au–B bonding features in **III** and **III′** have been discussed in the previous section (and the Supporting Information) of this work, and analogously to the Au–Al bond in **I** and **I′**, the nature of the Au–B bond is only negligibly influenced by the ancillary phosphine/carbene ligand of gold, as it is shown in Table 5.

**Table 5:** Orbital Interaction Energies (Δ*E*_oi_^*k*^) (in kcal/mol) and Charge Transfer (CT^*k*^) (in Electrons, e) Associated with the First Two NOCV Deformation Densities for the Interaction between Neutral Doublet [LAu]· and [B(*o*-tol)_2_]· Fragments (L = ^t^Bu_3_P, IPr) for Complexes **III** and **III′**

	Δ*E*_oi_^1α^	CT^1α^	Δ*E*_oi_^1β^	CT^1β^	CT^1^	Δ*E*_oi_^2^	CT^2^
**III**	–61.7	–0.325	–23.9	0.277	–0.048	–0.1	–7.8
**III′**	–57.5	–0.354	–24.8	0.296	–0.058	–0.1	–7.1

The CD-NOCV results reported in Table 5 point out that the Au–B bond in **III** and **III′** is only slightly different. The variability range of the dominant components Δρ_1α_′ and Δρ_1β_′ upon substitution of the ancillary ligand of gold is tight: Δ*E*_oi_^1α^ is slightly favored for **III** (−61.7 vs −57.5 kcal/mol), whereas Δ*E*_oi_^1β^ appears to be slightly favored for **III′** (−24.8 vs −23.9 kcal/mol). Overall, as it can be seen from the net charge transfer associated with these two components (CT^1^ = −0.048 and −0.058 e for **III** and **III′**, respectively), the two components are practically equivalent. This result holds also true for the π back-donation component (CT^2^ and Δ*E*_oi_^2^ values only differ by 0.010 e and 0.7 kcal/mol, respectively), confirming the absence of a significant ligand effect on the Au–B bond.

**Figure 6:**
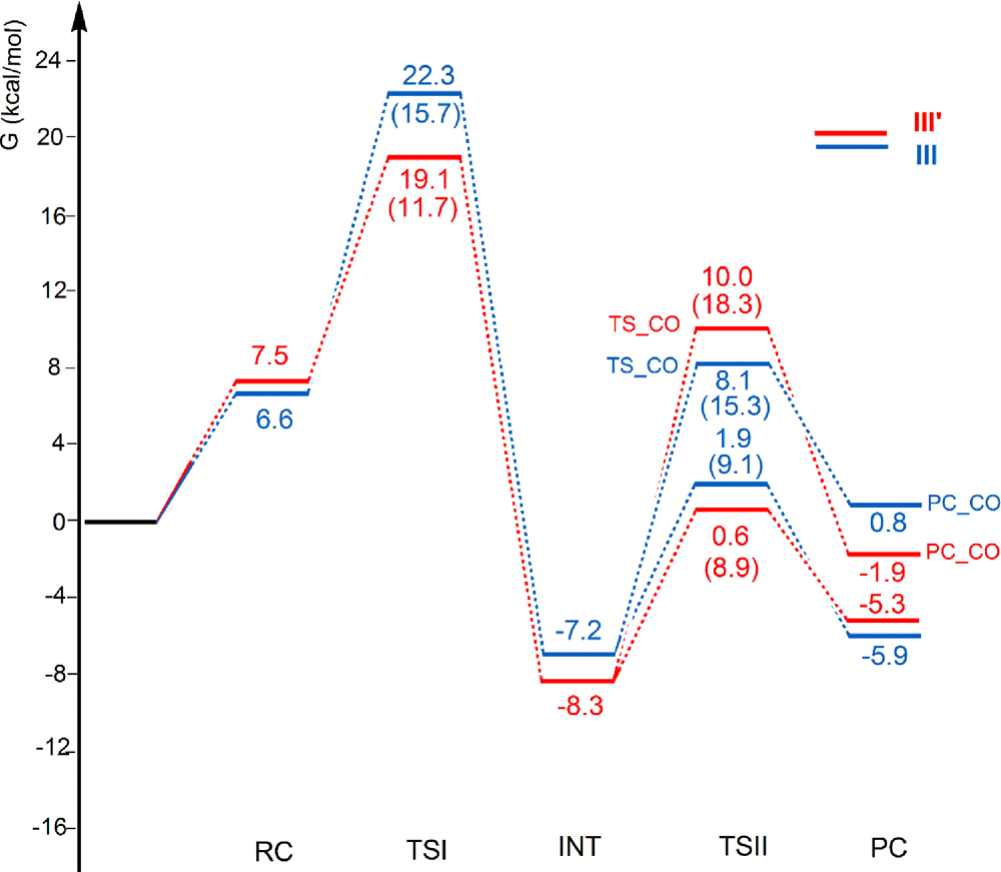
Free energy reaction profiles for the CO_2_ insertion into the Au–B bond in the experimental [IPrAuB(*o*-tol)_2_] complex **III** (blue lines) and in the model [^t^Bu_3_PAuB(*o*-tol)_2_] complex **III′** (red lines). Paths for CO extrusion are also shown (from INT to PC_CO *via* TS_CO). Δ*G* values refer to the energy of the separated reactants taken as zero. Activation free energy barriers are reported in parentheses.

The free energy profiles for the CO_2_ insertion into the Au–B bond of model complex **III′** and experimental complex **III** can be directly compared in Figure 6 (blue and red lines), where possible elimination of CO from the INT complex as an alternative route to PC formation has been explored for both complexes **III** and **III′** (TS_CO and PC_CO species in Figure 6).

Figure 6 shows that the Gibbs′ free energy activation barrier of the first step for **III** is larger than for **III′** (15.7 vs 11.7 kcal/mol, respectively), although the electronic activation energy barrier is very close (Δ*E*^≠^ 11.4 vs 11.7 kcal/mol for **III** and **III′**, respectively; Table S9). These findings, consistent with the negligible ligand effect on the Au–B bond (which acts as the nucleophile in this reaction step), suggest that no significant electronic effect can be observed in the first activation barrier. For boryls, the effect of the ligand on the second step of the reaction is even less significant: Starting from an only slightly more stable INT formed for **III′** (−8.3 kcal/mol) with respect to **III** (−7.2 kcal/mol), *via* a TSII with comparable activation barriers (9.1 vs 8.9 kcal/mol for **III′** and **III**, respectively), similarly stable insertion products PC are formed (−5.3 vs −5.9 kcal/mol for **III** and **III′**, respectively). Notably, although the INT-to-PC conversion is endergonic in both cases, it is slightly less unfavored for **III** (ΔΔ*G* = 1.3 kcal/mol) with respect to **III′** (ΔΔ*G* = 3.0 kcal/mol). This is consistent with the slightly enhanced affinity of the [IPrAu] radical for CO_2_, as shown in Table S14 in the Supporting Information. In both cases, it should be noticed that the reverse activation free energy barrier from PC to INT is sufficiently low to suggest that the formation of PC would be hardly observed under ambient conditions.

**Figure 7:**
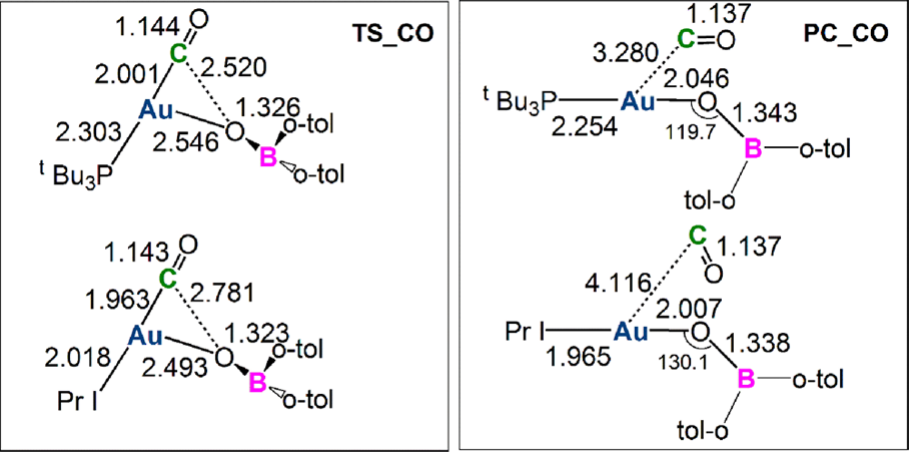
Sketched TS_CO and PC_CO structures for the [IPrAuB(*o*-tol)_2_] complex **III** and [^t^Bu_3_PAuB(*o*-tol)_2_] complex **III′**. Selected interatomic distances (in Å) and bond angles (degrees) are given.

Interestingly, while the oxide complexes (PC_CO species) for **I** and **I′** lie at a very high energy, as discussed in the previous section, [(L)AuOB(*o*-tol)_2_][CO] (L = ^t^Bu_3_P, IPr) complexes are more stabilized. Indeed, formation of PC_CO is almost thermoneutral for both **III** and **III′** (Δ*G* values are −1.9 and 0.8 kcal/mol for **III′** and **III**, respectively) and it proceeds with reasonable activation barriers *via* the transition state TS_CO (Δ*G*^≠^ values are 18.3 and 15.3 kcal/mol for **III** and **III′**, respectively). Optimized structures of TS_CO and PC-CO are sketched with the main geometrical parameters in Figure 7.

From Figure 7, we observe that the two TS_CO structures feature a partially formed Au–O bond (Au–O bond lengths are 2.546 and 2.493 Å for **III′** and **III**, respectively), a largely dissociated C–O bond (2.520 and 2.781 Å for **III′** and **III**, respectively), and a still short Au–C bond (2.001 and 1.963 Å for **III′** and **III**, respectively). The two PC_CO structures show an essentially dissociated CO and a formed [(L)AuOB(*o*-tol)_2_] oxide, where the boron atom presents a clear sp^2^ hybridization. The remarkably enhanced stability of boron-oxide complexes with respect to the aluminyl counterparts can be well explained in terms of the great oxophilicity of boron, and it can be observed by inspection of the PC_CO structures. Whereas for **III** and **III′** the B–O bonds are relatively short (1.338 and 1.343 Å for **III** and **III′**, respectively), with values that almost fall within the experimentally determined range of boron-oxide double bonds,^27−31^ the Al–O distances in **I** and **I′** (1.686 and 1.687 Å for **I** and **I′**, respectively, see Figures S9 and S28 in the Supporting Information) fall within the range of a single Al–O bond,^32^ clearly indicating the greater affinity of boron toward oxygen and rationalizing the relatively more stable PC_CO structures.

On comparing the free Gibbs energies for CO_2_ insertion product formation (PC) and for [(L)AuOB(*o*-tol)_2_] formation upon CO dissociation, *i.e.*, for equations [(L)AuB(*o*-tol)_2_] + CO_2_ → [(L)AuCO_2_B(*o*-tol)_2_] (1) and [(L)AuB(*o*-tol)_2_] + CO_2_ → [(L)AuOB(*o*-tol)_2_] + CO (2), we find Δ*G* (1) values of −5.9 and −5.3 kcal/mol and Δ*G* (2) values of −5.7 and −6.4 kcal/mol for complexes **III** and **III′**, respectively, thus suggesting that, thermodynamically, formation of the two products (PC and PC-CO) is competitive (and that [(L)AuOB(*o*-tol)_2_] species are more stable than the corresponding [LAuOAl(NON)] ones). However, formation of insertion products **IV** and **IV′** remains the favored path over the CO extrusion path, showing lower activation barriers.

Before concluding, we would like to point out that the steric hindrance of the gold ligand may be a crucial factor for the thermodynamics of the CO_2_ insertion. While exploring the reaction path for **III**, we have been able to optimize a conformational isomer of **IV** (**IV^isomer^**), with differently oriented isopropyl substituents on the IPr ligand (see Figure S29 for a comparison between the two structures). To our surprise, despite the very subtle structural difference between the **IV** isomers, the **IV^isomer^** lies at a much higher energy with respect to **IV** (Δ*G* = +5.4 vs −5.9 kcal/mol, see profiles in Figure S30), and as it can be seen by the buried volume (%V_bur_)-related steric maps^33^ (Figure S29 in the Supporting Information), the two ligands have a very differently distributed steric hindrance, which apparently results in a much less stable insertion product. To further assess this issue, we optimized the insertion product using a less hindered carbene ligand, namely, the ICy (ICy = 1,3-bis(cyclohexyl)imidazol-2-ylidene]). The use of this much less sterically hindered ligand resulted in an increased stability of the product with respect to both **IV** isomers (Δ*G* = −8.1 kcal/mol). These results clearly suggest that less sterically hindered NHC ligands may help to access more stable insertion products and call for a systematic investigation to properly and quantitatively address this interesting issue.

## Conclusions

The unconventional reactivity of a phosphine-gold-aluminyl complex toward carbon dioxide, with the formation of a CO_2_ insertion product featuring an Au-C(O_2_)-Al coordination mode, has been recently shown to be related to the unusual electron-rich and highly covalent Au–Al bond, which has been recognized as the nucleophilic site for the reaction, at a variance with the suggested nucleophilic behavior of the gold center. The formation of the insertion product has been also shown to occur through a radical-like mechanism. More recently, the reactivity of carbene-gold-diarylboryl complexes toward a series of C=N and C=O electrophiles, leading to the formation of Au–C and B–O/N bonds, similar to the “original” Au–Al complex, has been reported and a nucleophilic reactivity of the gold atom has been analogously suggested. These experimental findings have motivated us to investigate bonding and reactivity in gold-diarylboryl complexes. They also raise the question of the possible role of the gold ancillary ligand and anionic (aluminyl/diarylboryl) ligands in controlling the reactivity.

In this work, we computationally study the Au–Al/B bonding features, electronic structure, and carbon dioxide insertion reaction mechanism of four gold complexes with different anionic ligands (namely, the aluminyl Al(NON) and the diarlyboryl B(*o*-tol)_2_) and different gold ligands (namely, the phosphine ^t^Bu_3_P and the carbene IPr) to assess, if any, the Al/B and gold ligand effects on bonding, electronic structure, and reactivity.

The results show that boryl and aluminyl fragments form only slightly different covalent bonds with the gold fragment, which are responsible for a quantitatively different reactivity with CO_2_. While the Au–Al bond has an (non-polar) electron-sharing nature, the Au–B bond displays a slightly higher polarization as Au(δ^+^)–B(δ^–^), consistently with the ability of the boryl fragment to stabilize the negative charge. Concerning their reactivity, the greater oxophilicity (and electrophilicity) of boron is found to favor the formation of gold-boryl intermediate species in the first step of the reaction mechanism. However, in the second step, where the CO_2_ insertion product is formed, the reaction is found to be less favorable for boryls due to their decreased radical-like reactivity toward carbon dioxide.

For the gold ligand effect, we surprisingly find that, for both boryl and aluminyl-gold complexes, no evidence of a remarkable trans effect can be observed on both the Au–B and Au–Al bonds. As a result, the first step of the reaction is not affected by the gold ligand nature. In the second step, an only slight trans effect is found, with carbene ligands marginally favoring the formation of the CO_2_ insertion product. From an electronic perspective, the gold ligand effect is far from being remarkable in the complexes studied here. From a steric perspective, however, we find that the stability of the insertion product is extremely sensible to the steric hindrance of the gold ligand, with highly hindered ligands disfavoring the formation of stable products.

This work fits in the framework of a wider understanding and control of this remarkable and novel carbon dioxide reactivity with Au–X bonds, providing insights that may be useful for the efficient design of new and performing heterobimetallic complexes.

## Computational Details

All geometry optimizations and frequency calculations on the optimized structures (minima with zero imaginary frequencies and transition states with one imaginary frequency) for the CO_2_ insertion reaction into the [LAuX] (L = ^t^Bu_3_P, IPr ; X = Al(NON′), B(*o*-tol)_2_) complexes have been carried out using the Amsterdam density functional (ADF) code^34,35^ in combination with the related quantum-regions interconnected by local description (QUILD) program.^36^ The same modeling of the NON fragment (denoted NON′) has been used as that in ref (2), namely, the two *tert*-butyl groups at the peripheral positions of the dimethylxanthene moiety have been replaced with hydrogen atoms and the two Dipp substituents on the nitrogen atoms with phenyl groups. This modeling has been shown to give good agreement with available experimental geometrical data for complex **I** in ref (2). The PBE^37^ GGA exchange-correlation (XC) functional, the TZ2P basis set with a small frozen core approximation for all atoms, the ZORA Hamiltonian^38−40^ for treating scalar relativistic effects, and Grimme’s D3-BJ dispersion correction were used.^41,42^ Solvent effects were modeled by employing the conductor-like screening model (COSMO) with the default parameters for toluene as implemented in the ADF code.^43^ The same computational setup has also been used for the EDA, CD-NOCV, and ASM analyses and for computing the radical reactions between [X], [CO_2_], and [LAu] fragments. Mayer’s bond orders have been calculated with the same computational setup but relying on a larger (QZ4P) basis set. The calculation of conceptual DFT descriptors^21^ has been carried out by excluding solvent effects from the same computational protocol. The Fukui functions were calculated using the finite difference linearization approach. This setup has been successfully used in refs (1) and (2) to study the [^t^Bu_3_PAuAl(NON)] and [^t^Bu_3_PAuCO_2_Al(NON)] complexes. Intrinsic bond orbital (IBO)^20^ analyses were performed based on PBE-D3(BJ)/def2-SVP^44^ Kohn–Sham wavefunctions obtained from single-point calculation carried out using the electronic structure code ORCA (v4.2.1).^45,46^ Calculations were performed in the gas phase using Grid 5 and were accelerated using density fitting employing Weigend’s universal fitting basis sets.^47^ IBO analyses were performed using IboView.^48,49^ For further details and description of the methods used in this work, see the Methodology section in the Supporting Information.

## Funding

This work is funded by the Ministero dell’Università e della Ricerca (MUR, project AMIS, through the program “Dipartimenti di Eccellenza – 2018–2022”), the University of Perugia (“Fondo Ricerca di Base 2019“), the Netherlands Organisation for Scientific Research (NWO START-UP grant), and the Center for Information Technology of the University of Groningen.

## Supporting Information Available

Methodology; supplementary tables (comparative EDA, numerical results of the CD-NOCV analysis, conceptual DFT results, Mayer’s bond orders, hemolytic bond dissociation energies, ASM results for all complexes, EDA-NOCV results on TSI and INT for all complexes, results of the Fukui function localization, and formation energies of the insertion products from radical fragments); and figures (CD-NOCV curves for the Au–X bond and CD-NOCV isodensity surfaces, acceptor MOs in **I** and **III′**, optimized structures of stationary points along the reaction paths for all complexes, ASM diagrams, NOCV isosurfaces of the main interactions at TSI and INT for all complexes, isosurface plots of the dual descriptors, spin densities of the radical fragments in the product complexes, buried volume maps, and Gibbs’ energy profiles for Au–B complexes with differently hindered NHC ligands) (PDF)
*xyz* coordinates of all structures and movie file for vibrational modes associated with the imaginary frequency of the TSIs (ZIP)
