# Supplementary material for: Gold-Aluminyl and Gold-Diarylboryl Complexes: Bonding and Reactivity with Carbon Dioxide
Source: Inorg Chem. 2022 May 5;61(19):7327–37. doi: 10.1021/acs.inorgchem.2c00174 (PMC9115750; doi:10.1021/acs.inorgchem.2c00174)
Supplement: Supplementary file 1 — ic2c00174_si_001.pdf [file ic2c00174_si_001.pdf]

Gold-aluminyl and -diarylboryl complexes: bonding and reactivity with carbon dioxide

Diego Sorbelli,[*,†] Elisa Rossi,[†] Remco W.A. Havenith,[§a] Johannes E.M.N. Klein,[§] Leonardo Belpassi,[*,‡] and Paola Belanzoni[*,†,‡]

[†]*Department of Chemistry, Biology and Biotechnologies, University of Perugia, Via Elce di Sotto, 8 – 06123, Perugia, Italy* diegosorbelli00@gmail.com, paola.belanzoni@unipg.it

[‡]*CNR Institute of Chemical Science and Technologies "Giulio Natta" (CNR-SCITEC), Via Elce di Sotto, 8 – 06123, Perugia, Italy* leonardo.belpassi@cnr.it

[§]*Stratingh Institute for Chemistry, University of Groningen, Nijenborgh 4, 9747 AG Groningen, The Netherlands*

[a]*Zernike Institute for Advanced Materials, University of Groningen, Nijenborgh 4, 9747 AG Groningen, The Netherlands and Ghent Quantum Chemistry Group, Department of Chemistry, Ghent University, Krijgslaan 281 (S3), B-9000 Gent, Belgium*

Index of Contents

Methodology S2
Tables S1-S4. Comparative EDA S8
Figure S1. CD curves for the Au-X bond S10
Table S5. Results of the CD-NOCV analysis S11
Figures S2-S3. CD-NOCV isosurfaces S12
Figure S4. Acceptor MOs in **I** and **III'** S13
Table S6. Molecular electronegativity S14
Figures S5-S8. Relevant IBO for **I**, **I'**, **III**, and **III'** S15
Figures S9-S10. Optimized structures **I** and **III** S19
Table S7. Mayer Bond Orders S21
Table S8. Homolytic bond dissociation energies S21
Figures S11-S14. Reaction mechanism analysis S22
Tables S9-S10. ASM numerical data S25
Figure S15. ASM diagrams S26
Tables S11-S12. EDA-NOCV results on TSI and INT S27
Figures S16-S23. NOCV breakdown S28
Table S13. Local Fukui functions S36
Figure S24. Isosurface plots of the dual descriptor S36
Table S14. Formation energy of PC from fragments S37
Figure S25. Spin densities of [LAu] radicals S38
Figure S26. Sketched structures for **I'** and **III** S41
Figures S27-S28. Optimized structures **I'** and **III** S41
Figures S29. Steric maps, structures and profiles S44
Figure S30. Gibbs free energy profiles for differently hindered NHCs S45
References S46
xyz geometries S47

Methodology

- **Natural Orbitals for Chemical Valence and Charge Displacement analysis**

Natural Orbitals for Chemical Valence (NOCV)[1,2] represent a suitable approach for the description of chemical bonding based on the rearrangement of the electron density occurring when a chemical bond is formed. Such arrangement can be expressed as electron density difference between the formed adduct (AB) and sum of the densities of the two non-interacting fragments (A and B) frozen in their adduct geometry.

This deformation density can be brought into diagonal contributions in terms of NOCVs. In the NOCV scheme, the charge rearrangement taking place upon bond formation is obtained from the occupied orbitals of the two fragments suitably orthogonalized to each other and renormalized (*promolecule*). The resulting electron density rearrangement ($\Delta\rho'$) can be expressed in terms of NOCV pairs which are defined as the eigenfunctions of the so-called "valence operator"[3–5] as follows:

$$\Delta\rho' = \sum_k \nu_k(|\phi_{+k}|^2 - |\phi_{-k}|^2) = \sum_k \Delta\rho'_k \quad [S1]$$

where ϕ_{+k} and ϕ_{-k} are the NOCV pairs orbitals and $\nu_{\pm k}$ are the corresponding eigenvalues. When the adduct is formed from the promolecule, a fraction ν_k of electrons is transferred from the ϕ_{-k} to the ϕ_{-k} orbital, which are envisaged as donor and acceptor orbitals, respectively. For the sake of interpretation, a population analysis can also be performed in order to single out, for ϕ_{-k} and ϕ_{+k} orbitals, which molecular orbitals (MOs) of the two constituting fragments contribute to the interaction (with a resulting associated coefficient accounting for the magnitude of the contribution).

The NOCV scheme can be coupled with the framework of the Charge Displacement (CD)[6] analysis. The CD analysis allows to quantify the amount of electronic charge that is transferred between the two fragments upon the formation of the A-B bond. The Charge Displacement function (Δq) is defined as the partial progressive integration on a suitable z-axis of the deformation density $\Delta\rho'$:[7]

$$\Delta q(z) = \int_{-\infty}^{z} dz' \int_{-\infty}^{+\infty} \int_{-\infty}^{+\infty} \Delta\rho'(x, y, z')dxdy \quad [S2]$$

The CD function, $\Delta q(z)$, quantifies at each point of the bond axis the exact amount of electron charge that, upon formation of the bond, is transferred from the right to the left across a plane perpendicular to the bond axis through z.

When coupled with the NOCV scheme, the density rearrangement due to the bond formation between two fragments, ($\Delta\rho'$), can be partitioned in different NOCV deformation densities ($\Delta\rho'_k$) and therefore one is able to quantify the charge transfer (CT) associated to each different component. It must be noted that only few of the NOCV pairs contributes to the chemical bond. Therefore, when the CD-NOCV analysis is carried out, usually only the first $\Delta\rho_k'$ components are investigated in order to understand which significant chemical contribution to the bond they represent.

In equation [S2], the integration axis is usually conveniently chosen as the bond axis between the two fragments constituting the adduct and usually we choose to evaluate the charge transfer between A and B by taking the CD value at the "isodensity boundary", i.e. the z-point where equally valued isodensity surfaces of the isolated fragments become tangent.[7,8]

In this case, since we also apply this scheme to both TSI and INT, with [LAuX] (L= $^tBu_3P$, IPr ; X= Al(NON'), B(*o*-tol)$_2$) and [CO_2] as fragments, such approach is complicated, since the two fragments display multiple interactions with multiple atomic centres and thus it is clearly impossible to define a unique bond axis and it is very hard to rely on the isodensity boundary for the estimation of the charge transfer. In order to avoid any ambiguity in the definition of the z-axis, we recall an approach that may be useful for evaluating the charge transferred between the [LAuX] and [CO_2] fragments at TSI and INT.[9]

Within this approach, the electron density rearrangement ($\Delta\rho'$), which typically shows charge accumulation regions (positive values) and charge depletion regions (negative values), defines two different positive functions, $\Delta\rho^+$ and $\Delta\rho^-$, each equal to the magnitude of the appropriate portion, *i.e.*:

$$\Delta\rho^{+/-}(r) = max[\pm\Delta\rho(r)', 0] \quad [S3]$$

so that

$$\Delta\rho(r)' = \Delta\rho^+(r) - \Delta\rho^-(r) \quad [S4]$$

By defining two arbitrary regions that are associated with the interacting fragments, we can evaluate the charge transfer as follows:

$$CT = \int_A \Delta\,\rho(r)'dr = -\int_B \Delta\,\rho(r)'dr \quad [S5]$$

By combining Eqs. [S4] and [S5], CT can also be expressed as:

$$CT = \int_A \Delta\, \rho^{+}(r)dr - \int_A \Delta\, \rho^{-}(r)dr = -\int_B \Delta\, \rho^{+}(r)dr + \int_B \Delta\, \rho^{-}(r) \qquad [S6]$$

Ultimately, this approach can also be expressed in the CD-NOCV framework. By combining Equations [S1] and [S5], we can use to this approach for calculating the charge transfer associated to each NOCV deformation density as follows:

$$CT_k = \int_A \Delta\, \rho_k(r)'dr = -\int_B \Delta\, \rho_k(r)'dr \qquad [S7]$$

Despite the spatial regions associated to the two interacting fragments being defined arbitrarily, this approach is particularly suitable for the analysis of the interaction between the [LAuX] and [CO_2] fragments in TSI, being the two fragments well-separated in space.

- **Energy Decomposition Analysis and ETS-NOCV approach**

In this work the Energy Decomposition Analysis (EDA)[10,11] has been applied to get additional and complementary insights into the interaction between carbon dioxide and the [LAuX] (L= ^{t}Bu$_3$P, IPr ; X= Al(NON'), B(*o*-tol)$_2$) complex in the transition state TSI and intermediate INT. With this approach, the interaction energy between the [LAuX] and [CO_2] fragments can be decomposed in different contributions as follows:

$$\Delta E_{\text{int}} = \Delta E^{Pauli} + \Delta V_{elst} + \Delta E_{oi} + \Delta E_{disp} \qquad [S8]$$

where ΔE^{Pauli} represents the Pauli repulsion interaction between occupied orbitals on the two fragments, ΔV_{elst} is the quasiclassical electrostatic interaction between the unperturbed charge distribution of the fragments at their final positions, ΔE_{disp} takes into account the dispersion contribution and ΔE_{oi} is the orbital interaction, which arises from the orbital relaxation and the orbital mixing between the fragments, and accounts for electron pair bonding, charge transfer, and polarization.

The orbital interaction term ΔE_{oi} can be further decomposed within the ETS-NOCV[12] scheme into NOCV pairwise orbital contributions ($\Delta E_{oi} = \sum_k \Delta\, E_{oi}^k$) which associates an energy contribution (E_{oi}^k) to each NOCV deformation density ($\Delta\rho_k$).

- **Activation Strain Model**

The Activation Strain Model (ASM)[13–15] is a popular approach often used in order to get insights into the factors controlling the activation barrier of a process. Within this framework, the activation barrier ($\Delta E^{\#}$) can be decomposed as follows:

$$\Delta E^{\#} = \left[\Delta E_{\text{dist}}^{TSI} - \Delta E_{dist}^{RC}\right] + \left[\Delta E_{\text{int}}^{TSI} - \Delta E_{\text{int}}^{RC}\right] = \Delta\Delta E_{dist} + \Delta\Delta E_{\text{int}} \quad \text{[S9]}$$

where the "$\Delta E_{dist}{}^{TSI}$" and "$\Delta E_{dist}{}^{RC}$" terms represent the energy penalty due to the distortion of the fragments (i.e. [LAuX] and CO_2) constrained in the structures of the transition state (TSI) and the reactant complex (RC) respectively, whereas "$\Delta E_{int}{}^{TSI}$" and "$\Delta E_{int}{}^{RC}$" represent the interaction energies between the fragments (with the geometries constrained at the ones assumed in the TSI and RC, respectively) in the two structures. These terms can be grouped in the "$\Delta\Delta E_{dist}$" and "$\Delta\Delta E_{int}$" terms, that represent the overall distortion and interaction contributions to the activation barrier, respectively.

Additionally, we can also rearrange Equation [S9] in order to express the distortion contributions relatively to the two fragments as follows:

$$\begin{gathered} \Delta\Delta E_{\text{dist}} = E_{CO2}^{TS} - E_{CO2}^{RC} + E_{AuX}^{TS} - E_{AuX}^{RC} \\ \Delta\Delta E_{\text{dist}} = \Delta E_{\text{dist}}^{CO2} + \Delta E_{\text{dist}}^{AuX} \end{gathered} \quad \text{[S10]}$$

where "$\Delta E_{dist}{}^{CO2}$" represent the distortion penalty (or stabilization) due to CO_2 rearranging from its structure in the RC going into TSI and the "$\Delta E_{dist}{}^{AuX}$" term represents the same distortion contribution concerning the rearrangement of the [LAuX] complexes.

Since, for the sake of comparison, we analyzed in the detail the electronic structure of intermediates INT, in order to exploit the factors controlling the different degrees of stabilization, we extended the ASM to the latters as follows:

$$\Delta E^{\#} = \left[\Delta E_{\text{dist}}^{\text{INT}} - \Delta E_{dist}^{TSI}\right] + \left[\Delta E_{\text{int}}^{\text{INT}} - \Delta E_{\text{int}}^{TSI}\right] = \Delta\Delta E_{dist} + \Delta\Delta E_{\text{int}} \quad \text{[S11]}$$

$$\begin{gathered} \Delta\Delta E_{\text{dist}} = E_{CO2}^{\text{INT}} - E_{CO2}^{TSI} + E_{AuX}^{\text{INT}} - E_{AuX}^{\text{TSI}} \\ \Delta\Delta E_{\text{dist}} = \Delta E_{\text{dist}}^{CO2} + \Delta E_{\text{dist}}^{AuX} \end{gathered} \quad \text{[S12]}$$

where the "$\Delta E_{dist}{}^{INT}$" term represents the energy penalty due to the distortion of the fragments (i.e. [LAuX] and CO_2) constrained in the structures of the intermediate (INT), whereas "$\Delta E_{int}{}^{INT}$"

represents the interaction energy between the fragments (with the geometries constrained at the ones assumed in the INT).

Intrinsic Bond Orbitals

Self-consistent field methods like Kohn-Sham DFT or Hartree-Fock allow quantitative predictions of many physical properties. Unfortunately, this requires expanding their occupied orbitals $\varphi_i(r)$ using large and flexible basis sets (e. g. triple zeta or larger), which have too large a variational freedom to allow for a unique assignment of basis functions to the individual atoms they are placed on. Intrinsic Atomic Orbitals (IAOs)[16] overcome such problem. Indeed, they are built in a way that they constitute a minimal basis set of atomic core and valence orbitals (and therefore they can be uniquely assigned to a given atom), but they are polarized for taking into account the molecular context. IAOs ($\omega_p (r)$) are expressed as a linear expansion over the basis functions $\chi_\mu(r)$ of the full computational basis set:

$$\omega_\rho(r) = \sum_{\mu \in B_1} R_\rho^\mu \, \chi_\mu(r) \qquad \text{[S13]}$$

where B_1 is the full computational basis set and the coefficients R are determined so that they span the occupied MOs ($\varphi_i (r)$) of the SCF wavefunction, but they still resemble in the best possible way the free-atom atomic orbitals.

Since IAOs span the occupied MOs, one can express the latter as a linear combination over the IAOs:

$$\phi_i(r) = \sum_{\rho \in B} O_i^\rho \, \omega_\rho(r) \qquad \text{[S14]}$$

This formulation has several advantages. First of all, in this way, each MO can be split unambiguously in its atomic contributions and the occupation of each orbital of the atom can be defined uniquely. Moreover, this is a true LCAO, since in this case instead of combining basis functions, atomic orbitals are combined.

IBOs ($\phi_\iota(r)$) are obtained in a way that combines the definition given in Eq. [S14] with the fact that a Slater determinant is invariant to unitary rotations among its occupied orbitals:[16]

$$\phi_\iota(r) = \sum_j^{occ} \phi_j \, U_{ji} \qquad \text{[S15]}$$

where U_{ji} are the elements of a unitary matrix.

$\phi_i(r)$and $\phi_i(r)$look actually different, but the actual N-electron wavefunctions have the same physical meaning. For this reason, U_{ji} is computed in a way similar to what happens in the Pipek-Mezey localization procedure, so that the resulting orbital charge is spread on the minimum possible atomic centres.

This approach allows to analyze bonding in terms that recall chemical common sense, that is σ bonds, π bonds, lone-pairs, bond orbitals and so on. Nevertheless, since their definition is purely mathematical, they can be useful for the interpretation of exotic and unknown bonding schemes. Moreover, consistencies[16] are found with electronegativity values, C 1s core-level shifts, resonance substituent parameters (σ_R), Lewis structures and oxidation states of transition metal complexes, making the IBO method a suitable and efficient method for the bonding analysis.

	[^{t}Bu$_3$PAu]· - [Al(NON')]·	[^{t}Bu$_3$PAu]$^+$- [Al(NON')]$^-$	[^{t}Bu$_3$PAu]$^-$- [Al(NON')]$^+$
**ΔE$_{Pauli}$**	167.80	218.09	200.21
**ΔE$_{Elst}$**	-173.43	-283.48	-244.25
**ΔE$_{Steric}$**	-5.63	-65.39	-44.05
**ΔE$_{oi}$**	-71.51	-105.34	-171.09
**ΔE$_{disp}$**	-10.42	-10.42	-10.42
**ΔE**	-87.56	-181.14	-225.56

Table S1. Energy Decomposition Analysis (EDA) of the interaction energy between [^{t}Bu$_3$PAu] and [Al(NON')] fragments in the [^{t}Bu$_3$PAuAl(NON')] complex using different fragmentations, i.e. doublet open shell neutral fragments (first column), [^{t}Bu$_3$PAu]$^+$ singlet and [Al(NON')]$^-$ singlet fragments (second column) and [^{t}Bu$_3$PAu]$^-$ singlet and [Al(NON')]$^+$ singlet fragments (third column). Energies are reported in kcal/mol.

	[^{t}Bu$_3$PAu]· - [B(*o*-tol)$_2$]·	[^{t}Bu$_3$PAu]$^+$- [B(*o*-tol)$_2$]$^-$	[^{t}Bu$_3$PAu]$^-$-[B(*o*-tol)$_2$]$^+$
**ΔE$_{Pauli}$**	223.65	282.17	230.83
**ΔE$_{Elst}$**	-200.64	-342.29	-230.39
**ΔE$_{Steric}$**	23.01	-60.12	0.44
**ΔE$_{oi}$**	-104.11	-123.17	-228.07
**ΔE$_{disp}$**	-7.14	-7.14	-7.14
**ΔE**	-88.24	-190.43	-234.78

Table S2. Energy Decomposition Analysis (EDA) of the interaction energy between [^{t}Bu$_3$PAu] and [B(*o*-tol)$_2$] fragments in the [^{t}Bu$_3$PAuB(*o*-tol)$_2$] complex using different fragmentations, i.e. doublet open shell neutral fragments (first column), [^{t}Bu$_3$PAu]$^+$ singlet and [B(*o*-tol)$_2$]$^-$ singlet fragments (second column) and [^{t}Bu$_3$PAu]$^-$ singlet and [B(*o*-tol)$_2$]$^+$ singlet fragments (third column). Energies are reported in kcal/mol.

	[IPrAu]· - [Al(NON')]·	[IPrAu]$^+$- [Al(NON')]$^-$	[IPrAu]$^-$- [Al(NON')]$^+$
**ΔE$_{Pauli}$**	175.23	214.71	214.40
**ΔE$_{Elst}$**	-184.05	-282.12	-250.90
**ΔE$_{Steric}$**	-8.83	-67.41	-36.50
**ΔE$_{oi}$**	-73.36	-101.41	-190.63
**ΔE$_{disp}$**	-14.38	-14.38	-14.38
**ΔE**	-96.57	-183.20	-241.51

Table S3. Energy Decomposition Analysis (EDA) of the interaction energy between [IPrAu] and [Al(NON')] fragments in the [IPrAuAl(NON')] complex using different fragmentations, i.e. doublet open shell neutral fragments (first column), [IPrAu]$^+$ singlet and [Al(NON')]$^-$ singlet fragments (second column) and [IPrAu]$^-$ singlet and [Al(NON')]$^+$ singlet fragments (third column). Energies are reported in kcal/mol.

	$[IPrAu]^{\cdot}$ - $[B(o\text{-tol})_2]^{\cdot}$	$[IPrAu]^{+}$- $[B(o\text{-tol})_2]^{-}$	$[IPrAu]^{-}$-$[B(o\text{-tol})_2]^{+}$
**ΔE_{Pauli}**	236.92	280.11	241.62
**ΔE_{Elst}**	-214.59	-344.49	-234.04
**ΔE_{Steric}**	22.33	-64.38	7.58
**ΔE_{oi}**	-108.57	-118.16	-246.58
**ΔE_{disp}**	-12.62	-12.62	-12.62
**ΔE**	-98.86	-195.17	-251.62

Table S4. Energy Decomposition Analysis (EDA) of the interaction energy between [IPrAu] and $[B(o\text{-tol})_2]$ fragments in the $[IPrAuB(o\text{-tol})_2]$ complex using different fragmentations, i.e. doublet open shell neutral fragments (first column), $[IPrAu]^{+}$ singlet and $[B(o\text{-tol})_2]^{-}$ singlet fragments (second column) and $[IPrAu]^{-}$ singlet and $[B(o\text{-tol})_2]^{+}$ singlet fragments (third column). Energies are reported in kcal/mol.

As reported in Refs. [17,18] the most accurate fragmentation in NOCV and EDA decompositions consists of the one for which the less negative orbital interaction energy term (ΔE_{oi}) is observed, *i.e.* the one that describes the most stable fragments. Clearly, in this case, for all complexes, the best fragmentations consist of the use of radical doublet gold and boryl/aluminyl fragments, since it provides the less negative ΔE_{oi} term in each case.

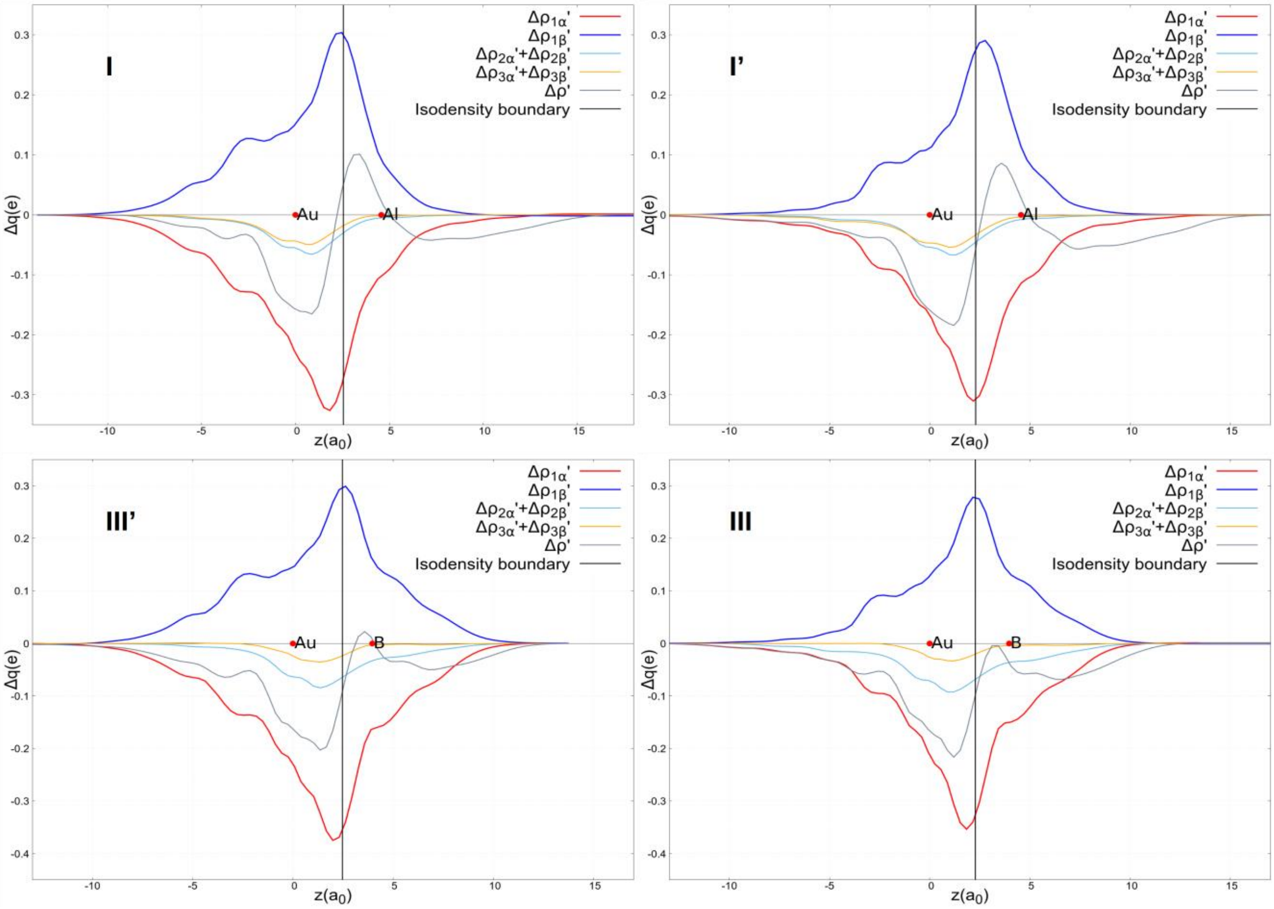

Figure S1. Charge displacement (CD-NOCV) curves for the interaction between the [^{t}Bu$_3$PAu] and [Al(NON')] (top left), [IPrAu] and [Al(NON')] (top right), [^{t}Bu$_3$PAu] and [B(*o*-tol)$_2$] (bottom left), [IPrAu] and [B(*o*-tol)$_2$] (bottom right) doublet fragments in complexes **I**, **I'**, **III**, **III'** respectively. Red dots indicate the position of the nuclei along the z axis. The vertical solid line marks the isodensity boundary between the fragments. Positive (negative) values of the curve indicate right-to-left (left-to-right) charge transfer.

$^{t}Bu_3PAuAl(NON')$ (I)						
**Net CT (e)**	**0.051**					
	**α**			**β**		
**k**	**$\|\nu_k\|$**	**ΔE_{oi}^{k} (kcal/mol)**	**CT^{k} (e)**	**$\|\nu_k\|$**	**ΔE_{oi}^{k} (kcal/mol)**	**CT^{k} (e)**
1	0.45	-32.66	-0.272	0.42	-24.49	0.299
2	0.09	-2.03	-0.015	0.09	-2.32	-0.015
**IPrAuAl(NON') (I')**						
**Net CT (e)**	**0.056**					
	**α**			**β**		
**k**	**$\|\nu_k\|$**	**ΔE_{oi}^{k} (kcal/mol)**	**CT^{k} (e)**	**$\|\nu_k\|$**	**ΔE_{oi}^{k} (kcal/mol)**	**CT^{k} (e)**
1	0.44	-33.63	-0.275	0.39	-24.19	0.307
2	0.09	-2.06	-0.023	0.08	-2.24	-0.023
**$^{t}Bu_3PAuB(\textit{o}\text{-tol})_2$ (III')**						
**Net CT (e)**	**-0.098**					
	**α**			**β**		
**k**	**$\|\nu_k\|$**	**ΔE_{oi}^{k} (kcal/mol)**	**CT^{k} (e)**	**$\|\nu_k\|$**	**ΔE_{oi}^{k} (kcal/mol)**	**CT^{k} (e)**
1	0.53	-61.66	-0.325	0.39	-23.88	0.277
2	0.14	-3.76	-0.034	0.14	-4.01	-0.034
**$IPrAuB(\textit{o}\text{-tol})_2$ (III)**						
**Net CT (e)**	**-0.091**					
	**α**			**β**		
**k**	**$\|\nu_k\|$**	**ΔE_{oi}^{k} (kcal/mol)**	**CT^{k} (e)**	**$\|\nu_k\|$**	**ΔE_{oi}^{k} (kcal/mol)**	**CT^{k} (e)**
1	0.54	-57.46	-0.354	0.42	-24.77	0.296
2	0.13	-3.44	-0.032	0.13	-3.70	-0.032

Table S5. Eigenvalues ($|\nu_k|$), orbital interaction energies (ΔE_{oi}^{k}) and charge transfer (CT_k) associated to the first three NOCV deformation densities (k=1-3) and to the corresponding α and β components for complexes **I**, **I'**, **III** and **III'**.

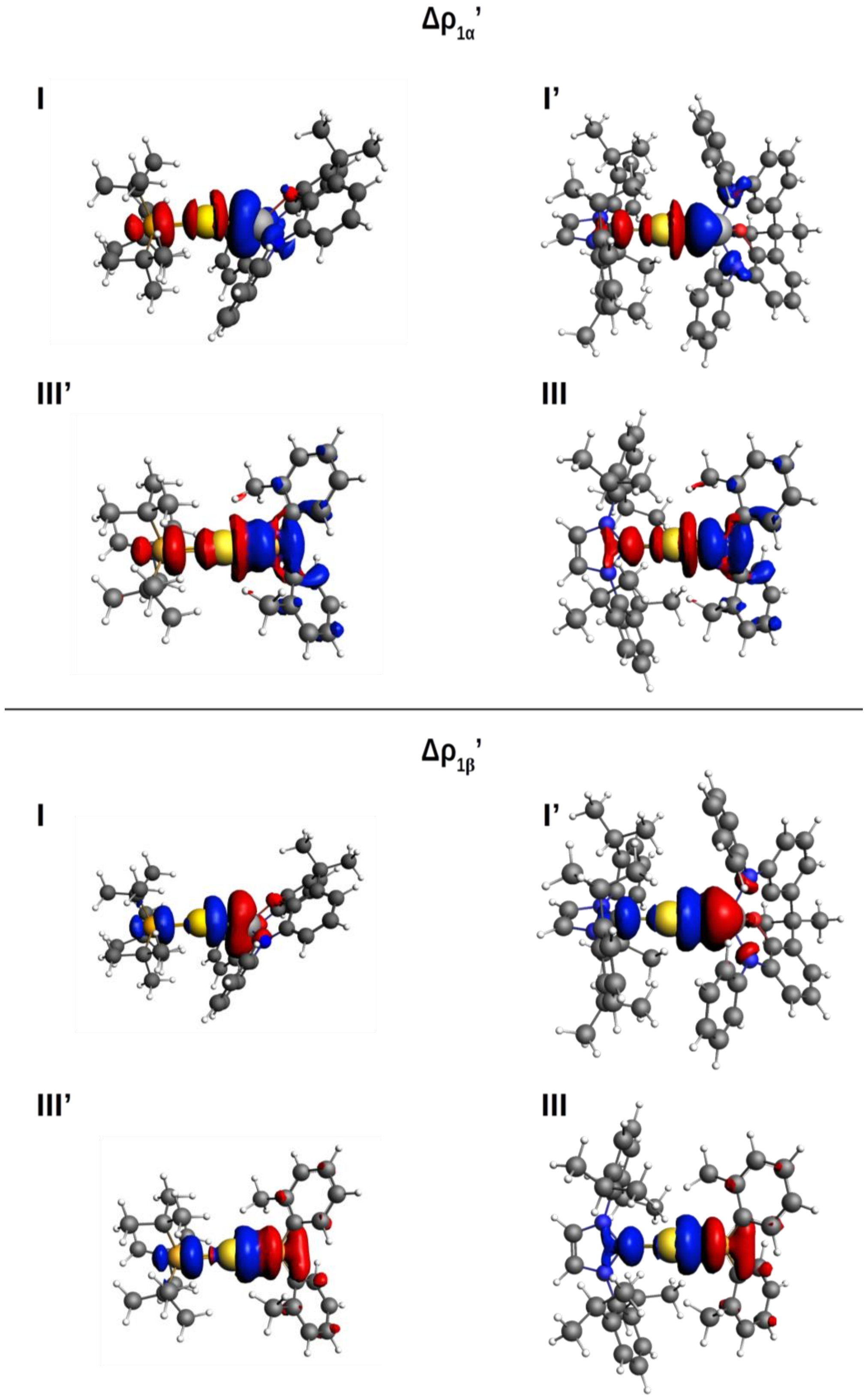

Figure S2. Isodensity surfaces of the *Δρ1α'* and *Δρ1β'* NOCV deformation densities for complexes **I**, **I'**, **III**, **III'**. Blue regions indicate electron density accumulation areas, whereas red regions indicate depletion areas. Isovalue for all surfaces is 1 me/a_0.

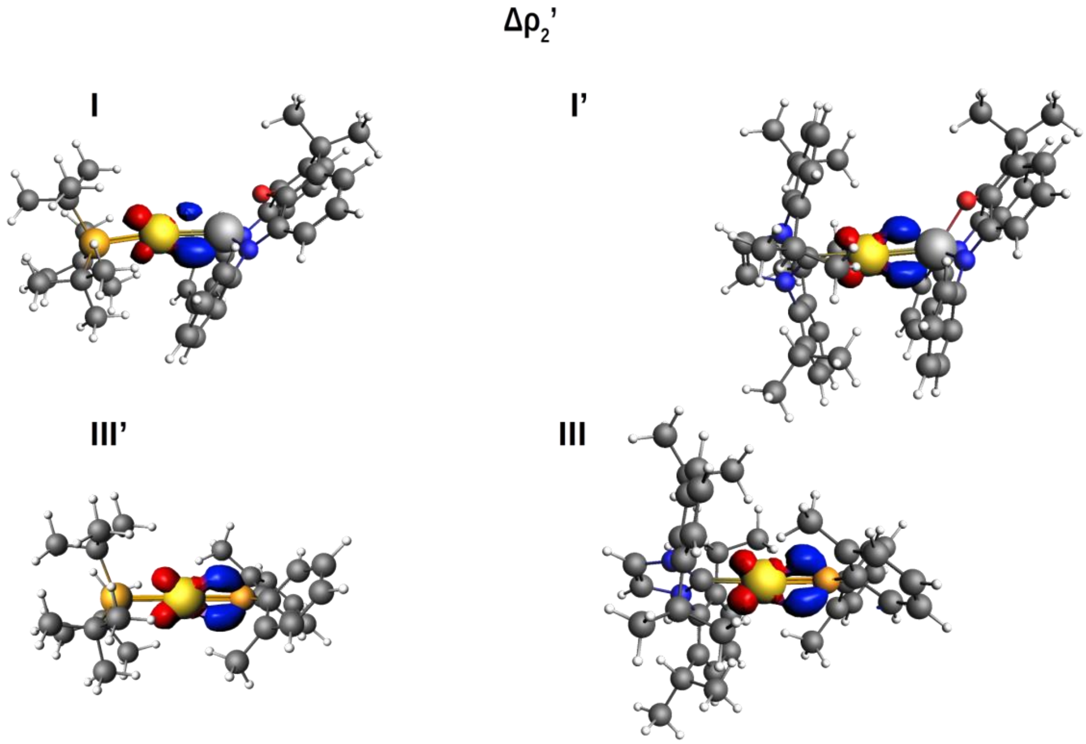

Figure S3. Isodensity surfaces of the $\Delta\rho_2$' and $\Delta\rho_3$' NOCV deformation densities for complexes **I**, **I'**, **III**, **III'**. Blue regions indicate electron density accumulation areas, whereas red regions indicate depletion areas. Isovalue for all surfaces is 1 me/a_0.

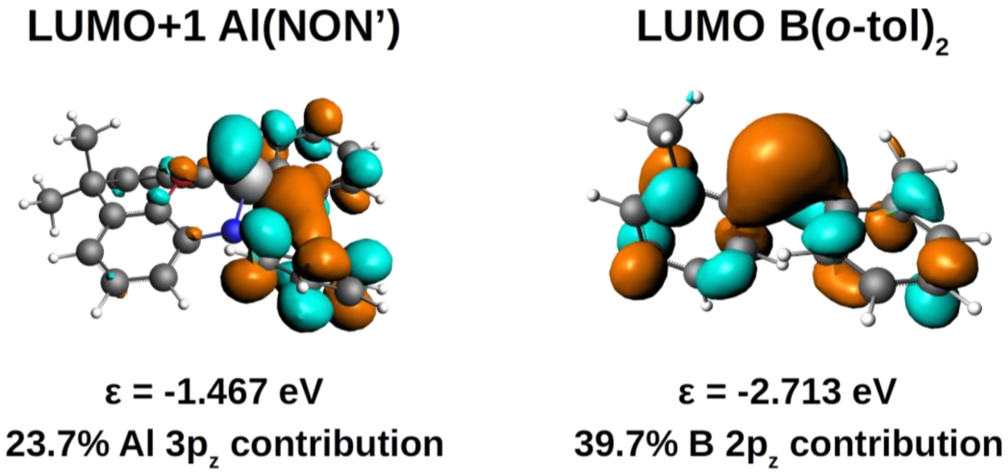

Figure S4. Isodensity surfaces of the main acceptor molecular orbitals for the Al(NON') (LUMO+1, left) and B(*o*-tol)$_2$ (LUMO, right) fragments involved in the *$\Delta\rho_2$'* bond component (Au-to-X back-donation) for **I** and **III'** respectively. The energy of the MOs and the contribution of the np_z atomic orbital of Al and B respectively are reported. Isovalue for all surfaces is 30 me/a_0.

	I		I’		III’		III	
	**[Au]**	**[Al]**	**[Au]**	**[Al]**	**[Au]**	**[B]**	**[Au]**	**[B]**
**IE**	5.40	6.33	5.17	6.35	5.41	6.79	5.16	6.74
**EA**	-0.26	-1.26	-0.03	-1.33	-0.27	-0.82	-0.02	-0.86
χ_M	2.57	2.54	2.57	2.51	2.57	2.98	2.57	2.94

Table S6. Ionization energies (IE), electron affinity (EA) and “molecular electronegativity” (χ_M, defined as χ_M=IE+EA/2) for the [LAu] (L=^{t}Bu$_3$P, IPr) and [X] (X=Al(NON’), B(*o*-tol)$_2$) neutral fragments in complexes **I**, **I’**, **III**, **III’**. The fragments have been kept frozen at their in-adduct geometries. Descriptors are expressed in eV.

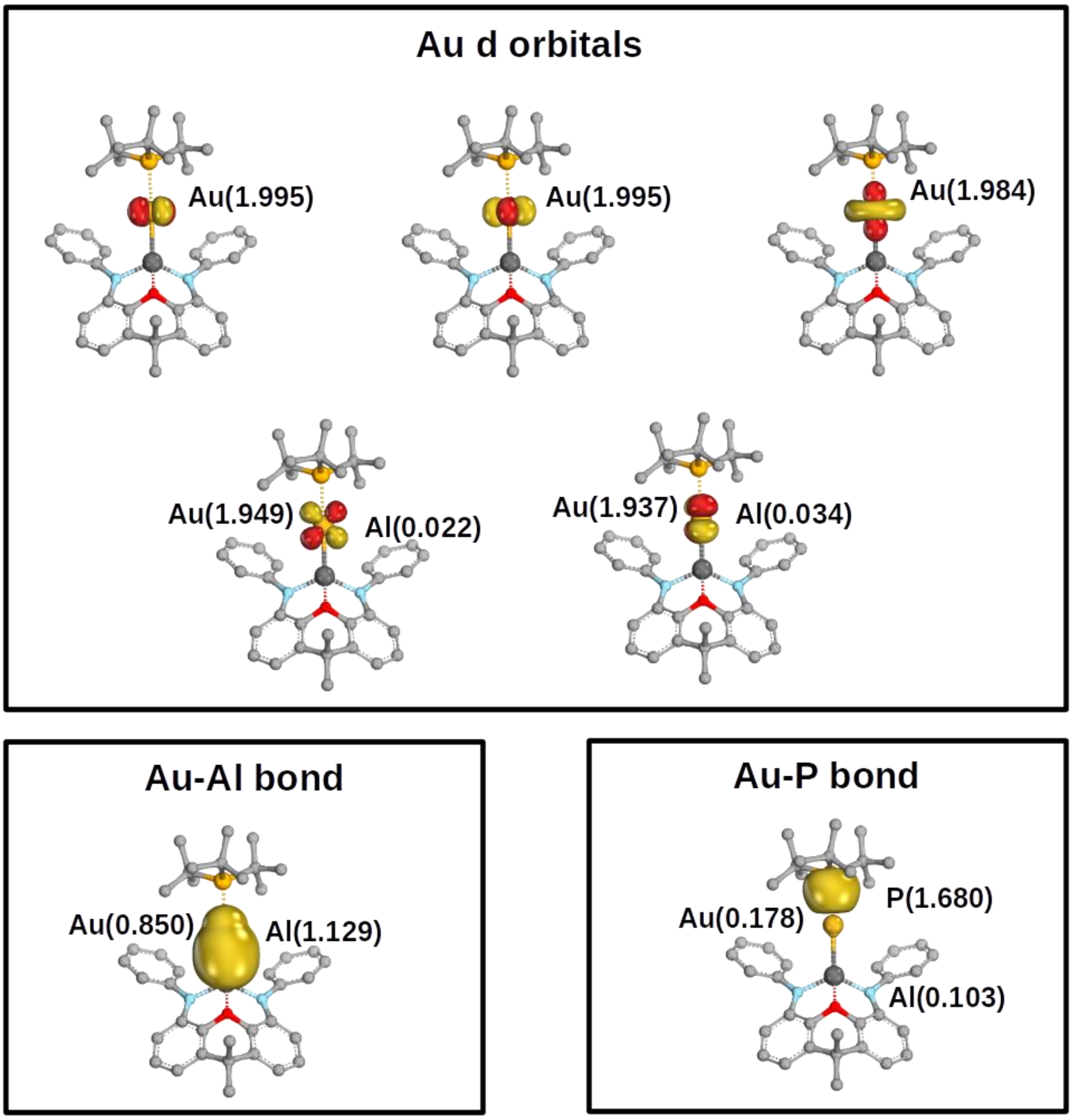

Figure S5. Relevant Intrinsic Bond Orbitals (IBOs) for **I**. Values in parentheses refer to the partial charge distribution on the related atoms.

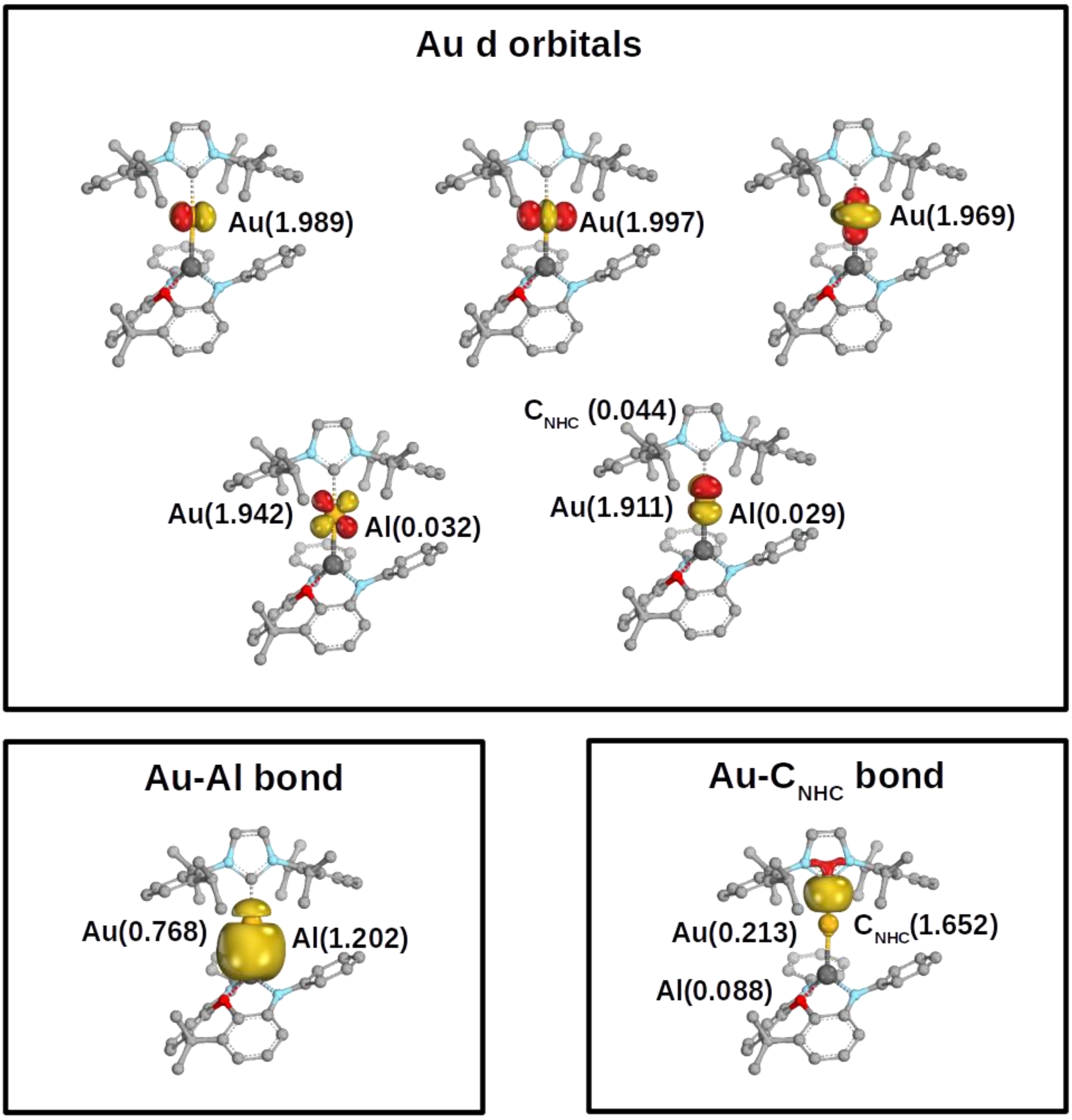

Figure S6. Relevant Intrinsic Bond Orbitals (IBOs) for **I'**. Values in parentheses refer to the partial charge distribution on the related atoms.

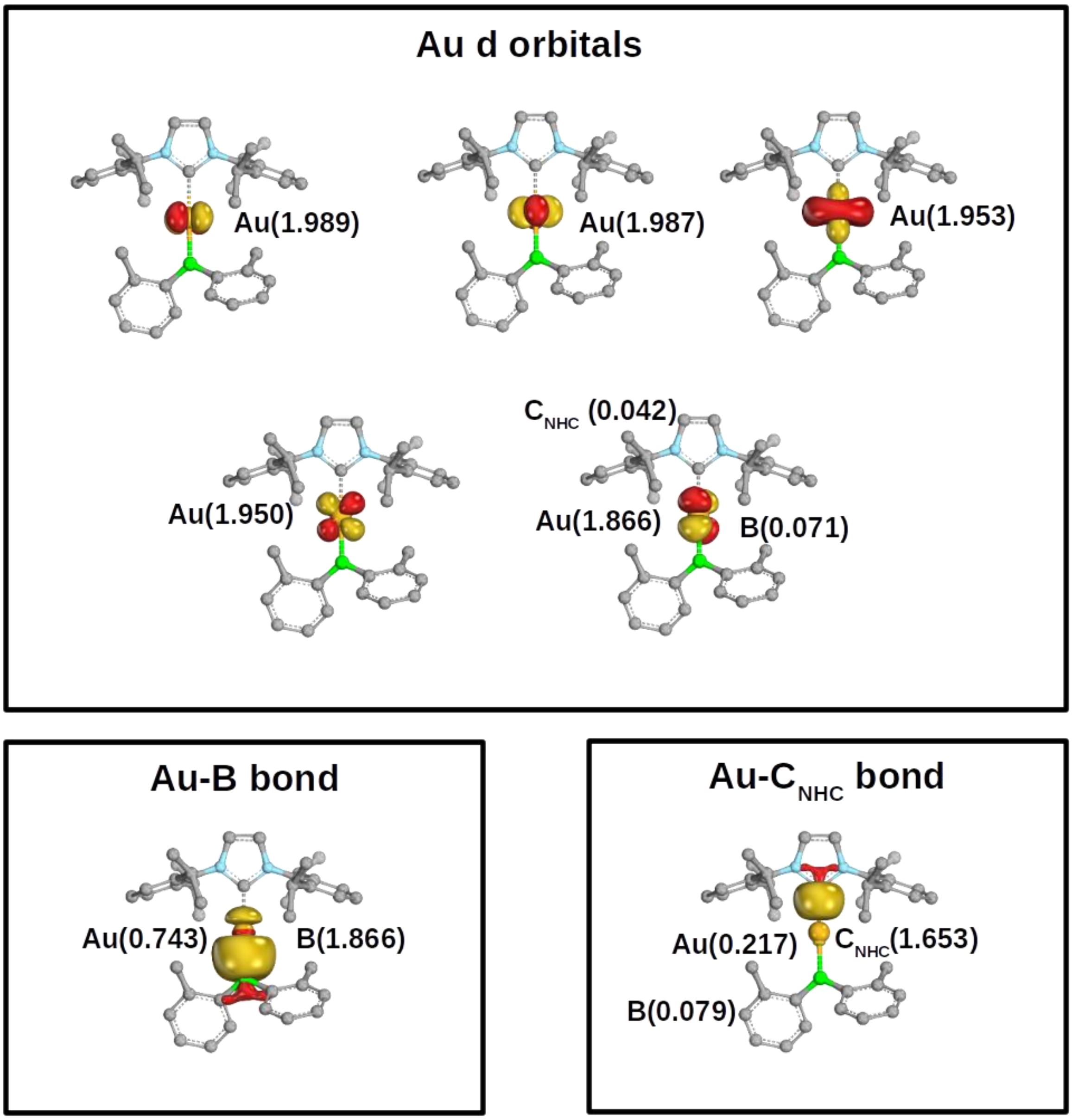

Figure S7. Relevant Intrinsic Bond Orbitals (IBOs) for **III**. Values in parentheses refer to the partial charge distribution on the related atoms.

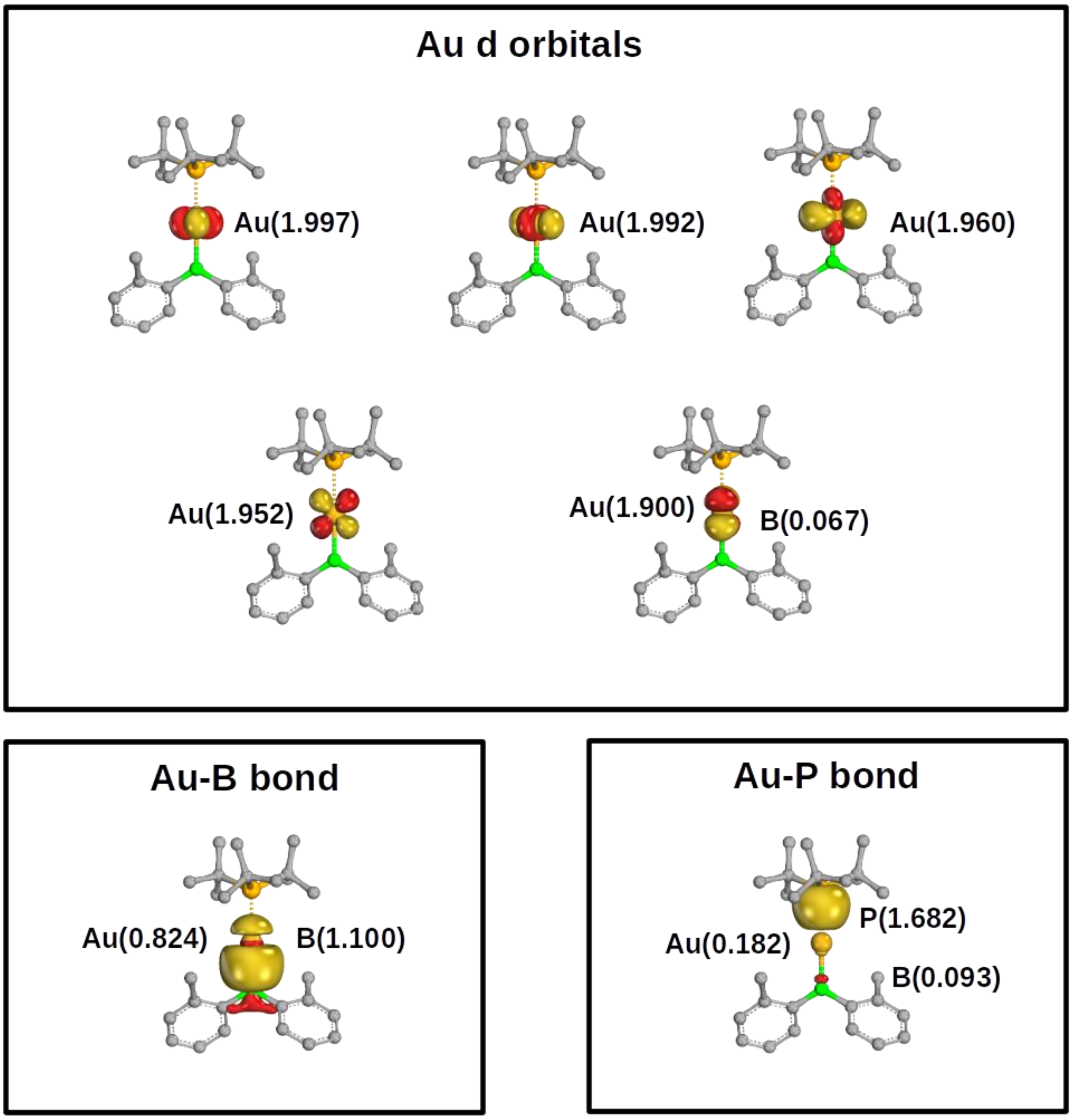

Figure S8. Relevant Intrinsic Bond Orbitals (IBOs) for **III'**. Values in parentheses refer to the partial charge distribution on the related atoms.

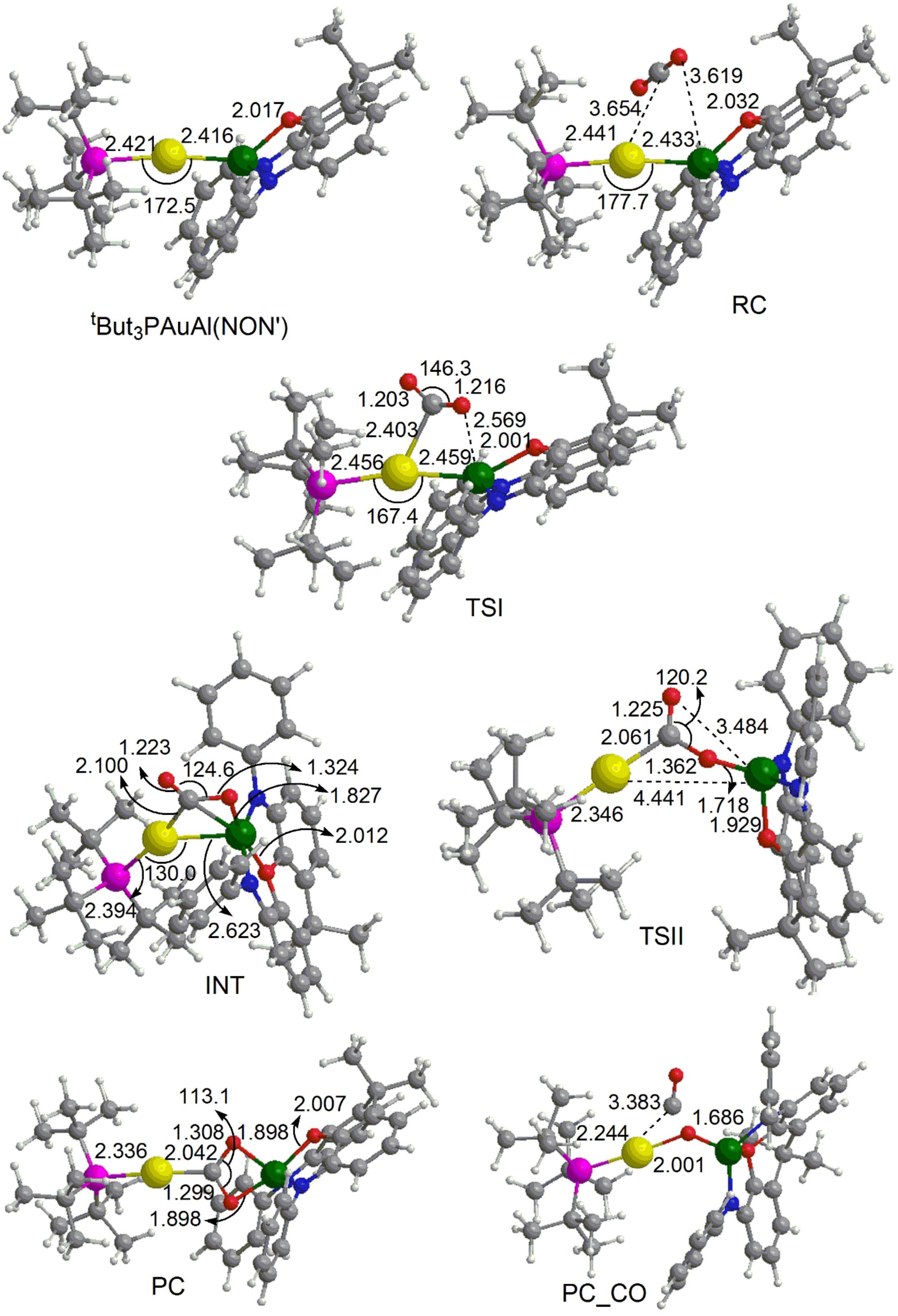

Figure S9. Optimized structures of [$^tBu_3PAuAl(NON')$] (complex **I**), RC, TSI, INT, TSII, PC and PC_CO complexes. Main geometrical parameters are reported (bond in Å, angles in degree).

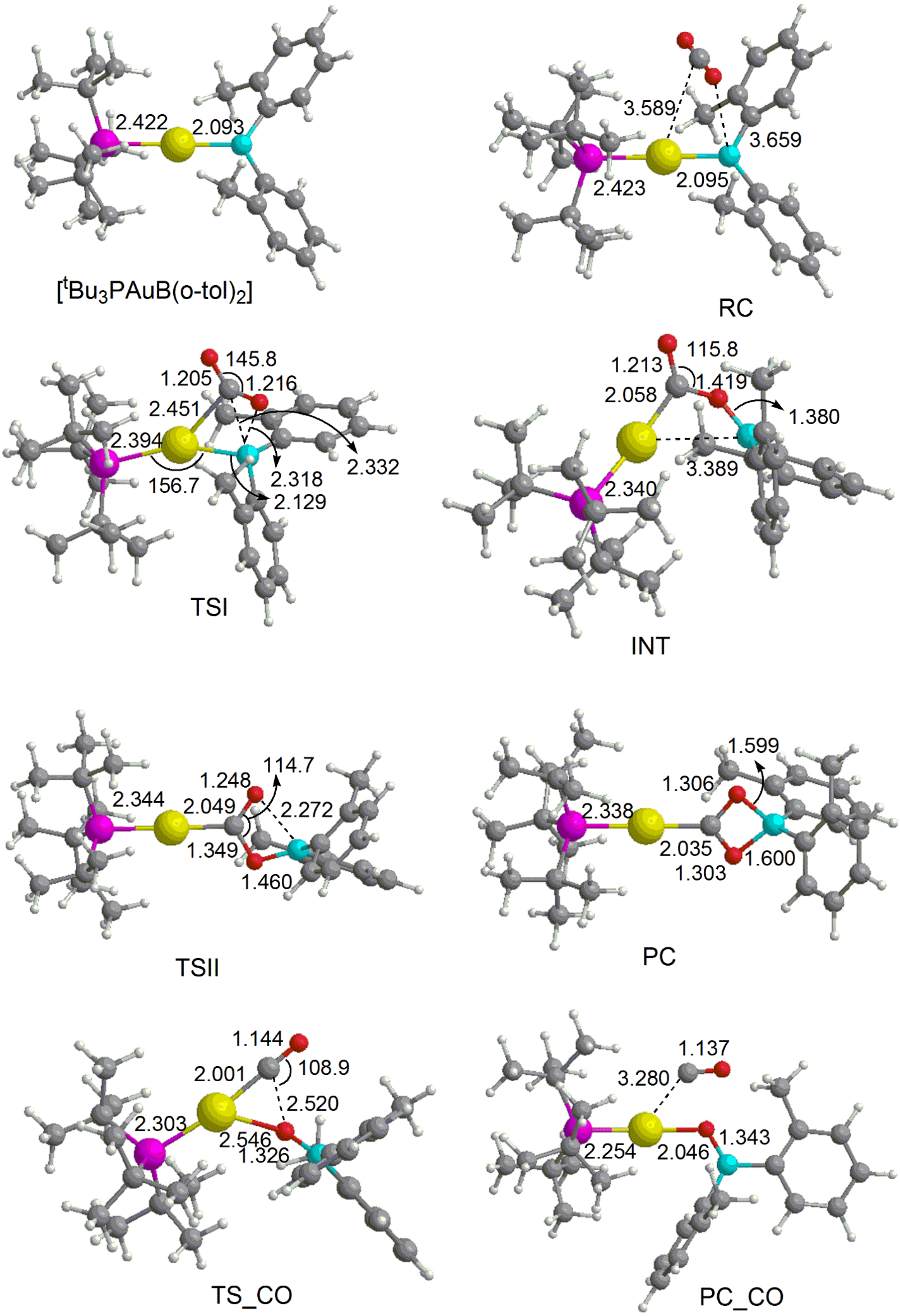

Figure S10. Optimized structures of [^{t}Bu$_3$PAuB(o-tol)$_2$] (complex **III'**), RC, TSI, INT, TSII, PC, TS_CO and PC_CO complexes. Main geometrical parameters are reported (bond in Å, angles in degree).

Bond order	I	III'	I'	III
Au-X	0.54	0.07	0.39	0.05
X-O^1_{CO2}	0.22	1.10	0.01	1.19
C-O^1_{CO2}	1.40	0.95	1.42	0.87
C-O^2_{CO2}	1.79	1.81	1.83	1.74

Table S7. Mayer's bond orders relative to INT structures of the most relevant interactions for complexes **I**, **I'**, **III**, **III'** respectively. Only bond orders greater than 0.1 are reported.

	I	I'	III	III'
Homolytic D_e	82.6	97.1	94.2	77.2

Table S8. Homolytic dissociation energies (D_e) for the Au-X (X=B, Al) bond in complexes **I**, **I'**, **III**, **III'** respectively. Energies are reported in kcal/mol.

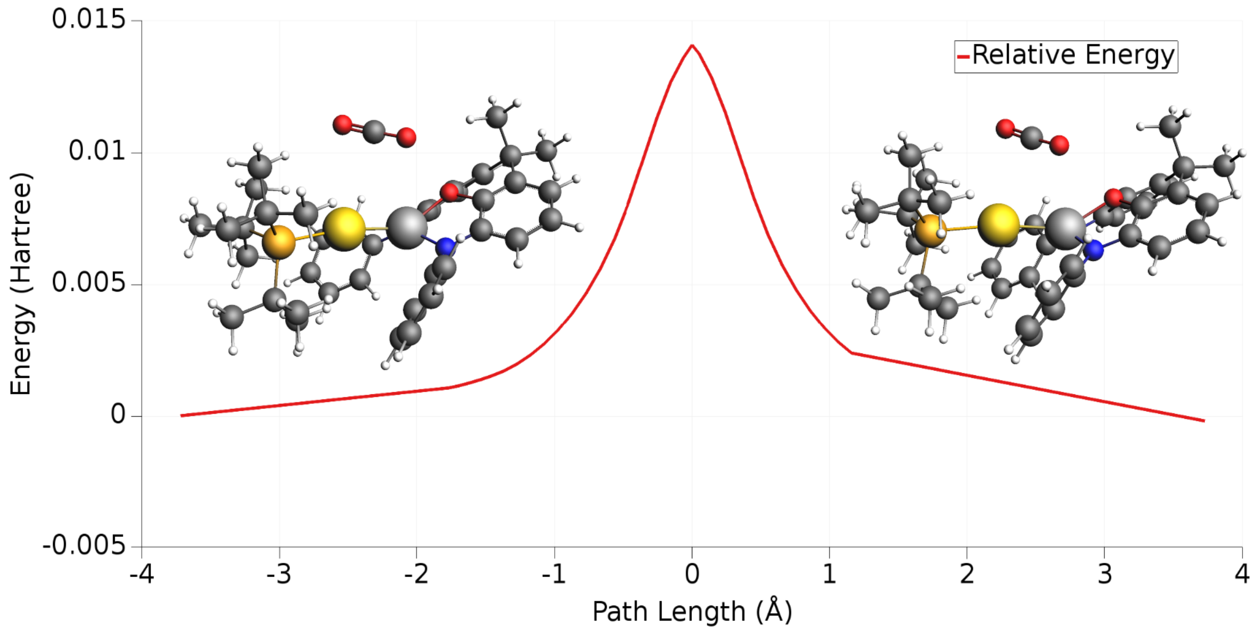

Figure S11. One-dimensional IRC minimum energy reaction path for the first step of the reaction of complex **I** with CO_2. Insets: IRC-optimized geometry for the two minima adjacent to TSI.

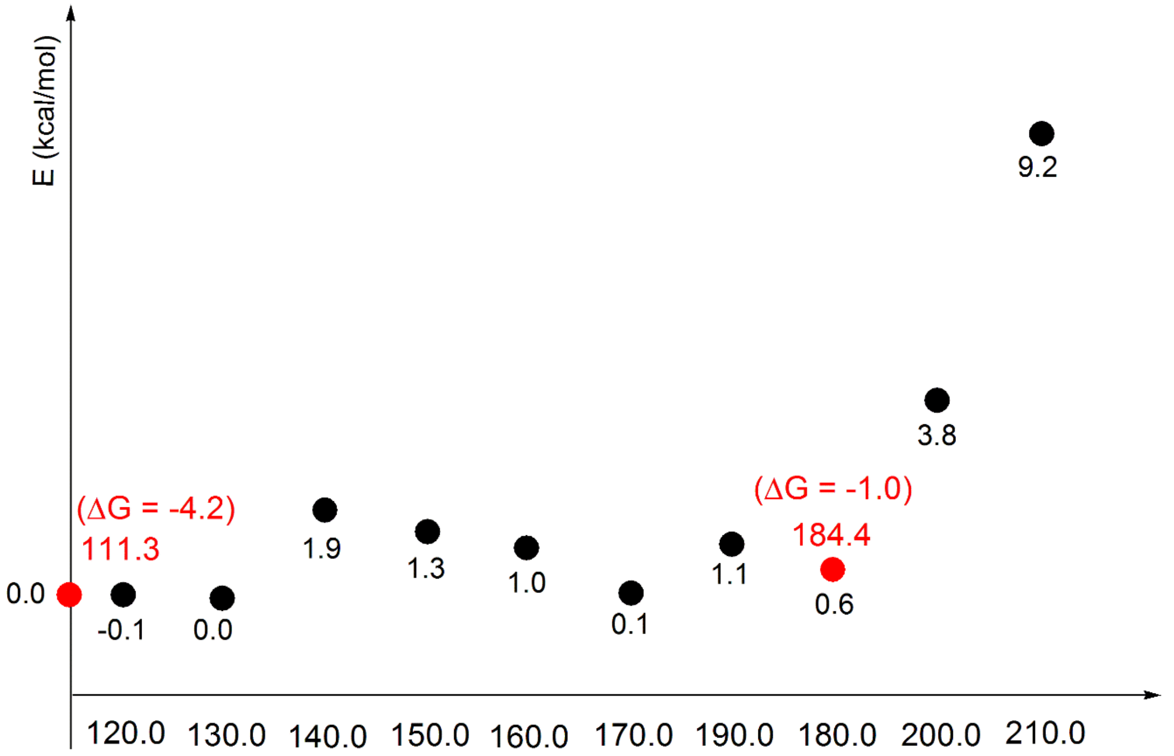

Figure S12. One-dimensional potential energy surface (PES) scan of complex **I** as a function of the Au-Al-O angle (in degrees). Red dots represent the INT stationary point of Figure 2 in the text (with Au-Al-O = 111.3 º) (taken as zero reference energy) and the additionally calculated INT' stationary point (with Au-Al-O = 184.4º). ΔG values refer to the energy of the separated reactants taken as zero.

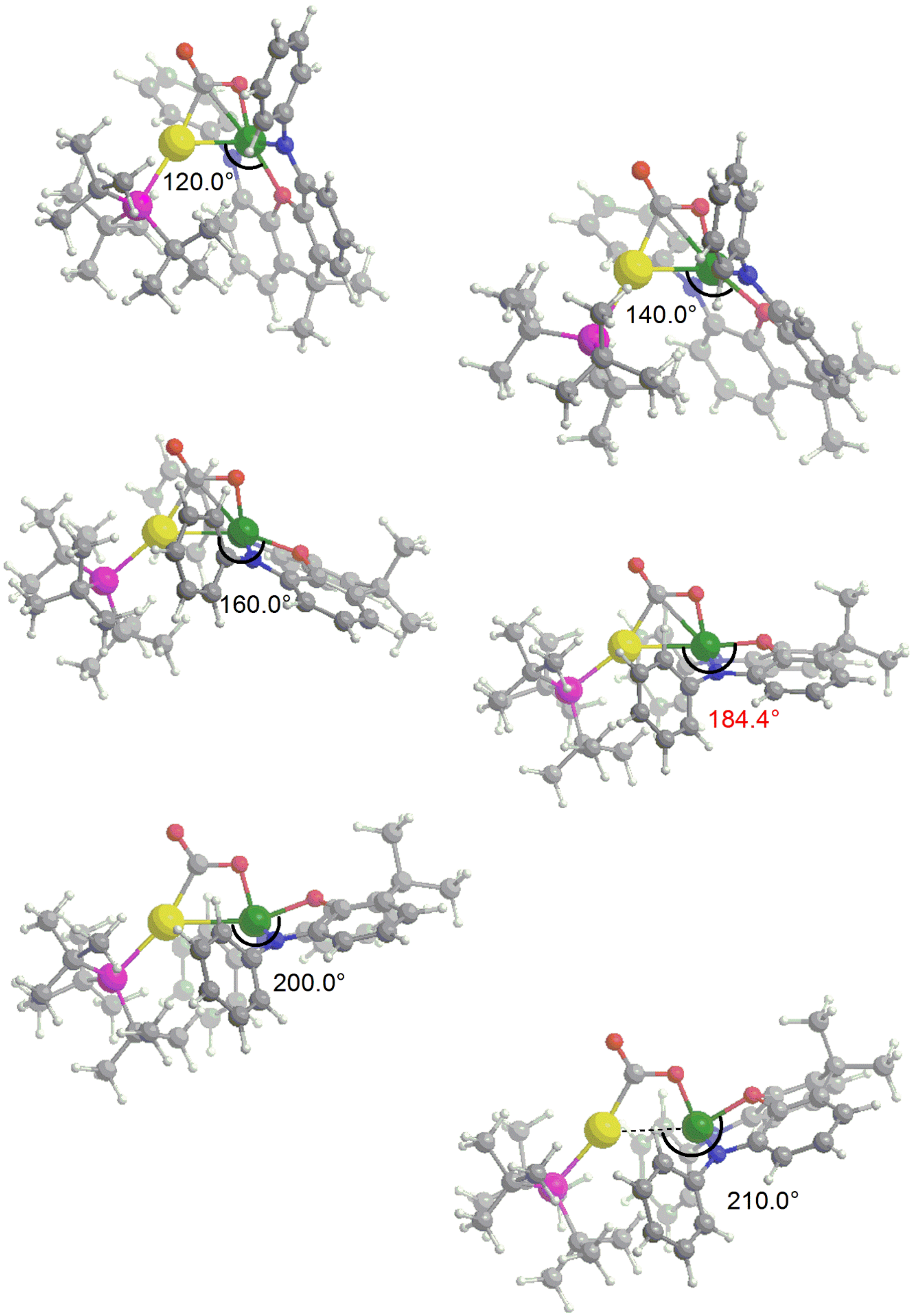

Figure S13. Selected structures (Au-Al-O = 120, 140, 160, 184.4, 200 and 210º angles) taken from the one-dimensional potential energy surface (PES) scan for complex **I** (**Figure S12**).

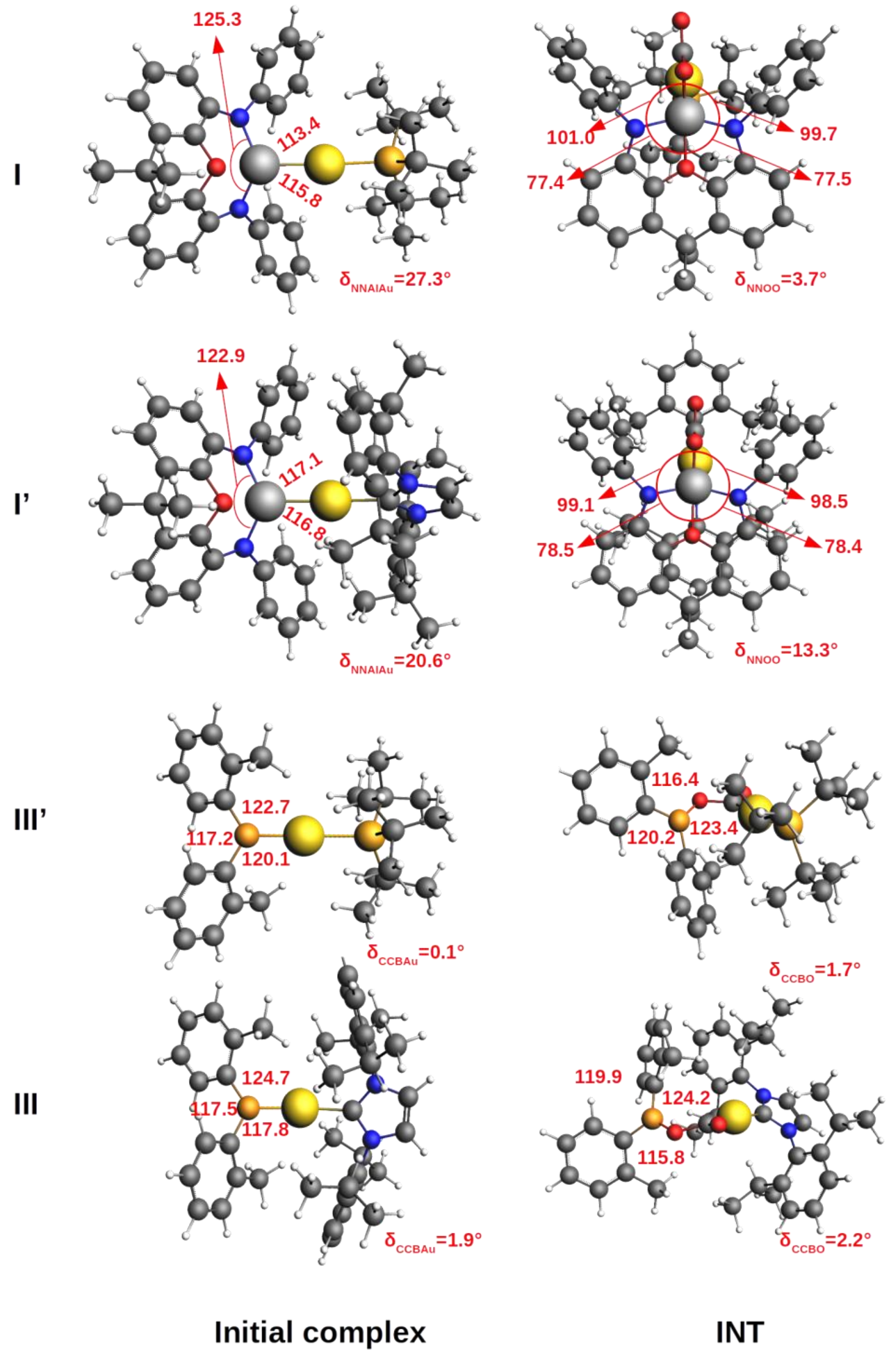

Figure S14. Analysis of the geometry around B and Al for initial complexes **I**, **I'**, **III'**, **III** and the corresponding INT structures. The main bond angles are reported together with the most significant dihedral angles (in degrees).

	[^{t}Bu$_3$PAu Al(NON’)]	[^{t}Bu$_3$PAu B(*o*-tol)]	[IPrAu Al(NON’)]	[IPrAu B(*o*-tol)]
**$\Delta E^{\#}$**	9.05	11.42	8.65	11.70
**ΔE_{INT}^{RC}**	-5.10	-5.19	-6.28	-5.29
**ΔE_{INT}^{TS}**	-17.97	-19.60	-10.52	-22.69
**$\Delta\Delta E_{INT}$**	-12.87	-14.41	-4.24	-17.40
**ΔE_{DIST}^{CO2}**	20.34	20.99	6.98	23.43
**$\Delta E_{DIST}^{complex}$**	1.58	4.84	5.91	5.67
**$\Delta\Delta E_{DIST}$**	21.92	25.83	12.89	29.10

Table S9. Results of the Activation Strain Model (ASM) analysis of the [CO_2]-[LAuX(NON’)] (L=^{t}Bu$_3$P, IPr ; X=Al,B) interaction for the electronic energy activation barrier involving **I/I’** and **III/III’** respectively. All energies are expressed in kcal/mol.

	[^{t}Bu$_3$PAu Al(NON’)]	[^{t}Bu$_3$PAu B(*o*-tol)]	[IPrAu Al(NON’)]	[IPrAu B(*o*-tol)]
**$\Delta E^{\#}$**	-21.05	-30.62	-18.15	-29.49
**ΔE_{INT}^{TS}**	-17.97	-19.60	-10.52	-22.69
**ΔE_{INT}^{INT}**	-107.02	-184.78	-96.50	-194.29
**$\Delta\Delta E_{INT}$**	-89.05	-165.18	-85.98	-171.60
**ΔE_{DIST}^{CO2}**	44.30	74.21	52.91	73.17
**$\Delta E_{DIST}^{complex}$**	23.70	60.35	14.92	68.94
**$\Delta\Delta E_{DIST}$**	68.00	134.56	67.83	142.11

Table S10. Results of the Activation Strain Model (ASM) analysis of the [CO_2]-[LAuX(NON’)] (L=^{t}Bu$_3$P, IPr ; X=Al,B) interaction for the INT stabilization involving **I/I’** and **III/III’** respectively. All energies are expressed in kcal/mol.

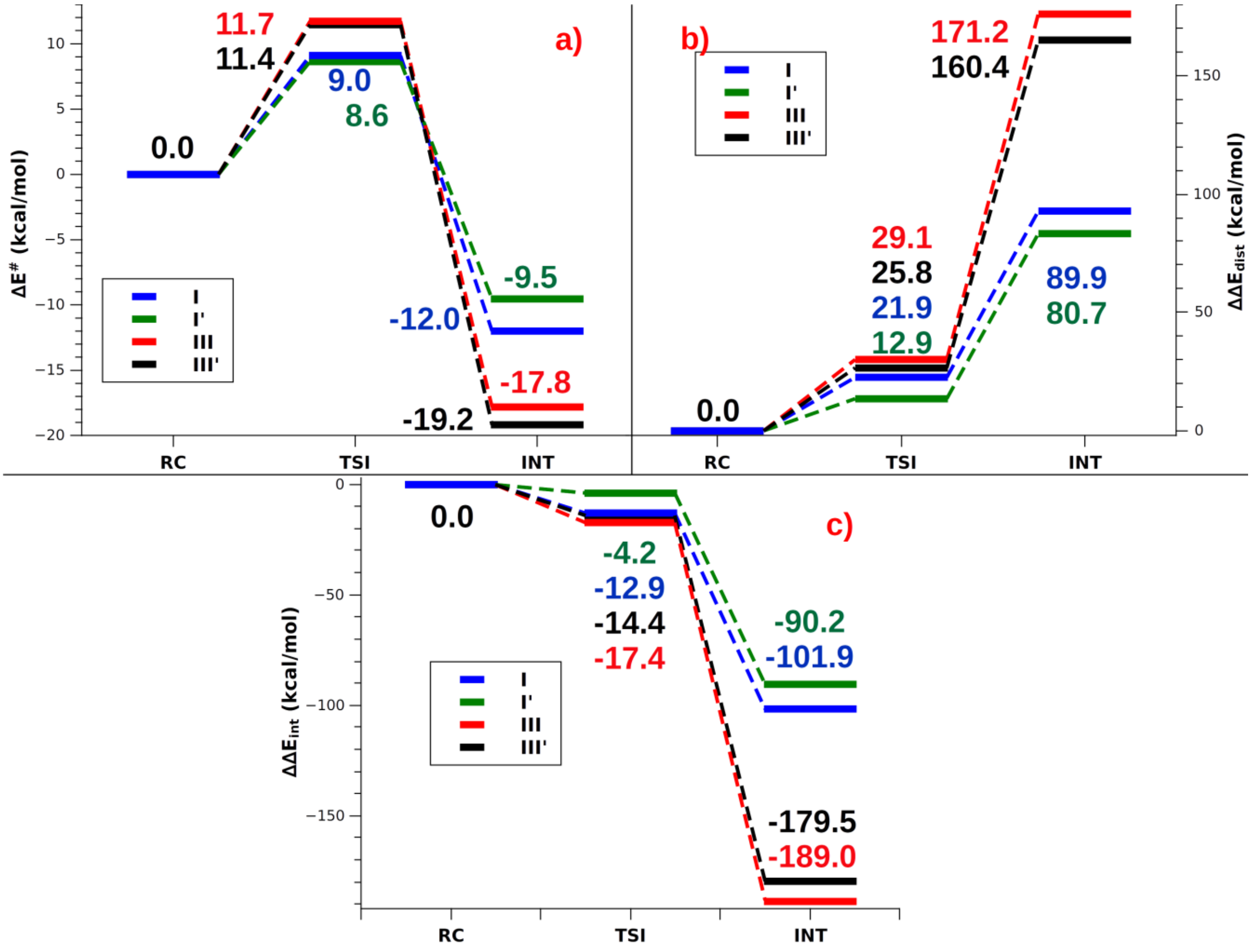

Figure S15. *a)* ASM diagrams for the electronic energy variation (*ΔE*) along the reaction path connecting RC, TSI and INT structures for complexes **I**, **I'**, **III**, **III'**. *b)* ASM diagrams for the variation of the distortion energy penalty ($\Delta\Delta E_{dist}$) along the reaction path connecting RC, TSI and INT structures for complexes **I**, **I'**, **III**, **III'**. *c)* ASM diagrams for the variation of the interaction energy stabilization ($\Delta\Delta E_{int}$) along the reaction path connecting RC, TSI and INT structures for complexes **I**, **I'**, **III**, **III'**. See the Methodology section for insights into the ASM scheme used here.

The ASM analysis reveal that **I** and **III'** display comparably low electronic energy activation barriers associated to TSI ($\Delta E^{\#}$ is 9.0 and 11.4 kcal/mol for **I** and **III'**, respectively), and, in agreement with the free energy profiles reported in Figure 2 in the text, a more stable intermediate is formed for **III'** (-19.2 vs -12.0 kcal/mol, respectively). The larger stability of the intermediate for **III'** originates from a high distortion penalty (160.4 kcal/mol) which is more efficiently counterbalanced by the stabilizing interactions between **III'** and CO_2 (-179.5 kcal/mol) with respect to **I**.

	[^{t}Bu$_3$PAu Al(NON')]	[^{t}Bu$_3$PAu B(*o*-tol)]	[IPrAu Al(NON')]	[IPrAu B(*o*-tol)]
**ΔE_{Pauli}**	102.70	105.29	68.93	119.27
**ΔE_{Elst}**	-60.31	-59.39	-44.74	-66.51
**ΔE_{Steric}**	42.39	45.90	24.19	52.77
**ΔE_{oi}**	-55.82	-60.01	-29.45	-69.28
**ΔE_{oi}^1**	-41.16	-42.49	-18.28	-48.49
**ΔE_{oi}^2**	-3.99	-7.74	-5.35	-8.63
**ΔE_{disp}**	-5.47	-5.56	-7.13	-6.44
**ΔE**	-18.90	-19.67	-12.38	-22.95

Table S11. Results of the Energy Decomposition Analysis (EDA) of the [CO_2]-[LAuX(NON')] (L=^{t}Bu$_3$P, IPr; X=Al,B) interaction for the TSI involving complexes **I/I'** and **III/III'** respectively. All energies are expressed in kcal/mol.

	[^{t}Bu$_3$PAu Al(NON')]	[^{t}Bu$_3$PAu B(*o*-tol)]	[IPrAu Al(NON')]	[IPrAu B(*o*-tol)]
**ΔE_{Pauli}**	390.55	665.38	381.00	678.82
**ΔE_{Elst}**	-227.27	-342.43	-222.21	-356.02
**ΔE_{Steric}**	163.28	322.96	158.79	322.80
**ΔE_{oi}**	-262.87	-503.68	-248.33	-513.31
**ΔE_{oi}^1**	-215.84	-389.26	-200.78	-389.06
**$\lvert CT^1 \rvert$**	0.66	0.71	0.64	0.72
**ΔE_{oi}^2**	-10.84	-47.08	-11.19	-50.70
**$\lvert CT^2 \rvert$**	0.07	0.18	0.07	0.18
**ΔE_{disp}**	-5.66	-4.48	-6.65	-5.04
**ΔE**	-105.25	-185.21	-96.19	-195.56

Table S12. Results of the Energy Decomposition Analysis (EDA) of the [CO_2]-[LAuX(NON')] (L=^{t}Bu$_3$P, NHC^{Dipp}; X=Al, B) interaction for the INT involving complexes **I/I'** and **III/III'** respectively. All energies are expressed in kcal/mol.

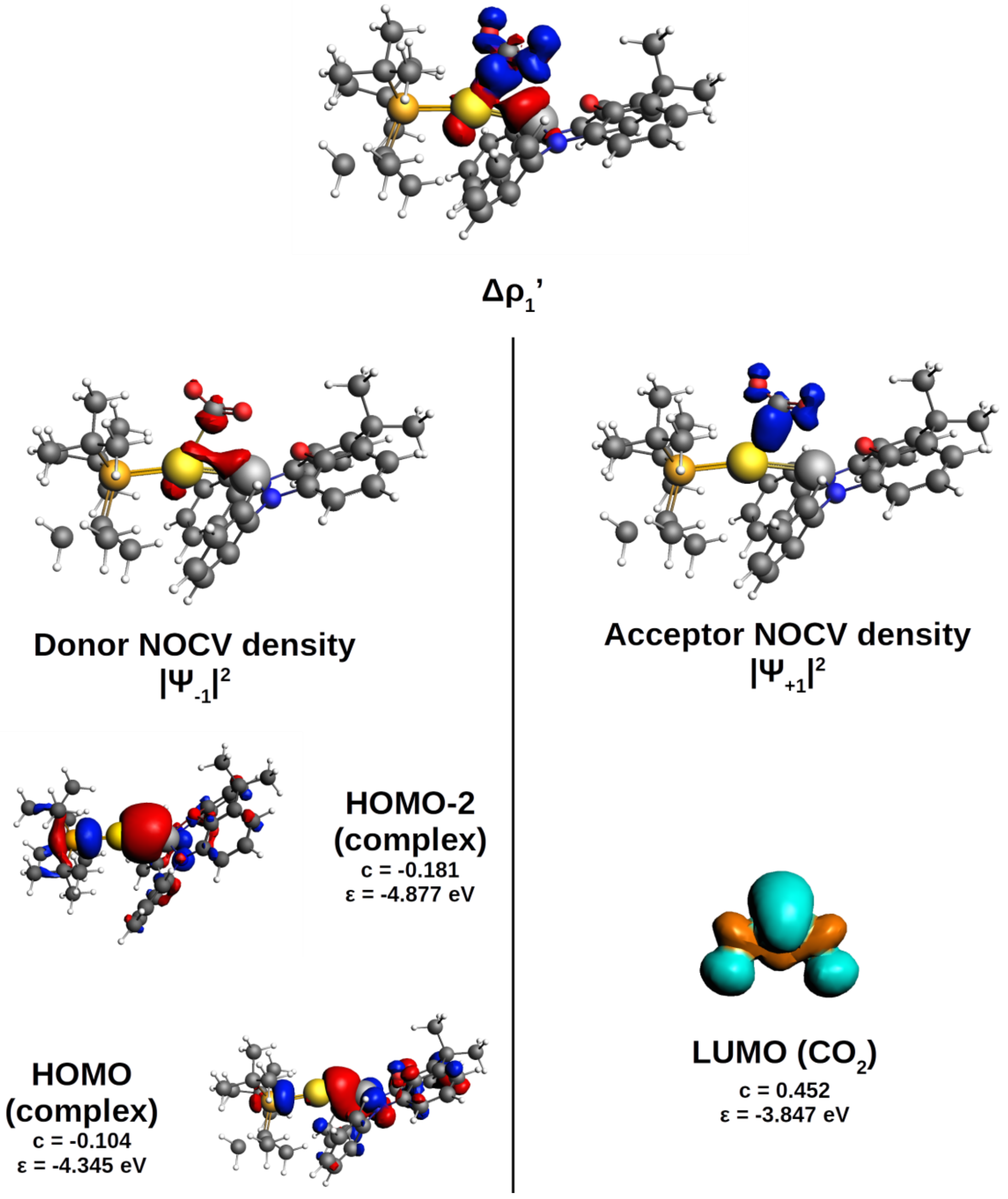

Figure S16. Breakdown of the donor ($|\Psi_1|^2$) and acceptor ($|\Psi_{-1}|^2$) NOCV densities that are associated with the deformation density $\Delta\rho'_1$ in the transition state TSI involving complex **I** into the most important MOs of the fragments frozen at their TSI geometry. The mixing coefficients are given in parentheses.

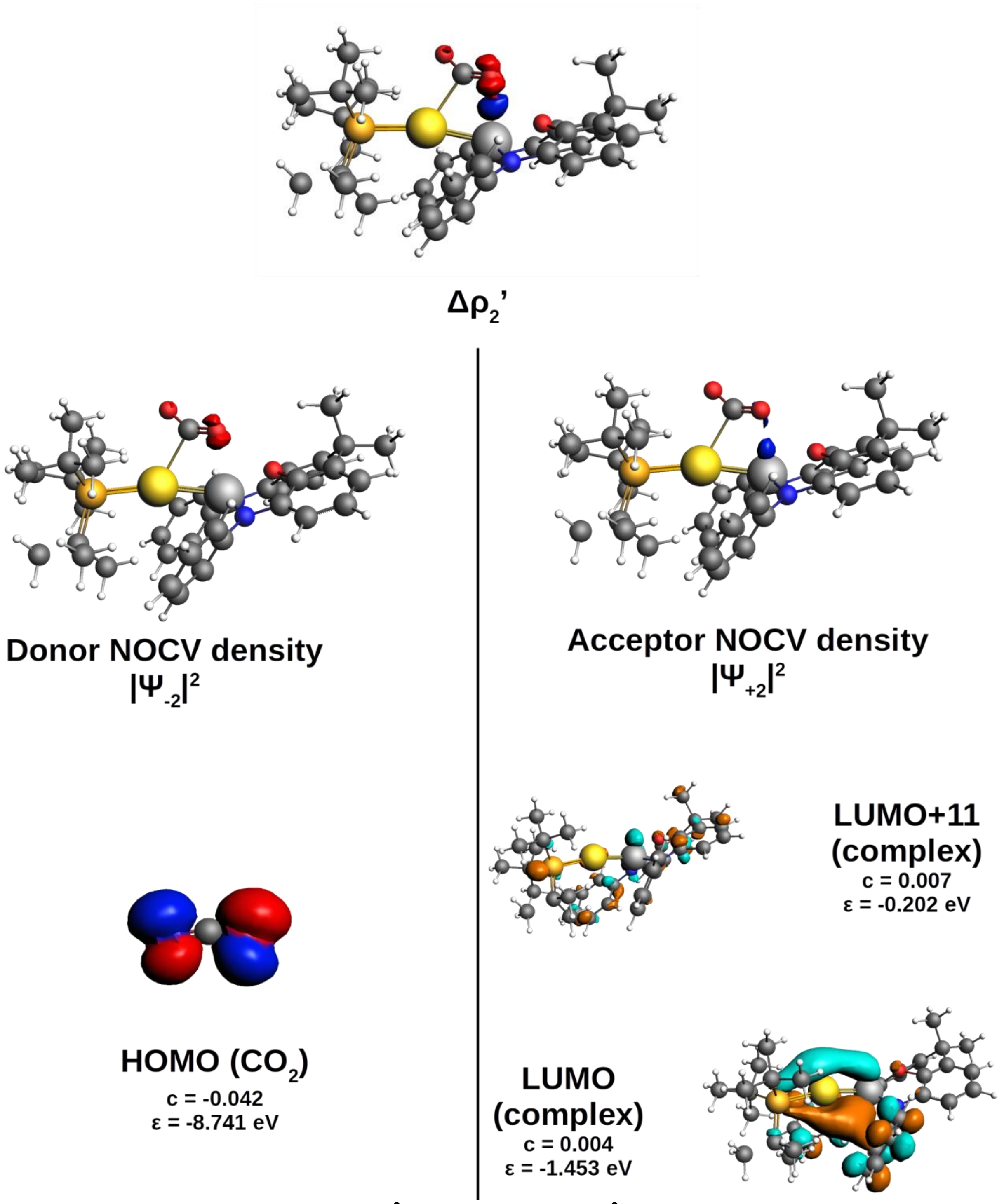

Figure S17. Breakdown of the donor ($|\Psi_2|^2$) and acceptor ($|\Psi_{-2}|^2$) NOCV densities that are associated with the deformation density $\Delta\rho'_2$ in the transition state TSI involving complex **I** into the most important MOs of the fragments frozen at their TSI geometry. The mixing coefficients are given in parentheses.

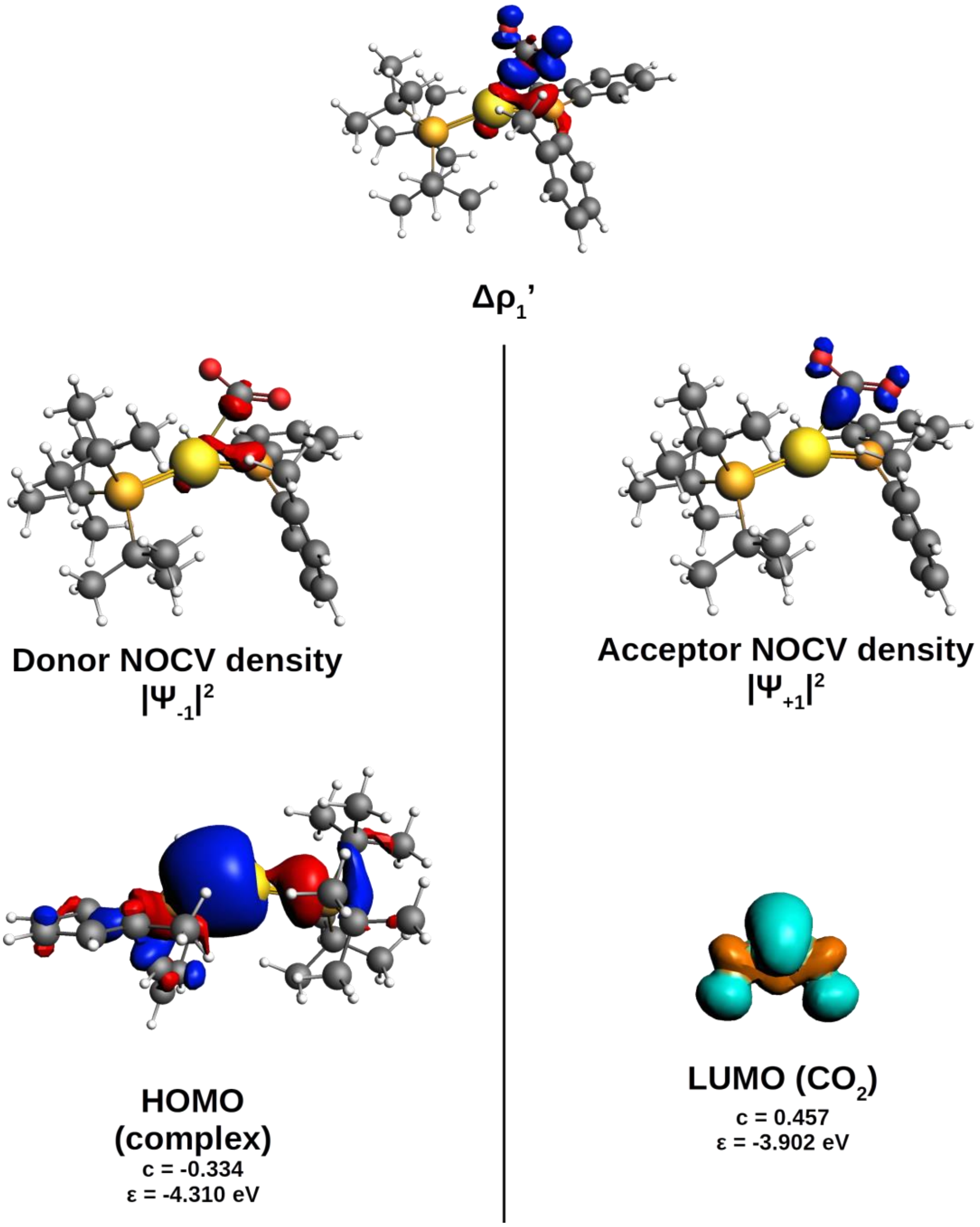

Figure S18. Breakdown of the donor ($|\Psi_1|^2$) and acceptor ($|\Psi_{-1}|^2$) NOCV densities that are associated with the deformation density $\Delta\rho'_1$ in the transition state TSI involving complex **III'** into the most important MOs of the fragments frozen at their TSI geometry. The mixing coefficients are given in parentheses.

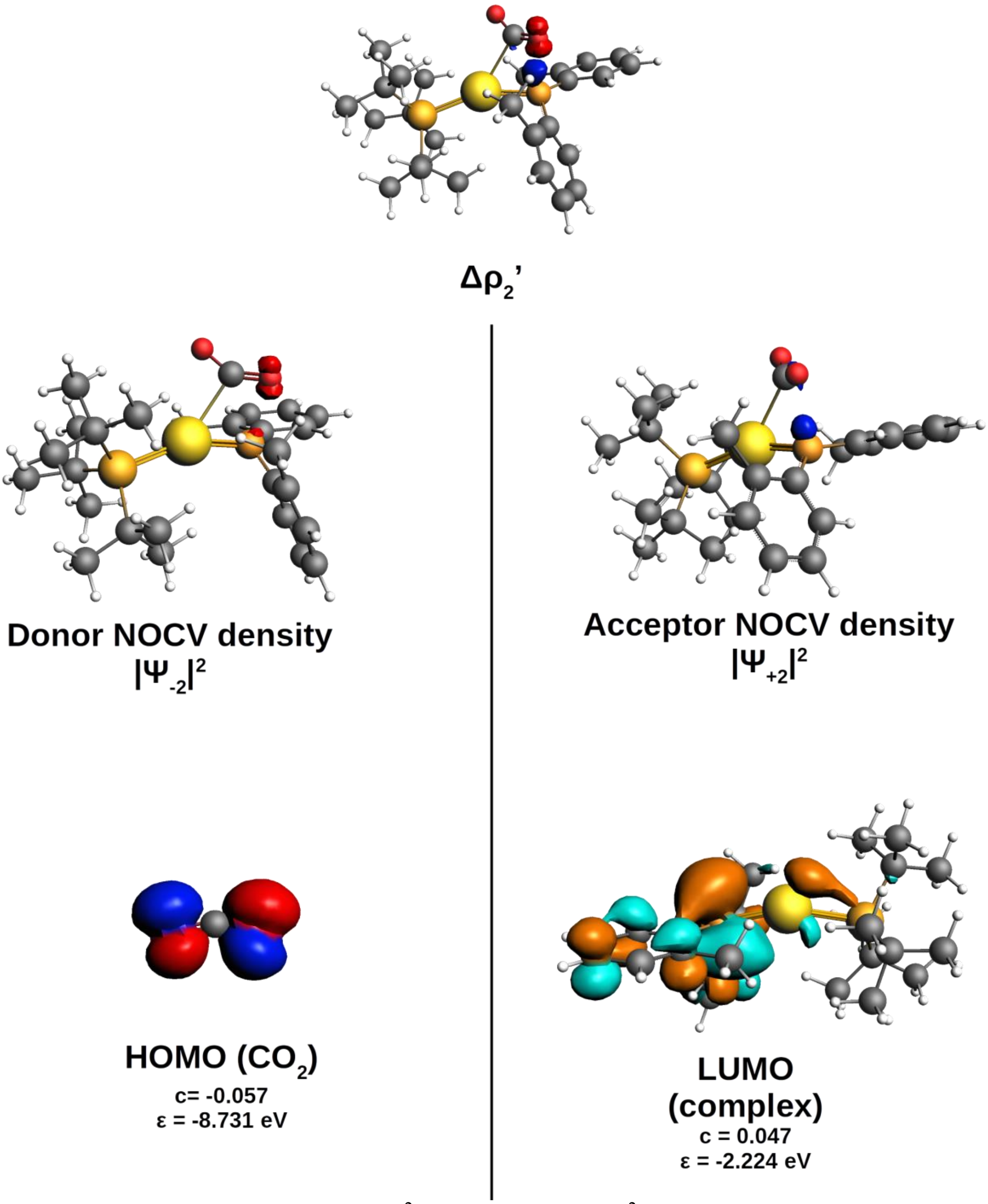

Figure S19. Breakdown of the donor ($|\Psi_2|^2$) and acceptor ($|\Psi_{-2}|^2$) NOCV densities that are associated with the deformation density $\Delta\rho'_2$ in the transition state TSI involving complex **III'** into the most important MOs of the fragments frozen at their TSI geometry. The mixing coefficients are given in parentheses.

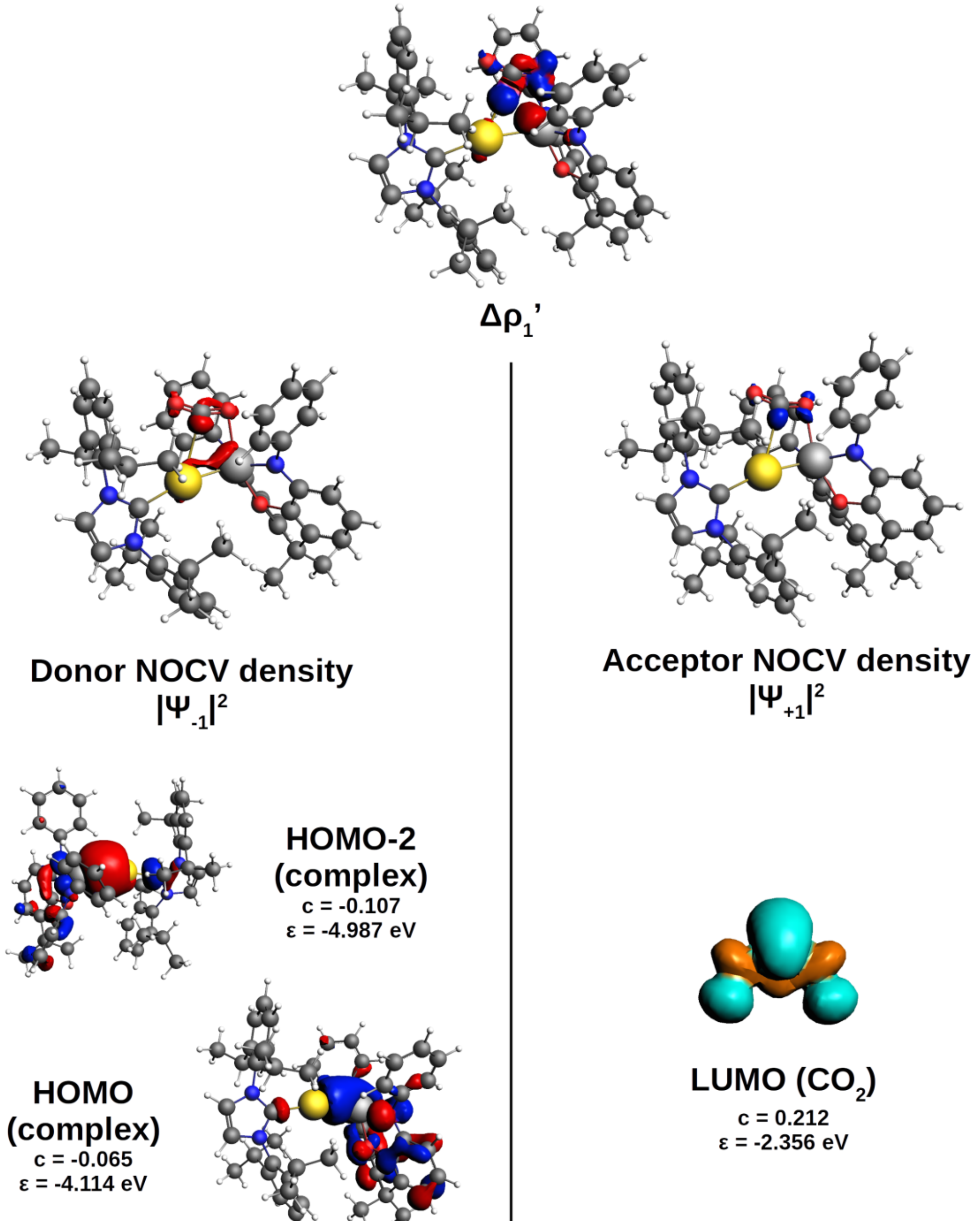

Figure S20. Breakdown of the donor ($|\Psi_1|^2$) and acceptor ($|\Psi_{-1}|^2$) NOCV densities that are associated with the deformation density $\Delta\rho'_1$ in the transition state TS involving complex **I'** into the most important MOs of the fragments frozen at their TS geometry. The mixing coefficients are given in parentheses.

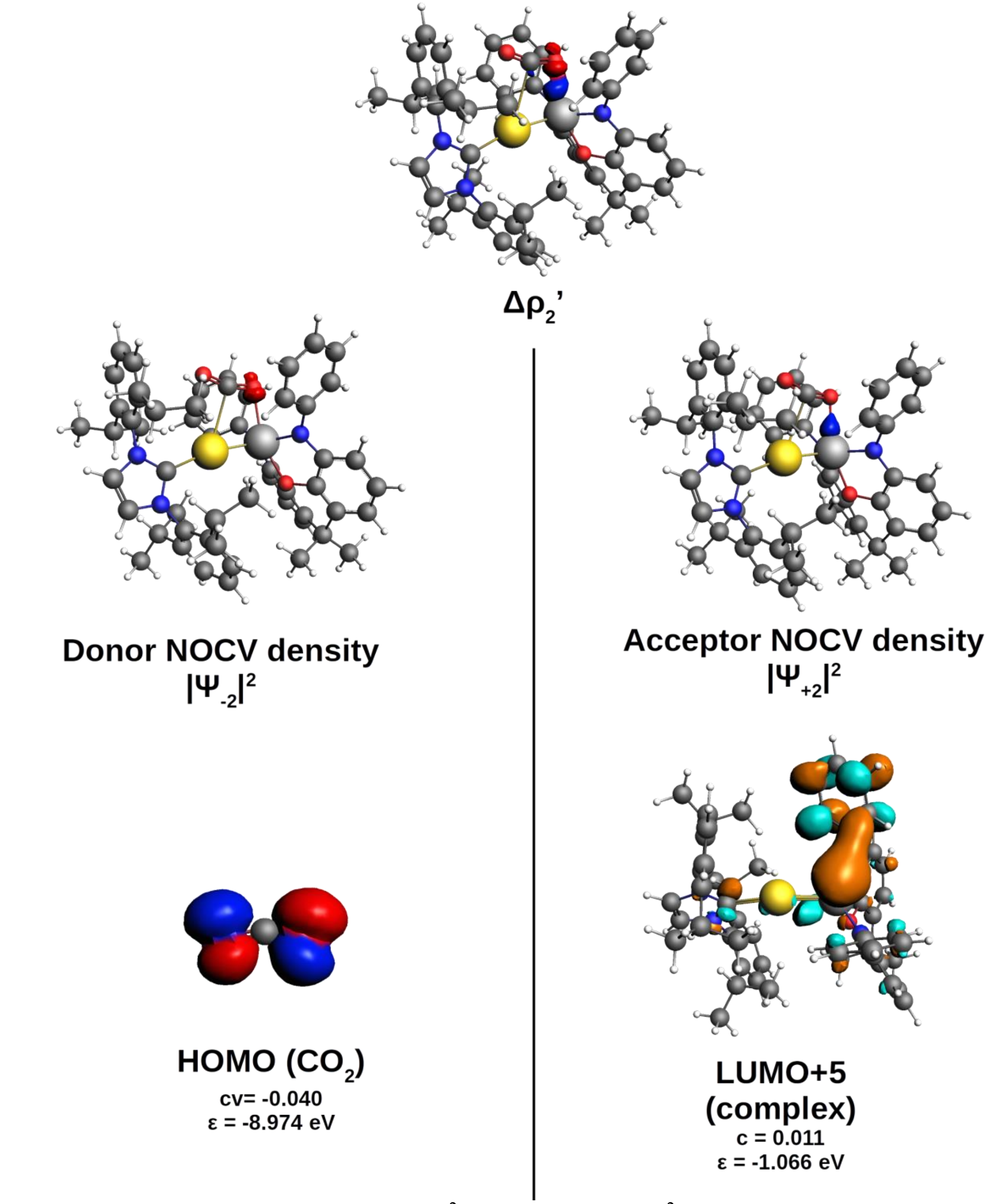

Figure S21. Breakdown of the donor ($|\Psi_2|^2$) and acceptor ($|\Psi_{-2}|^2$) NOCV densities that are associated with the deformation density $\Delta\rho'_2$ in the transition state TSI involving complex **I'** into the most important MOs of the fragments frozen at their TSI geometry. The mixing coefficients are given in parentheses.

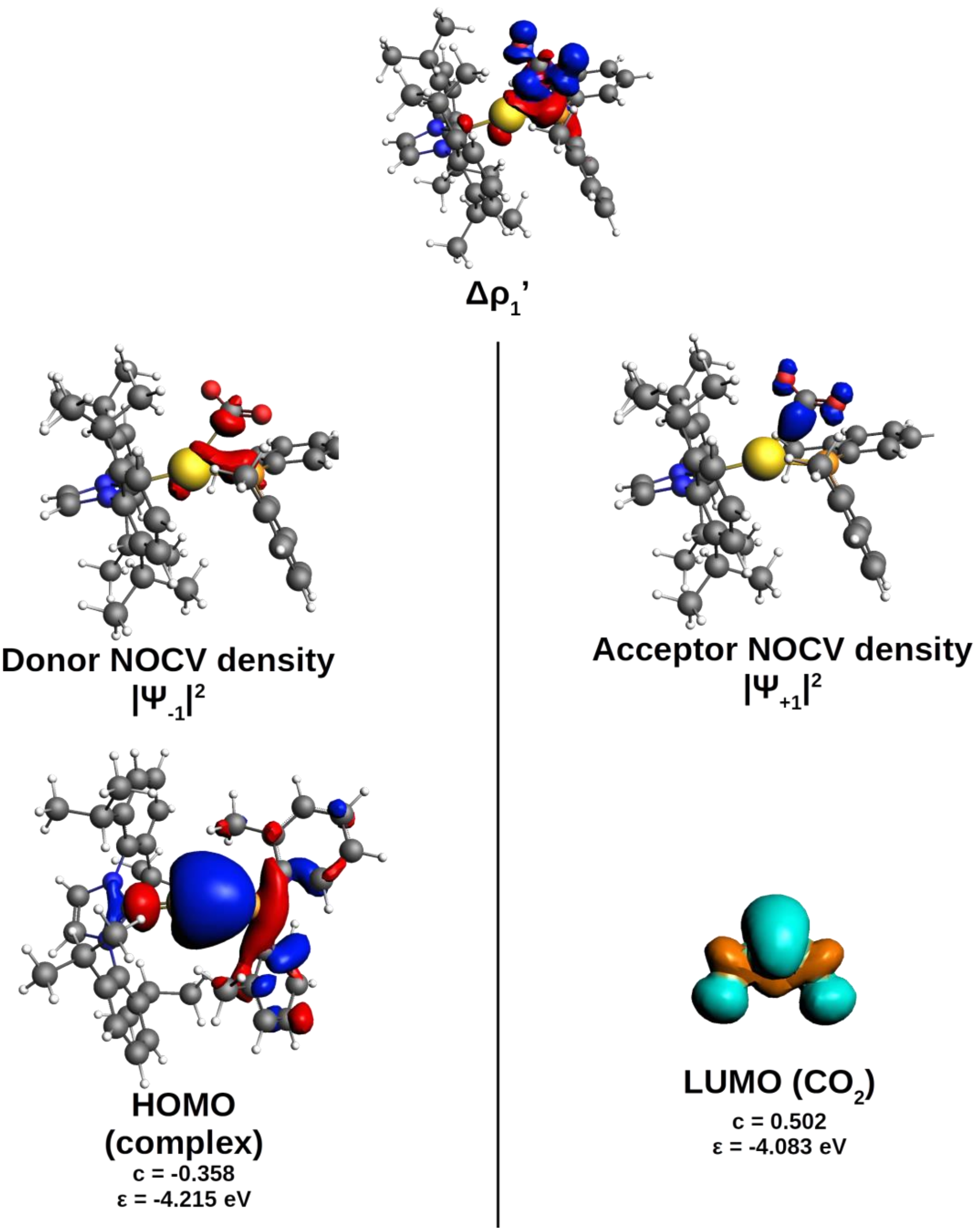

Figure S22. Breakdown of the donor ($|\Psi_1|^2$) and acceptor ($|\Psi_{-1}|^2$) NOCV densities that are associated with the deformation density $\Delta\rho'_1$ in the transition state TSI involving complex **III** into the most important MOs of the fragments frozen at their TSI geometry. The mixing coefficients are given in parentheses.

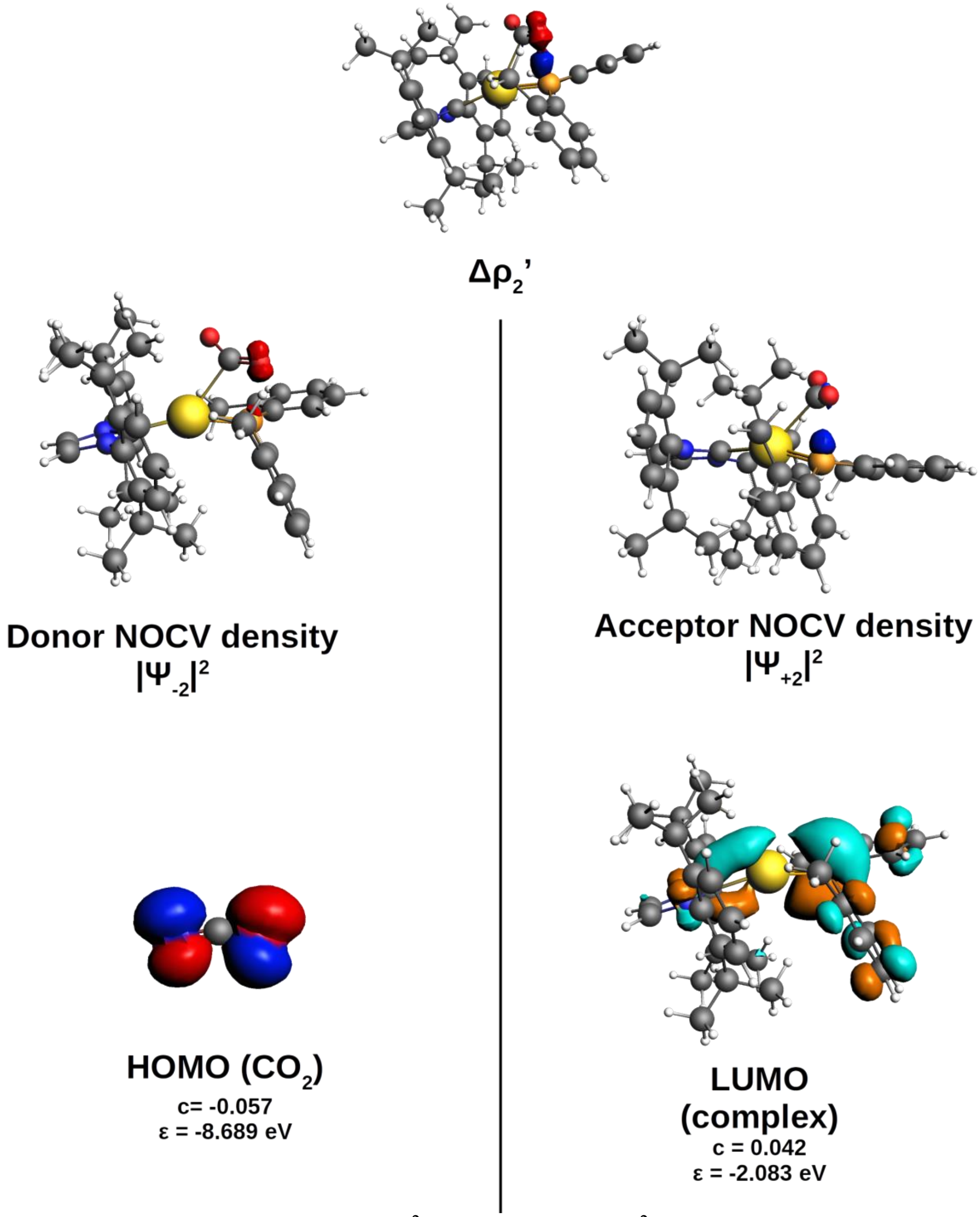

Figure S23. Breakdown of the donor ($|\Psi_2|^2$) and acceptor ($|\Psi_{-2}|^2$) NOCV densities that are associated with the deformation density $\Delta\rho'_2$ in the transition state TS involving complex **III** into the most important MOs of the fragments frozen at their TS geometry. The mixing coefficients are given in parentheses.

	I		I’		III’		III	
	**[Au]**	**[Al]**	**[Au]**	**[Al]**	**[Au]**	**[B]**	**[Au]**	**[B]**
f^-	0.045	0.033	0.042	0.028	0.125	0.054	0.147	0.055
f^+	0.082	0.075	0.032	0.032	0.074	0.114	0.048	0.075
$f^{(2)}$	0.037	0.042	-0.010	0.004	-0.051	0.060	-0.099	0.020

Table S13. Local Fukui minus (f^-), Fukui plus (f^+) function and dual descriptor ($f^{(2)}$) (Hirschfeld partitioning) for the complexes **I**, **I’**, **III**, and **III’**.

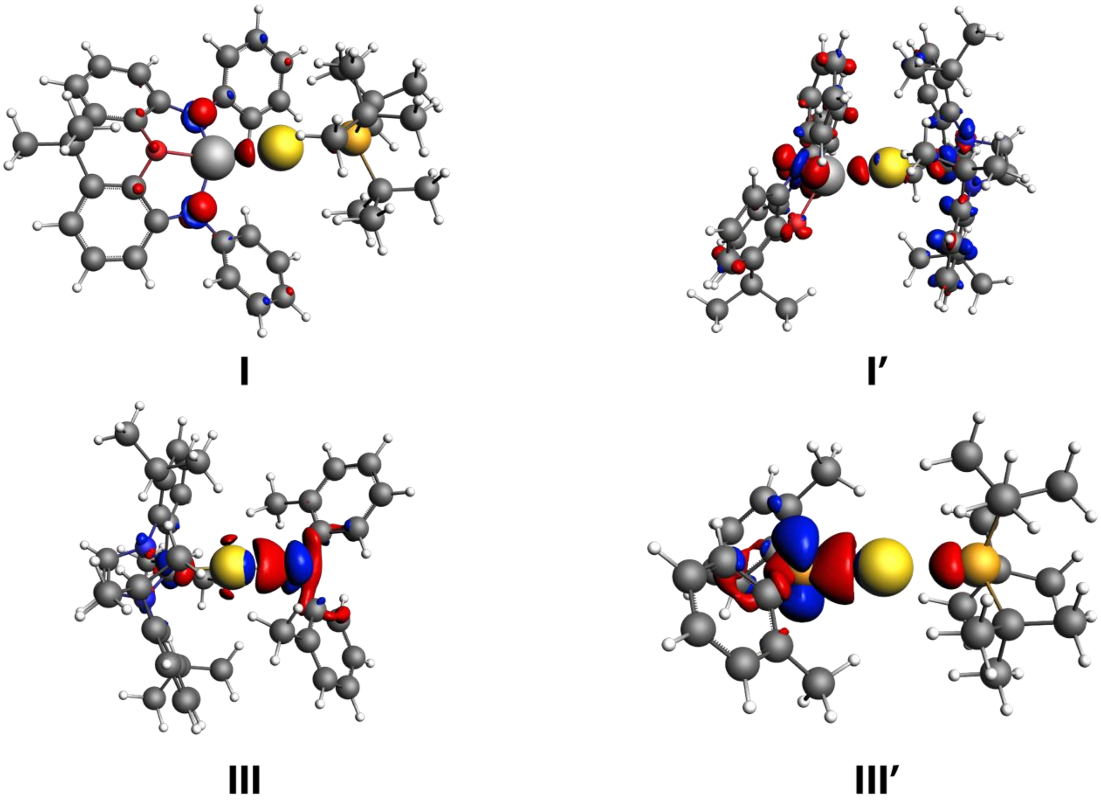

Figure S24. Isosurface plots of the dual descriptor (isovalue 0.003 e/a_0) for the complexes **I**, **I’**, **III**, and **III’**. Electrophilic regions are indicated in blue, nucleophilic regions in red.

	ΔE_{int}	ΔE_{prep}^{CO2}	ΔE_{prep} [Al]/[B]	ΔE_{prep} [Au]	ΔE_{prep}	ΔE
**I (neutral)**	-200.72	94.37	0.09	0.31	94.77	-105.95
**I (charged)**	-253.20	94.37	11.54	3.71	109.62	-143.58
**III' (neutral)**	-222.77	114.41	9.59	0.25	124.25	-98.52
**III' (charged)**	-277.33	114.41	12.14	3.44	129.99	-147.34
**I' (neutral)**	-216.68	95.24	0.95	0.69	96.88	-119.80
**I' (charged)**	-267.20	95.24	12.29	2.27	109.80	-157.40
**III (neutral)**	-234.24	115.73	10.03	0.76	126.52	-107.72
**III (charged)**	-286.65	115.73	11.99	2.49	130.21	-156.44

Table S14. Interaction energy (ΔE_{int}) and preparation energy of the [LAu] ($\Delta E_{prep}^{[Au]}$), boryl/aluminyl ($\Delta E_{prep}^{[Al]/[B]}$) and CO_2 (ΔE_{prep}^{CO2}) fragments concerning the formation of PCs **I**, **I'**, **III**, **III'** using both charges singlet $[LAu]^+$ and $[X]^-$ and neutral doublet [LAu]· and [X]· fragments. The overall preparation (ΔE_{prep}) and formation (ΔE) energies are reported as well. All energies are expressed in kcal/mol.

Use of either charged or neutral fragments for the formation/dissociation of the product complex shows no remarkable effect when changing the ligand/anion and the effect is systematic overall for all the complexes. In all cases, the formation energy associated to the PC is higher when using the charged fragments, thus pointing out that the most likely fragmentation for the formation/dissociation of the PC is in any case the one featuring neutral doublet gold and boryl/aluminyl fragments.

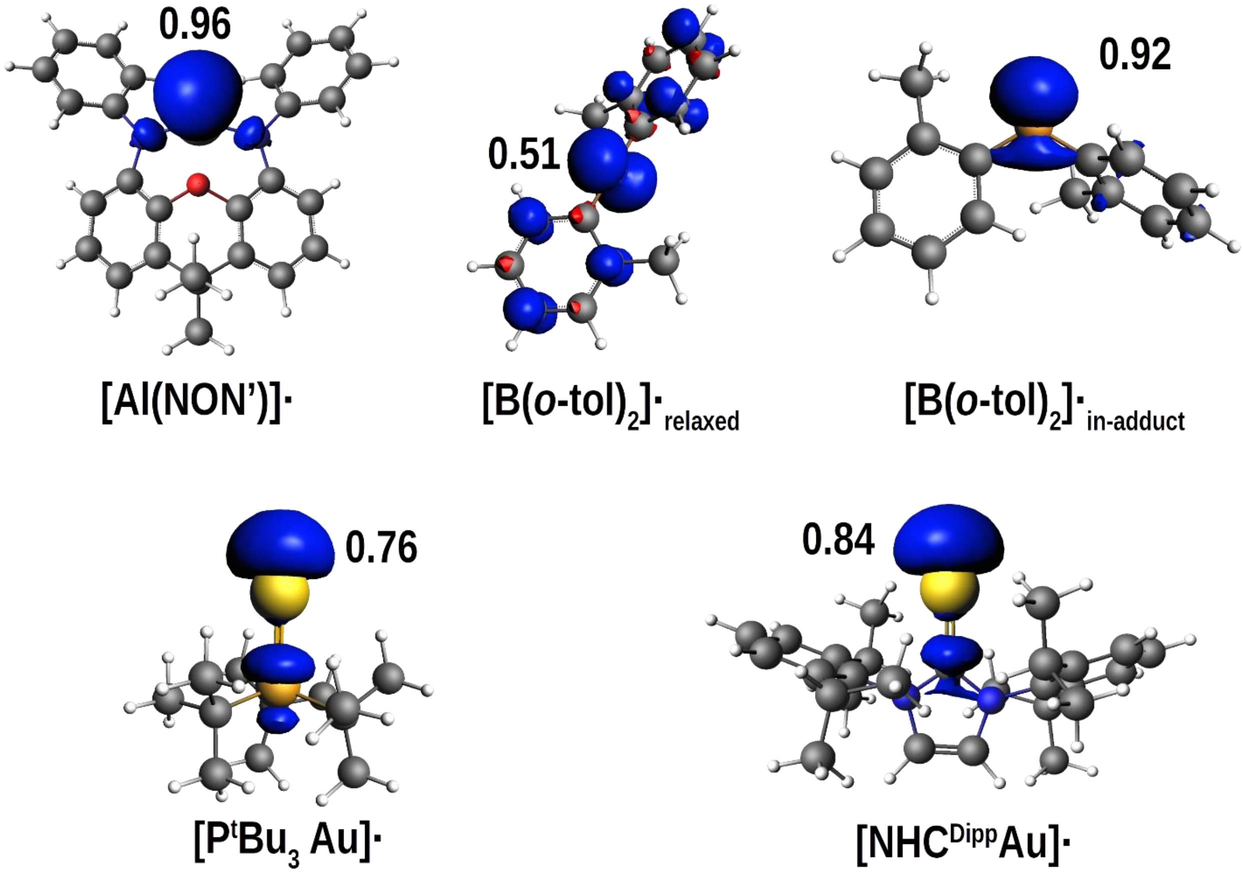

Figure S25. Spin densities of the [Al(NON')]· (top, left), relaxed B[(o-tol)$_2$]· (top, centre), in-adduct B[(o-tol)$_2$]· (top, right), [^{t}Bu$_3$PAu]· (bottom, left) and [IPrAu]· (bottom, right) radicals. The geometries have been frozen at the in-adduct geometry they have in **I** and **I'** respectively. The spin density on Au is reported for all radicals.

Aluminyl vs. boryl – [IPrAu]: anion effect on the Au-X bond and reaction mechanism

Application of the CD-NOCV approach for the analysis of the Au-X bond in complexes **I'** and **III** reveals that, upon substitution of the phosphine ancillary ligand of the gold moiety with a N-heterocyclic carbene, the differences between the Au-Al and Au-B bonds discussed in the main text do not change. Likewise, the Au-X bond in **I'** and **III** mainly consists of two opposite Au-to-X and X-to-Au charge transfers assisted by a smaller dative back-donation component. The same anion effect can be seen, as demonstrated by the numerical results of the CD-NOCV analysis shown in Table S15 and Table S5.

	$\Delta E_{oi}^{1\alpha}$	$CT^{1\alpha}$	$\Delta E_{oi}^{1\beta}$	$CT^{1\beta}$	ΔE_{oi}^{2}	CT^{2}
**I'**	-33.6	-0.307	-24.2	0.275	-4.3	-0.046
**III**	-61.7	-0.325	-23.9	0.277	-7.8	-0.068

Table S15. Orbital interaction energies (ΔE_{oi}^{k}) (in kcal/mol) and charge transfer (CT^{k}) (in electrons, e) associated with the first two NOCV deformation densities for the interaction between neutral doublet [$^{t}Bu_3PAu$]· and [X]· fragments (X=B(*o*-tol)$_2$, Al(NON')) for complexes **I'** and **III**.

Similar to what has been discussed in the main text, the Au-B bond in **III** is slightly more polarized with respect to the Au-Al bond in **I'**. Whereas the extent of the $\Delta\rho_{1\beta}$' bond component, associated to the X-to-Au charge flux, is practically equal in the two complexes (0.275 vs. 0.277 e for **I'** and **III**, respectively, in terms of CT), the Au-to-X charge transfer ($\Delta\rho_{1\alpha}$') is larger for **III** (the associated energies and CTs are -33.6 kcal/mol and -0.307 e for **I'** and -61.7 kcal/mol and -0.325 e for **III**). A slightly reduced electron-sharing character and an increased Au(δ+) -B(δ-) character is analogously observed in **III,** consistently with the increased molecular electronegativity (2.51 vs 2.94 eV for the aluminyl and boryl respectively, Table S6). Moreover, similarly to **III'**, the Au-to-B π back-donation in **III** is clearly enhanced with respect to the backdonation towards Al, as demonstrated by the CT^2 values (-0.046 and -0.068 e for **I'** and **III**, respectively) and the orbital interaction energy (-4.3 and -7.8 kcal/mol for **I'** and **III**, respectively) associated to the $\Delta\rho_{2}$' component.

The reaction profiles for complexes **I'** and **III**, also reported in Figure 2 in the main text (green and blue lines, see Figure S22 for a schematic representation of the stationary point structures along the path and Figures S23-S24 for the optimized structures) are qualitatively very similar to their gold-phosphine counterpart discussed in the main text. The activation free energy barrier for the nucleophilic attack to the CO_2 carbon atom is higher for boryl complex than aluminyl ($\Delta G^{\#}$ =15.7 kcal/mol for **III**, $\Delta G^{\#}$ =9.6 kcal/mol for **I'**), although in terms of electronic energies the two barriers are very similar and also similar to their gold-phosphine counterparts ($\Delta E^{\#}$ =11.7 kcal/mol for **III**,

$\Delta E^{\#}$ =8.6 kcal/mol for **I’,** Table S9), thus suggesting the same anion effect discussed for **I** and **III’**. Analogously, for **III** a more stable INT (-7.2 kcal/mol) is formed, possessing a structure with a substantially broken Au-B, whereas a less stable four-membered cyclic INT (-1.9 kcal/mol) is formed in the case of **I’**.

Application of the CD-NOCV and ETS-NOCV approaches to TSI and INT (see Tables S11-S12 and Figures S16-S19) shows that, consistently, the interaction between **I’**/**III** and CO_2 results from the population of the LUMO of CO_2 via donation from the electron-rich Au-X bond and from the CO_2-to-X donation via population of the valence np_z orbital of the Group 13 element, which is favored for boryl (the associated $\Delta E_{oi}{}^2$ and CT^2 values are -11.2 kcal/mol and 0.07 e for **I’** and -50.70 kcal/mol and 0.18 e for **III’**, see Table S12).

In the second step, the rearrangement of the INT complex through the Au-Al/Au-B bond cleavage and CO_2 oxygen atom bond to Al/B via TSII ($\Delta G^{\#}$ is 9.1 and 5.4 kcal/mol for **III** and **I’**, respectively) yields the insertion products **IV** and **II’**. Analogously to **I** and **III’**, a less stable product complex PC is formed in the case of **III** (-5.9 vs. -16.1 kcal/mol for complex **III** and **I’**, respectively). Similarly, the different stability can be explained on the basis of the radical-like reactivity discussed in the main text using the scheme reported in Figure 5, where, despite the B-O interaction being favoured, the overall formation of the product is favoured for the gold-aluminyl complex **I’** (ΔE values are -119.8 and -107.7 kcal/mol for **I’** and **III**, respectively, see Table S14).

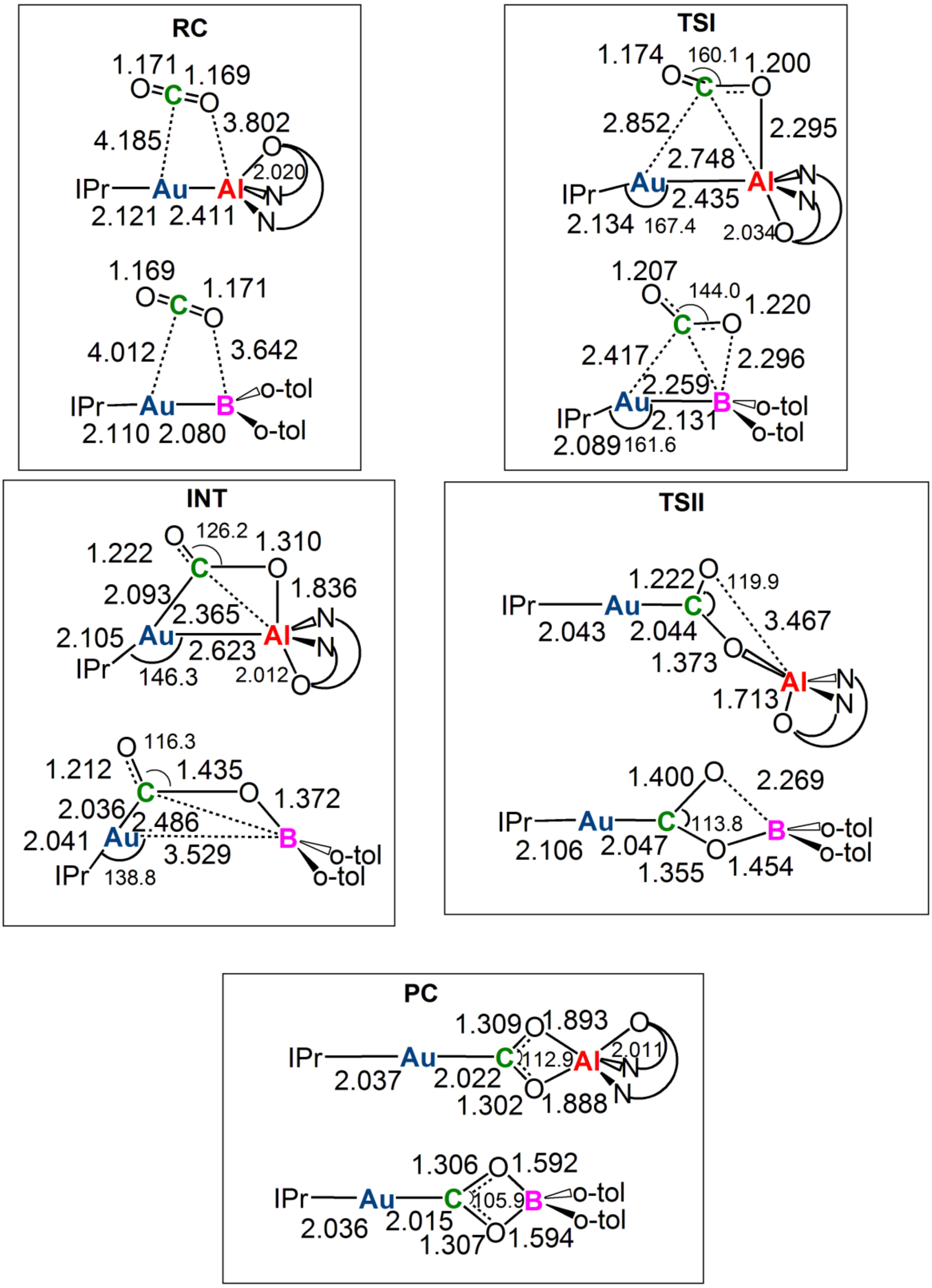

Figure S26. Sketched RC, TSI, INT, TSII and PC structures for the [IPrAuAl(NON')] complex **I'** and the [IPrAuB(*o*-tol)$_2$] complex **III**. Selected interatomic distances (in Å) and bond angles (degrees) are given

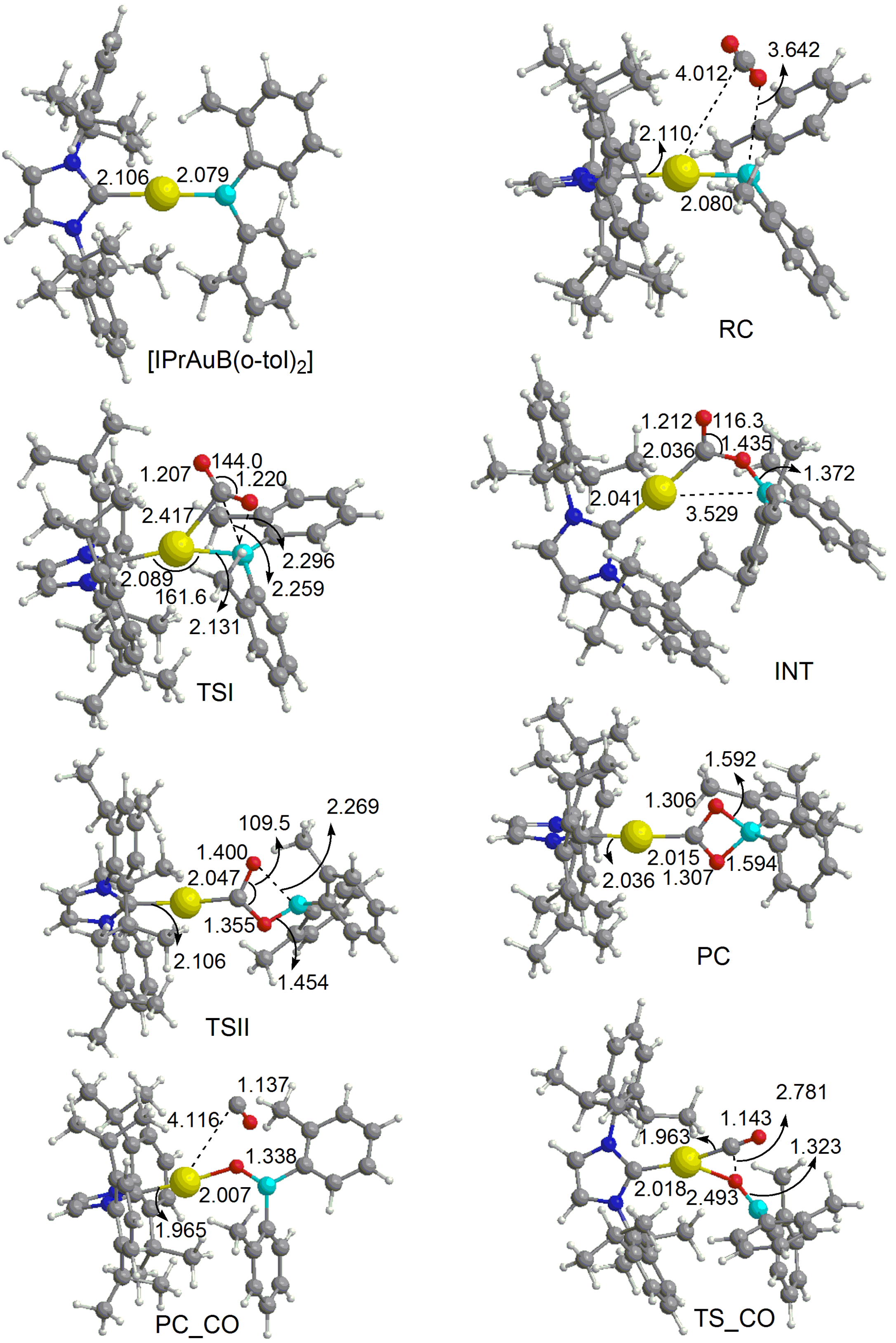

Figure S27. Optimized structures of [IPrAuB(o-tol)$_2$] (complex III), RC, TSI, INT, TSII, PC, TS_CO and PC_CO complexes. Main geometrical parameters are reported (bond in Å, angles in degree).

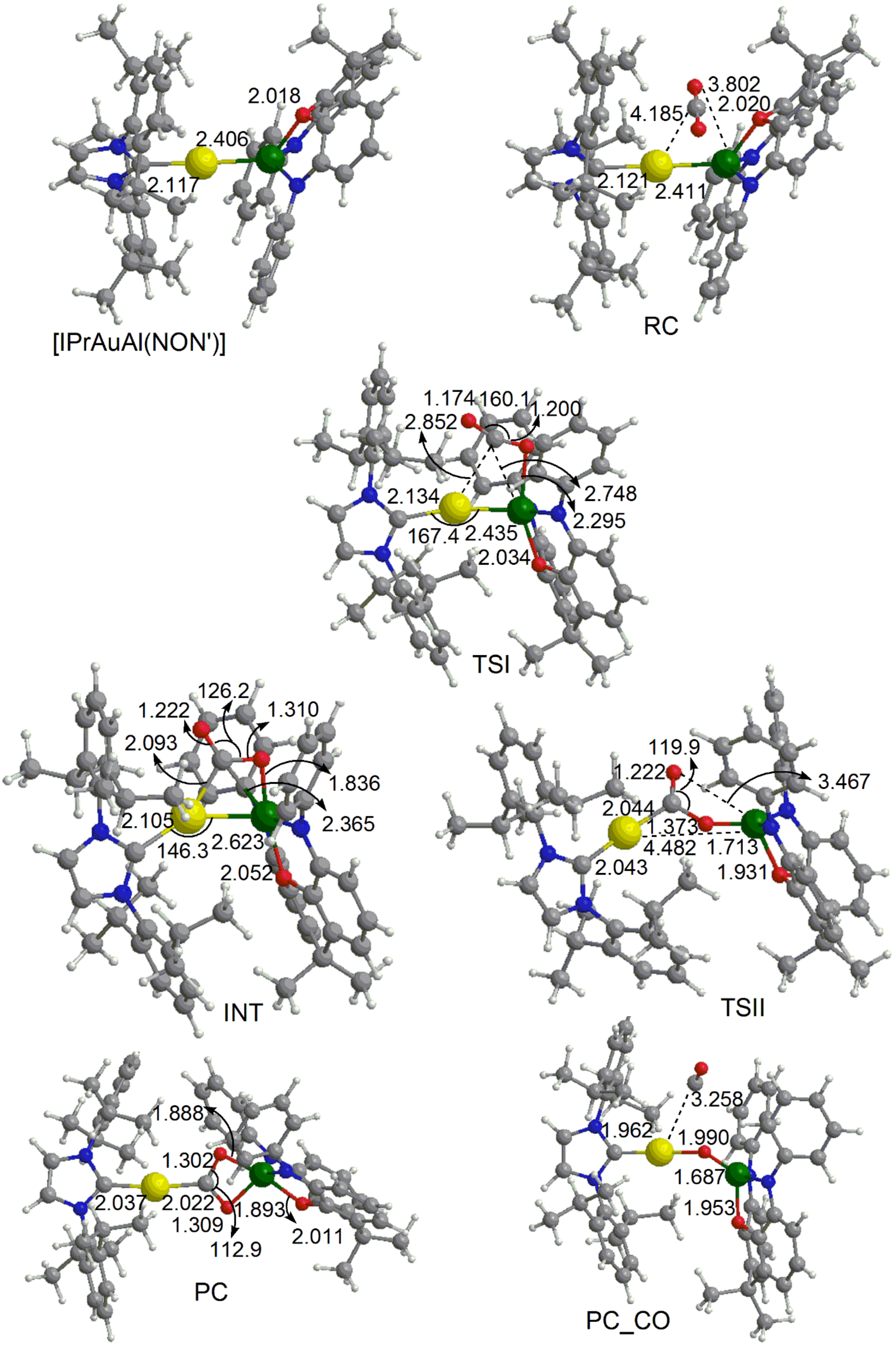

Figure S28. Optimized structures of [IPrAuAl(NON')] (complex I'), RC, TSI, INT, TSII, PC and PC_CO complexes. Main geometrical parameters are reported (bond in Å, angles in degree).

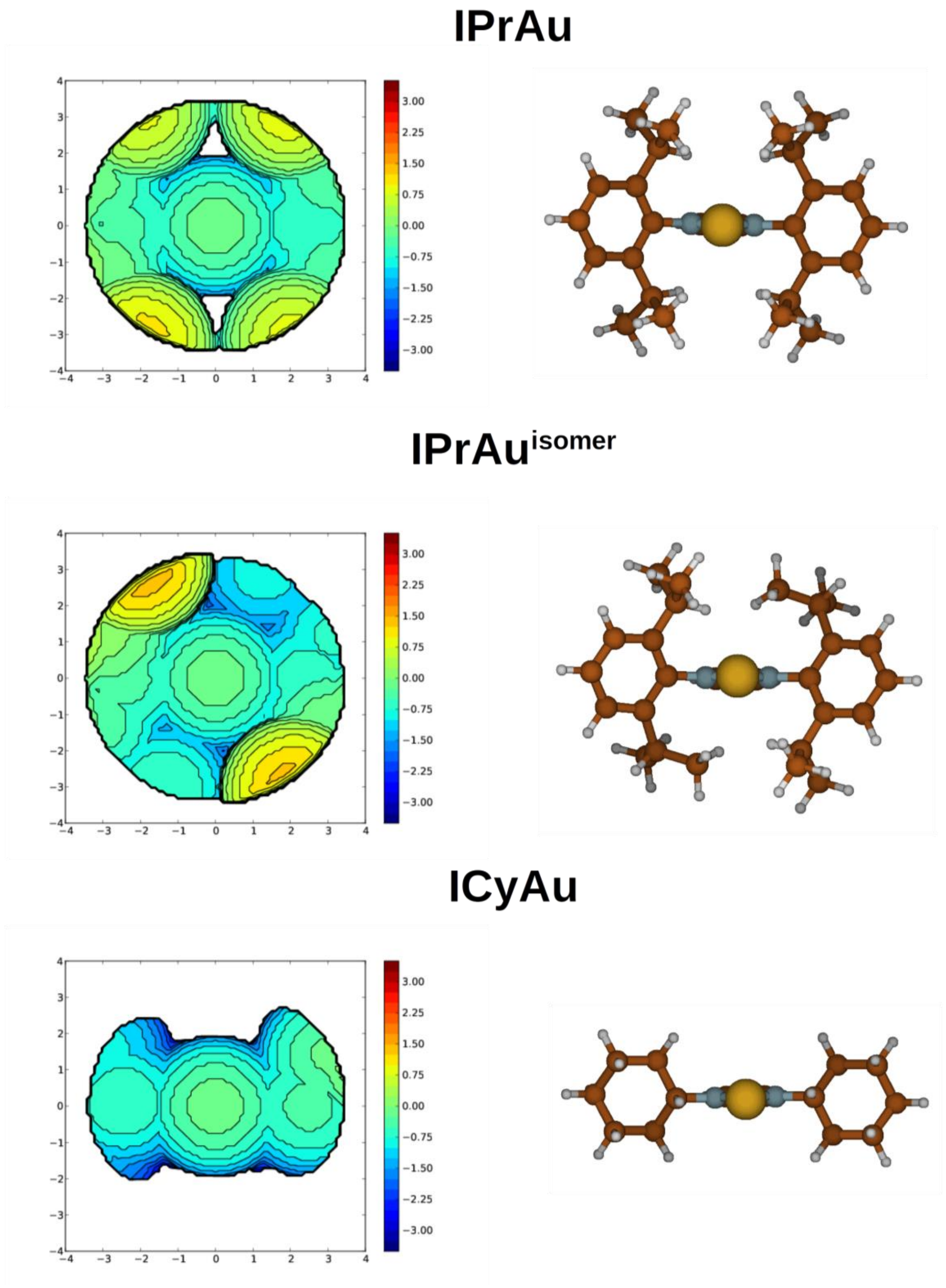

Figure S29. Steric maps (left) and structures (right) of the different NHCAu fragments (NHC=IPr, IPr^{isomer}; ICy).

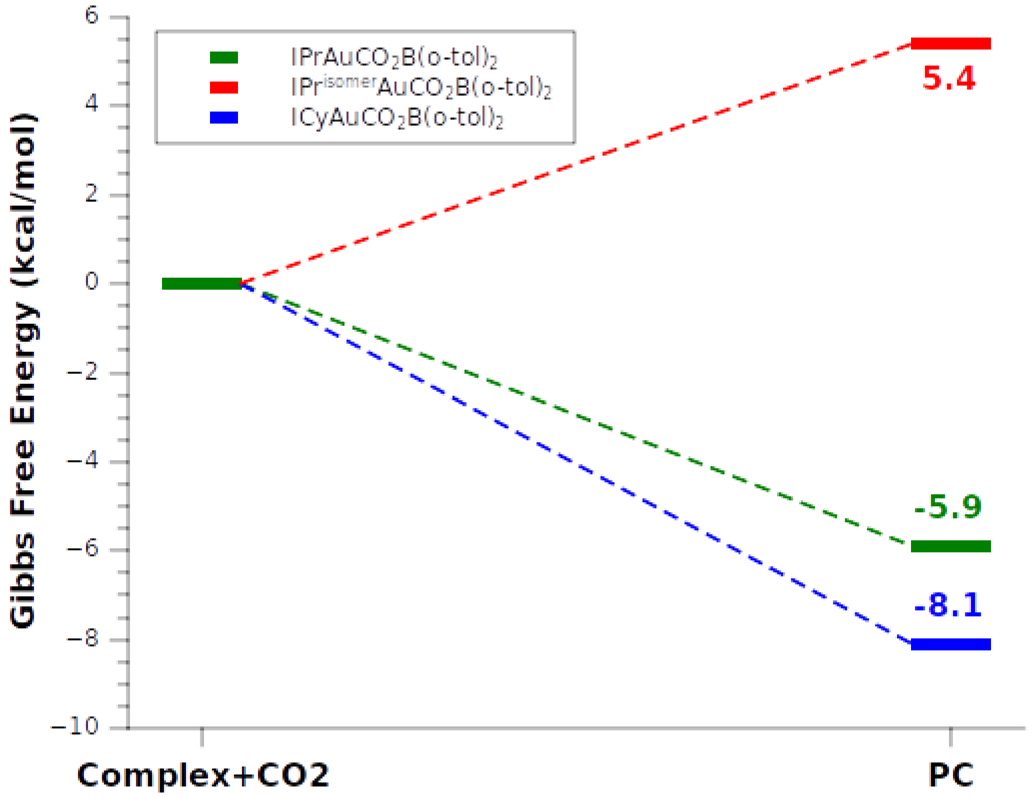

Figure S30. Gibbs' free energy profiles for the formation of insertion products PC with differently sterically hindered ligands on gold for the [LAuB(*o*-tol)$_2$] complex (L=IPr, IPr^{isomer}, ICy).

References

(1) Mitoraj, M.; Michalak, A. Natural Orbitals for Chemical Valence as Descriptors of Chemical Bonding in Transition Metal Complexes. *J. Mol. Model.* **2007**, *13* (2), 347–355.

(2) Michalak, A.; Mitoraj, M.; Ziegler, T. Bond Orbitals from Chemical Valence Theory. *J. Phys. Chem. A* **2008**, *112* (9), 1933–1939.

(3) Nalewajski, R. F.; ozek, J. Modified Valence Indices from the Two□particle Density Matrix. *Int. J. Quantum Chem.* **1994**, *51* (4), 187–200.

(4) Nalewajski, R. F.; Mrozek, J.; Michalak, A. Two-Electron Valence Indices from the Kohn-Sham Orbitals. *Int. J. Quantum Chem.* **1997**, *61* (3), 589–601.

(5) Lu, T.; Chen, F. Bond Order Analysis Based on the Laplacian of Electron Density in Fuzzy Overlap Space. *J. Phys. Chem. A* **2013**, *117* (14), 3100–3108.

(6) Bistoni, G.; Rampino, S.; Tarantelli, F.; Belpassi, L. Charge-Displacement Analysis via Natural Orbitals for Chemical Valence: Charge Transfer Effects in Coordination Chemistry. *J. Chem. Phys.* **2015**, *142* (8), 084112.

(7) Belpassi, L.; Infante, I.; Tarantelli, F.; Visscher, L. The Chemical Bond between Au(I) and the Noble Gases. Comparative Study of NgAuF and $NgAu^{+}$ (Ng = Ar, Kr, Xe) by Density Functional and Coupled Cluster Methods. *J. Am. Chem. Soc.* **2008**, *130* (3), 1048–1060.

(8) Salvi, N.; Belpassi, L.; Tarantelli, F. On the Dewar-Chatt-Duncanson Model for Catalytic Gold(I) Complexes. *Chem. - A Eur. J.* **2010**, *16* (24), 7231–7240.

(9) Bistoni, G.; Belpassi, L.; Tarantelli, F. Advances in Charge Displacement Analysis. *J. Chem. Theory Comput.* **2016**, *12* (3), 1236–1244.

(10) Morokuma, K. Molecular Orbital Studies of Hydrogen Bonds. III. C=O···H-O Hydrogen Bond in $H_2CO\cdots H_2O$ and $H_2CO\cdots 2H_2O$. *J. Chem. Phys.* **1971**, *55* (3), 1236–1244.

(11) Ziegler, T.; Rauk, A. On the Calculation of Bonding Energies by the Hartree Fock Slater Method. *Theor. Chim. Acta* **1977**, *46* (1), 1–10.

(12) Mitoraj, M. P.; Michalak, A.; Ziegler, T. A Combined Charge and Energy Decomposition Scheme for Bond Analysis. *J. Chem. Theory Comput.* **2009**, *5* (4), 962–975.

(13) Ferná, I.; Bickelhaupt, F. M. The Activation Strain Model and Molecular Orbital Theory: Understanding and Designing Chemical Reactions. *Chem. Soc. Rev* **2014**, *43*, 4953.

(14) Bickelhaupt, F. M.; Houk, K. N. Analyzing Reaction Rates with the Distortion/Interaction-Activation Strain Model. *Angewandte Chemie - International Edition*. Wiley-VCH Verlag August 14, 2017, pp 10070–10086.

(15) Vermeeren, P.; van der Lubbe, S. C. C.; Fonseca Guerra, C.; Bickelhaupt, F. M.; Hamlin, T. A. Understanding Chemical Reactivity Using the Activation Strain Model. *Nat. Protoc.* **2020**, *15* (2), 649–667.

(16) Knizia, G. Intrinsic Atomic Orbitals: An Unbiased Bridge between Quantum Theory and Chemical Concepts. *J. Chem. Theory Comput.* **2013**, *9* (11), 4834–4843.

(17) Zhao, L.; von Hopffgarten, M.; Andrada, D. M.; Frenking, G. Energy Decomposition Analysis. *Wiley Interdisciplinary Reviews: Computational Molecular Science*. Blackwell Publishing Inc. May 1, 2018.

(18) Jerabek, P.; Schwerdtfeger, P.; Frenking, G. Dative and Electron-Sharing Bonding in Transition Metal Compounds. *J. Comput. Chem.* **2019**, *40* (1), 247–264.

Geometries xyz

70

^{t}Bu$_3$PAuB(*o*-tol)$_2$ (Complex III')

B	-0.0000	0.0000	-2.0928
Au	0.0000	0.0000	0.0000
C	0.6697	2.5217	-2.6350
C	0.6738	1.1321	-2.9388
C	1.4447	0.7060	-4.0447
C	2.2263	1.5847	-4.7925
C	2.2186	2.9427	-4.4741
C	1.4348	3.3987	-3.4121
P	0.0414	0.0016	2.4212
C	0.0729	-1.7998	3.0804
C	-0.1727	3.0756	-1.5206
C	-0.6898	-1.1663	-2.8772
C	-0.5055	-2.5530	-2.6311
C	-1.2929	-3.4861	-3.3149
C	-2.2663	-3.0870	-4.2330
C	-2.4457	-1.7288	-4.5004
C	-1.6489	-0.7919	-3.8448
C	0.5622	-3.0304	-1.6873
C	1.6294	0.9199	2.9886
C	-1.5022	0.9066	3.1153
H	1.4187	4.4655	-3.1773
H	2.8124	3.6498	-5.0555
H	2.8241	1.2160	-5.6274
H	1.4320	-0.3531	-4.3100
H	-3.1911	-1.4036	-5.2279
H	-2.8722	-3.8354	-4.7461
H	-1.1392	-4.5509	-3.1234
H	-1.7757	0.2688	-4.0734
H	-1.2402	3.0767	-1.7878
H	-0.0837	2.4384	-0.6233
H	0.1162	4.1049	-1.2714
H	0.3833	-4.0629	-1.3596
H	0.6150	-2.3667	-0.8063
H	1.5557	-2.9931	-2.1602
C	-0.2231	-1.9606	4.5772
C	-0.9385	-2.6261	2.2595
C	1.4541	-2.4022	2.7727
C	-1.4005	1.3491	4.5810
C	-1.7767	2.1298	2.2173
C	-2.7260	-0.0105	2.9527
C	2.0352	0.7010	4.4514
C	2.7822	0.4869	2.0594
C	1.4382	2.4255	2.7374
H	3.6541	1.1242	2.2734
H	3.0868	-0.5527	2.2006
H	2.5060	0.6232	1.0038
H	2.4089	2.9204	2.8917
H	1.1242	2.6235	1.7038
H	0.7210	2.8880	3.4229
H	2.9212	1.3191	4.6658
H	1.2480	0.9940	5.1550
H	2.3096	-0.3403	4.6549
H	1.4065	-3.4826	2.9763
H	1.7248	-2.2727	1.7162
H	2.2498	-1.9850	3.3983
H	-0.7932	-3.6888	2.5069
H	-1.9796	-2.3745	2.4740
H	-0.7702	-2.4988	1.1815
H	-0.1244	-3.0251	4.8419
H	0.4741	-1.3961	5.2062
H	-1.2436	-1.6555	4.8346
H	-2.3605	1.7991	4.8790
H	-1.2025	0.5107	5.2583
H	-0.6248	2.1072	4.7360
H	-2.7278	2.5832	2.5360
H	-1.0032	2.8992	2.2792
H	-1.8750	1.8274	1.1654
H	-3.6260	0.5842	3.1707
H	-2.8150	-0.3866	1.9250
H	-2.7186	-0.8590	3.6444

73

RC (III')

C	-2.624041	-1.876394	-1.563480
C	-2.806180	-1.022109	-0.444011
C	-3.748019	-1.411126	0.535224
C	-4.430516	-2.624467	0.465130
C	-4.223018	-3.463678	-0.630376
C	-3.338550	-3.076651	-1.638786
B	-1.958078	0.250529	-0.113415
Au	0.134169	0.162233	-0.163051
P	2.555503	0.076571	-0.173954
C	3.222596	1.857088	-0.440841
C	-1.707368	-1.482196	-2.686336
C	-2.733240	1.551840	0.277385
C	-2.375158	2.460935	1.310247
C	-3.086790	3.657446	1.450814
C	-4.135729	3.990353	0.591266
C	-4.508662	3.103330	-0.418694
C	-3.827857	1.894079	-0.548359
C	-1.273052	2.140929	2.280071
C	3.188247	-0.597139	1.508038
C	3.176451	-1.060226	-1.589254
H	-3.194177	-3.726441	-2.505101
H	-4.756713	-4.412174	-0.707783
H	-5.132179	-2.906027	1.251927
H	-3.925212	-0.746904	1.383854
H	-5.335700	3.344306	-1.088330
H	-4.664614	4.936225	0.718657
H	-2.810772	4.346509	2.252272
H	-4.137263	1.188357	-1.322281
H	-2.185127	-0.744293	-3.349142
H	-0.802316	-0.995387	-2.282411
H	-1.417220	-2.346993	-3.297183
H	-0.979812	3.023740	2.862880
H	-0.394754	1.745609	1.739930
H	-1.575059	1.350330	2.983141
C	4.699175	1.957096	-0.843872
C	2.343683	2.533269	-1.513161
C	2.994884	2.662892	0.849753
C	4.653435	-1.467250	-1.509285
C	2.290377	-2.322607	-1.613094
C	2.923611	-0.344707	-2.927069
C	4.665090	-0.314771	1.812206
C	2.302443	-0.001677	2.621150
C	2.952887	-2.116241	1.550432
H	2.582686	-0.476206	3.574040
H	2.419906	1.078262	2.739614
H	1.240137	-0.210418	2.433942
H	3.122807	-2.458365	2.582352
H	1.922966	-2.376001	1.275646
H	3.639498	-2.671572	0.903349
H	4.924254	-0.796529	2.768113
H	5.334554	-0.719297	1.044629
H	4.872687	0.755375	1.921394
H	3.211146	3.719543	0.632072
H	1.950844	2.599017	1.184260
H	3.651403	2.355347	1.669791
H	2.616967	3.598584	-1.559697
H	2.475259	2.112305	-2.512691
H	1.278741	2.462622	-1.248616
H	4.972361	3.021723	-0.914632
H	5.367245	1.490773	-0.111084
H	4.893734	1.506640	-1.823744
H	4.899721	-2.070776	-2.397107

H	5.327655	-0.603795	-1.498319
H	4.868609	-2.083501	-0.629122
H	2.516175	-2.880538	-2.534663
H	2.465744	-2.992512	-0.768305
H	1.224264	-2.059402	-1.624260
H	3.103225	-1.066630	-3.737932
H	1.884022	-0.000080	-3.010912
H	3.594717	0.504904	-3.089072
C	-0.911220	-2.631915	1.831312
O	-1.156095	-1.915411	2.724323
O	-0.649273	-3.367704	0.960504

73

TSI (III')

C	-3.097823	1.903242	1.009453
C	-3.194556	0.666774	0.322401
C	-4.483565	0.189328	0.002791
C	-5.639488	0.898468	0.316929
C	-5.530174	2.112184	0.996133
C	-4.269033	2.598100	1.339936
B	-1.987226	-0.225954	-0.136929
Au	0.076009	0.282287	-0.266587
P	2.402595	0.190256	0.291070
C	2.694877	-1.501092	1.150040
C	-1.774237	2.493474	1.416055
C	-2.047122	-1.781412	0.082976
C	-1.465077	-2.813589	-0.705456
C	-1.478992	-4.129412	-0.229677
C	-2.031935	-4.462997	1.007796
C	-2.604831	-3.463858	1.792045
C	-2.621050	-2.152284	1.322069
C	-0.837846	-2.540793	-2.042072
C	3.449268	0.300130	-1.312013
C	2.873570	1.619803	1.476693
H	-4.186568	3.545520	1.876626
H	-6.423558	2.679641	1.261272
H	-6.618530	0.504333	0.041233
H	-4.569284	-0.765307	-0.518765
H	-3.048922	-3.703321	2.759271
H	-2.017489	-5.498414	1.351427
H	-1.037204	-4.913858	-0.848079
H	-3.082100	-1.377726	1.936436
H	-1.216545	1.817544	2.080045
H	-1.139174	2.671870	0.535666
H	-1.914139	3.447753	1.939937
H	-0.304085	-3.424779	-2.413279
H	-0.129267	-1.698997	-1.973432
H	-1.587239	-2.241441	-2.785802
C	4.024888	-1.606238	1.907861
C	1.521906	-1.772611	2.114412
C	2.628918	-2.616817	0.093410
C	4.381755	1.850387	1.641581
C	2.206059	2.911761	0.965823
C	2.250596	1.337826	2.853999
C	4.899939	-0.177265	-1.162830
C	2.740042	-0.516335	-2.410208
C	3.442830	1.756673	-1.805614
H	3.301669	-0.384564	-3.347385
H	2.688499	-1.586653	-2.199186
H	1.718614	-0.147795	-2.575605
H	3.881502	1.771619	-2.814469
H	2.422188	2.154594	-1.881636
H	4.042866	2.422827	-1.177459
H	5.420987	-0.008477	-2.117817
H	5.444731	0.370967	-0.386425
H	4.966252	-1.248117	-0.941165
H	2.637905	-3.580235	0.624621
H	1.697955	-2.570439	-0.485933
H	3.482375	-2.611598	-0.591871
H	1.656036	-2.782305	2.531400
H	1.471642	-1.071160	2.950567
H	0.559439	-1.753273	1.585499
H	4.118063	-2.629038	2.304134
H	4.892071	-1.419816	1.264573
H	4.074012	-0.921797	2.761996
H	4.531797	2.650521	2.382899
H	4.907410	0.960003	2.003405
H	4.856568	2.178114	0.710085
H	2.353274	3.693974	1.725856
H	2.626228	3.277585	0.026684
H	1.126583	2.771544	0.826876
H	2.374197	2.239605	3.471901
H	1.174468	1.132816	2.776742
H	2.733645	0.508958	3.381242
C	-1.448075	0.994188	-2.049673
O	-2.253701	0.156121	-2.407792
O	-0.963503	2.073058	-2.279276

73

INT (III')

C	-3.972440	-0.988964	-1.624645
C	-3.766044	0.016572	-0.643792
C	-4.649171	1.113675	-0.616282
C	-5.726026	1.220789	-1.494580
C	-5.918990	0.228192	-2.454940
C	-5.041527	-0.855359	-2.519028
B	-2.621831	-0.036480	0.428189
Au	0.615581	-1.027649	0.586905
P	2.610913	-0.411083	-0.469688
C	2.225429	0.298972	-2.206593
C	-3.065992	-2.186853	-1.752926
C	-1.902944	1.286022	0.883159
C	-1.590314	1.619161	2.225292
C	-0.969836	2.847011	2.494008
C	-0.642295	3.740579	1.475760
C	-0.954686	3.427369	0.152818
C	-1.593558	2.222233	-0.125352
C	-1.928324	0.718198	3.384823
C	3.493665	0.908340	0.598665
C	3.709435	-1.973172	-0.634002
H	-5.188420	-1.619988	-3.284961
H	-6.747714	0.298142	-3.161152
H	-6.402494	2.074329	-1.437350
H	-4.485839	1.898531	0.124669
H	-0.716587	4.123756	-0.652232
H	-0.157068	4.687215	1.718276
H	-0.748237	3.109731	3.530636
H	-1.865410	1.988361	-1.156397
H	-2.005627	-1.903462	-1.712568
H	-3.227970	-2.896689	-0.929933
H	-3.250268	-2.709435	-2.700118
H	-2.114842	1.310597	4.289939
H	-1.097760	0.031530	3.606862
H	-2.808436	0.095821	3.186811
C	3.430108	0.355733	-3.155127
C	1.100804	-0.548857	-2.834978
C	1.643648	1.712776	-2.048176
C	5.182612	-1.687879	-0.953678
C	3.612852	-2.779897	0.676660
C	3.115287	-2.877410	-1.727347
C	4.621401	1.663372	-0.116538
C	2.429666	1.904780	1.098536
C	4.060336	0.226625	1.855553
H	2.909568	2.584320	1.819140
H	1.992430	2.515273	0.306493
H	1.607629	1.388725	1.613121
H	4.400637	1.016852	2.541167
H	3.293924	-0.361285	2.377868
H	4.920683	-0.415810	1.642030
H	5.087717	2.353849	0.603111

H	5.405087	0.994673	-0.489544
H	4.254224	2.266812	-0.953875
H	1.260748	2.029750	-3.029969
H	0.804459	1.730658	-1.342130
H	2.389725	2.449705	-1.734121
H	0.810209	-0.077940	-3.786156
H	1.394109	-1.579400	-3.047834
H	0.217761	-0.571559	-2.181116
H	3.110872	0.835813	-4.092854
H	4.257247	0.946580	-2.745580
H	3.809800	-0.639029	-3.412608
H	5.702267	-2.649243	-1.086409
H	5.311270	-1.111297	-1.876189
H	5.687575	-1.155878	-0.139552
H	4.138947	-3.734788	0.525994
H	4.071629	-2.278434	1.531820
H	2.566897	-3.001931	0.929020
H	3.648399	-3.839220	-1.695605
H	2.051352	-3.081115	-1.546471
H	3.235266	-2.469135	-2.735687
C	-1.095062	-1.710556	1.504557
O	-2.327721	-1.278787	0.950921
O	-1.172598	-2.546523	2.380676

73

TSII (III')

C	-3.853416	2.549010	0.269861
C	-4.241755	1.185847	0.379938
C	-5.582759	0.905082	0.716046
C	-6.521306	1.907978	0.945132
C	-6.130579	3.240784	0.822830
C	-4.811985	3.547100	0.485909
B	-3.288845	-0.047819	0.244995
Au	0.887488	-0.007592	-0.174045
P	3.223117	0.011692	0.018107
C	3.732950	-1.069596	1.514971
C	-2.453110	2.968048	-0.091812
C	-3.824071	-1.509317	0.051070
C	-4.660088	-1.909863	-1.019718
C	-5.104941	-3.235875	-1.075271
C	-4.759980	-4.163676	-0.090626
C	-3.931992	-3.778199	0.962958
C	-3.461351	-2.465902	1.015878
C	-5.034233	-0.951943	-2.119985
C	3.990203	-0.691559	-1.590363
C	3.805293	1.817998	0.285266
H	-4.513456	4.592920	0.387775
H	-6.849780	4.044500	0.988461
H	-7.547864	1.652876	1.210392
H	-5.887553	-0.138964	0.808162
H	-3.645823	-4.496656	1.732511
H	-5.128377	-5.188667	-0.155691
H	-5.731969	-3.548239	-1.913258
H	-2.795617	-2.169056	1.828682
H	-1.737142	2.675417	0.688441
H	-2.121305	2.488143	-1.021299
H	-2.397805	4.056071	-0.220949
H	-5.495665	-1.483435	-2.961840
H	-4.147772	-0.413809	-2.481955
H	-5.745374	-0.191793	-1.766229
C	5.172784	-0.856911	1.999809
C	2.748382	-0.792399	2.668722
C	3.537976	-2.549320	1.143709
C	5.305428	2.047681	0.058030
C	2.990724	2.733035	-0.651720
C	3.439195	2.248427	1.715962
C	5.466188	-1.093499	-1.474286
C	3.151274	-1.907292	-2.033123
C	3.837546	0.353078	-2.708807
H	3.518197	-2.232157	-3.018552
H	3.224505	-2.759154	-1.353398
H	2.090798	-1.638607	-2.135514
H	4.117106	-0.127560	-3.658100
H	2.798638	0.695790	-2.803410
H	4.491249	1.220844	-2.575858
H	5.815511	-1.424611	-2.464382
H	6.105482	-0.261451	-1.158812
H	5.618048	-1.928234	-0.781153
H	3.664746	-3.145319	2.059699
H	2.527068	-2.739921	0.759573
H	4.271129	-2.908648	0.414647
H	2.962295	-1.507875	3.477151
H	2.834782	0.214320	3.083570
H	1.709056	-0.941805	2.344569
H	5.372151	-1.566267	2.817632
H	5.913420	-1.040925	1.213614
H	5.334001	0.151111	2.397729
H	5.536530	3.097164	0.296453
H	5.929635	1.416913	0.700671
H	5.600299	1.878696	-0.983537
H	3.234097	3.777083	-0.403206
H	3.212637	2.582100	-1.710489
H	1.911368	2.588015	-0.503214
H	3.614153	3.331587	1.796902
H	2.379046	2.064571	1.934966
H	4.052364	1.759484	2.479799
C	-1.149860	0.002456	-0.387793
O	-1.914275	0.070054	0.721684
O	-1.787503	-0.005088	-1.459996

73

PC (III')

C	-5.323436	0.951931	0.862247
C	-3.985315	1.200649	0.503135
C	-3.526981	2.542602	0.578672
C	-4.412442	3.549723	0.987833
C	-5.734838	3.273658	1.334816
C	-6.194765	1.960151	1.274839
B	-3.105870	-0.071320	0.091472
O	-1.913793	0.187394	-0.943153
C	-1.111251	-0.088234	0.046142
Au	0.922987	-0.082963	-0.002190
P	3.260226	-0.037638	-0.055060
C	3.923720	-1.613621	0.805294
C	-2.114145	2.947714	0.238309
C	-3.870683	-1.423416	-0.256597
C	-4.610181	-1.565138	-1.454823
C	-5.276162	-2.769019	-1.714988
C	-5.234019	-3.833375	-0.812287
C	-4.512526	-3.700306	0.373181
C	-3.838577	-2.505249	0.636231
C	-4.687477	-0.438245	-2.452558
O	-1.874118	-0.333786	1.076881
C	3.835427	0.014416	-1.880370
C	3.855552	1.528034	0.873915
H	-5.840558	-2.871962	-2.644849
H	-5.763479	-4.760822	-1.036495
H	-4.472024	-4.522454	1.089569
H	-3.269805	-2.408561	1.562827
H	-7.225176	1.720449	1.541487
H	-6.397578	4.081650	1.648898
H	-4.049574	4.579053	1.036066
H	-5.693513	-0.073495	0.808422
H	-5.110542	0.467063	-1.993009
H	-3.687620	-0.166373	-2.819635
H	-5.310032	-0.712677	-3.313530
H	-1.987194	4.031063	0.358840
H	-1.851876	2.678498	-0.792400
H	-1.383549	2.448841	0.891645
C	5.339621	1.507845	1.262135

C	3.578947	2.761402	-0.002394
C	2.987069	1.703769	2.135884
C	5.302172	0.420902	-2.072127
C	3.607930	-1.369973	-2.510041
C	2.918752	0.984848	-2.652034
C	5.401832	-1.914252	0.525599
C	3.711969	-1.475892	2.322774
C	3.054842	-2.809233	0.365380
H	3.780667	-1.278895	-3.592623
H	4.296861	-2.131404	-2.131044
H	2.575733	-1.716189	-2.366594
H	3.172597	0.912883	-3.720265
H	1.861683	0.708648	-2.534327
H	3.036138	2.028462	-2.352178
H	5.540704	0.362530	-3.145069
H	5.495709	1.450661	-1.752269
H	5.995052	-0.242148	-1.542362
H	3.366981	-3.687669	0.950024
H	1.991958	-2.623429	0.573231
H	3.158204	-3.059785	-0.692602
H	3.937905	-2.449184	2.782981
H	4.372883	-0.734478	2.782759
H	2.670119	-1.228246	2.565390
H	5.692950	-2.798854	1.112435
H	5.588358	-2.148515	-0.528137
H	6.061419	-1.090246	0.819455
H	5.587098	2.470354	1.735156
H	5.570707	0.720690	1.988089
H	5.999156	1.384763	0.395950
H	3.764237	3.655997	0.610309
H	4.234358	2.821515	-0.876964
H	2.532839	2.796694	-0.334554
H	3.259838	2.661345	2.604441
H	1.919398	1.742217	1.878818
H	3.130471	0.916330	2.879118

73

TS_CO (III')

C	3.291359	-3.384702	-0.386666
C	3.357924	-2.130392	0.274818
C	4.254545	-2.009705	1.353524
C	5.059387	-3.064045	1.786104
C	4.988376	-4.288428	1.121952
C	4.112711	-4.435856	0.045030
B	2.461009	-0.863662	-0.115941
Au	-0.577470	0.602770	-0.708011
P	-2.490022	-0.107574	0.359935
C	-2.142658	-1.847833	1.087640
C	2.363297	-3.629027	-1.547335
C	2.836128	0.550618	0.551990
C	3.934992	1.327024	0.116930
C	4.150852	2.596817	0.667715
C	3.310382	3.112808	1.657586
C	2.229204	2.352948	2.103777
C	2.006685	1.090660	1.547205
C	4.855458	0.803166	-0.955451
C	-2.926681	1.130292	1.754512
C	-3.917848	-0.200744	-0.915565
H	4.063903	-5.393704	-0.479187
H	5.614039	-5.126022	1.435498
H	5.740654	-2.930913	2.628362
H	4.319330	-1.047499	1.867405
H	1.565371	2.739568	2.879384
H	3.502353	4.101769	2.076633
H	4.995196	3.193630	0.313870
H	1.159210	0.500534	1.904997
H	1.316395	-3.651295	-1.212912
H	2.415419	-2.813812	-2.279113
H	2.597180	-4.584562	-2.036470
H	5.664106	1.512671	-1.173055
H	4.304069	0.615417	-1.889038
H	5.303293	-0.155887	-0.657297
C	-3.401287	-2.620565	1.505291
C	-1.342047	-2.662848	0.051445
C	-1.214488	-1.695291	2.304097
C	-5.308327	-0.320240	-0.275794
C	-3.869601	1.044415	-1.821050
C	-3.682486	-1.408076	-1.838301
C	-3.914814	0.576736	2.790328
C	-1.622675	1.557128	2.454171
C	-3.509213	2.408199	1.129492
H	-1.873005	2.332803	3.193454
H	-1.123756	0.742044	2.983336
H	-0.912633	1.989473	1.736090
H	-3.591387	3.162565	1.925938
H	-2.849039	2.812561	0.351195
H	-4.510263	2.262591	0.711362
H	-4.144313	1.378192	3.508704
H	-4.860291	0.254052	2.340988
H	-3.497503	-0.260448	3.360087
H	-0.898528	-2.703120	2.610533
H	-0.306169	-1.134632	2.048597
H	-1.701338	-1.226459	3.165114
H	-1.059947	-3.613477	0.529963
H	-1.914231	-2.906662	-0.846733
H	-0.414958	-2.151397	-0.248263
H	-3.083159	-3.565748	1.970601
H	-4.009910	-2.079620	2.238438
H	-4.035069	-2.879004	0.649804
H	-6.047461	-0.444983	-1.081527
H	-5.394649	-1.186359	0.388877
H	-5.589469	0.577733	0.285249
H	-4.635355	0.921494	-2.601769
H	-4.078124	1.977487	-1.292910
H	-2.890915	1.138447	-2.309163
H	-4.413008	-1.349568	-2.658688
H	-2.679077	-1.390495	-2.283714
H	-3.831615	-2.367278	-1.333331
C	0.908013	1.344936	-1.823914
O	1.422025	-0.956430	-0.934358
O	1.764424	1.678739	-2.504419

73

PC_CO (III')

C	-2.286046	1.937533	0.465952
C	-2.211475	0.603774	0.899654
C	-1.794941	0.355893	2.227588
C	-1.454523	1.428129	3.060768
C	-1.529674	2.747031	2.608336
C	-1.950415	3.003721	1.303154
B	-2.531569	-0.593452	-0.110643
Au	0.361660	-0.412961	-0.667261
P	2.462136	0.384585	-0.481342
C	3.575832	-0.383730	-1.837473
C	3.189799	0.222151	-3.197272
C	-1.715186	-1.060088	2.738095
C	-4.015045	-1.147854	-0.204772
C	-4.434656	-2.183656	-1.083518
C	-5.771721	-2.602853	-1.057641
C	-6.704582	-2.032731	-0.190096
C	-6.307606	-1.014933	0.676151
C	-4.980231	-0.588399	0.655943
C	-3.492645	-2.853972	-2.048082
C	2.381544	2.289012	-0.697111
C	1.763308	2.904026	0.569334
C	3.160239	-0.040426	1.253357
C	3.542174	-1.529703	1.295327
C	3.735642	2.954634	-0.978677
C	1.398993	2.612668	-1.840427
C	4.377957	0.797893	1.665458

C	2.029927	0.134890	2.286038
C	5.081621	-0.192045	-1.609075
C	3.253114	-1.887057	-1.937350
H	-6.088140	-3.398303	-1.736456
H	-7.737971	-2.383753	-0.194938
H	-7.025264	-0.557489	1.359004
H	-4.669304	0.211204	1.331544
H	-2.015068	4.029959	0.937552
H	-1.261070	3.569520	3.272972
H	-1.125944	1.224464	4.082803
H	-2.612346	2.148803	-0.555787
H	-3.007503	-2.121057	-2.705681
H	-2.674115	-3.356801	-1.516351
H	-4.027915	-3.591031	-2.661296
H	-1.316149	-1.099852	3.759576
H	-1.073027	-1.676516	2.091028
H	-2.707466	-1.536111	2.737357
H	2.416413	-0.180407	3.267373
H	1.670393	1.161670	2.379690
H	1.169130	-0.498387	2.036678
H	3.770911	-1.784918	2.340944
H	2.714240	-2.170048	0.967720
H	4.432467	-1.760119	0.701309
H	4.734380	0.429041	2.639440
H	5.208330	0.711497	0.955880
H	4.136324	1.858821	1.789324
H	1.565402	3.966746	0.363384
H	0.803446	2.436528	0.822853
H	2.427634	2.857947	1.438057
H	1.299524	3.707267	-1.899559
H	1.729539	2.253525	-2.817909
H	0.406456	2.190082	-1.633156
H	3.582066	4.043846	-1.017913
H	4.476311	2.753496	-0.196992
H	4.159271	2.652119	-1.942866
H	5.618551	-0.615861	-2.471498
H	5.365619	0.862913	-1.528421
H	5.438306	-0.716281	-0.715821
H	3.819234	-2.298828	-2.786709
H	3.527800	-2.453159	-1.044701
H	2.183182	-2.046328	-2.125667
H	3.710165	-0.348822	-3.980629
H	2.110911	0.137961	-3.383483
H	3.490077	1.269869	-3.300162
O	-1.548936	-1.118646	-0.860972
C	0.569940	-3.586620	0.133298
O	-0.425882	-4.131088	0.070194

119

IPrAuAl(NON') (Complex I')

C	3.824449	-2.509530	0.251548
C	3.898869	-1.272804	-0.411446
C	4.061130	-1.152697	-1.801153
C	4.166575	-2.337598	-2.538580
C	4.107745	-3.578166	-1.910663
C	3.933633	-3.662560	-0.532013
N	3.788463	-0.067757	0.369052
C	4.861067	0.599255	0.946784
C	4.342786	1.691924	1.571651
N	2.972726	1.653718	1.351939
C	2.610108	0.571548	0.608228
Au	0.643799	0.051183	0.021407
Al	-1.630869	-0.474102	-0.562489
N	-2.502989	0.705296	-1.825720
C	-1.806552	1.163863	-2.953735
C	2.033458	2.631451	1.835542
C	1.437558	2.422867	3.090369
C	0.529056	3.388394	3.537108
C	0.227408	4.502156	2.758091
C	0.820229	4.668909	1.509458
C	1.737009	3.733842	1.016795
C	1.696055	1.170833	3.908246
C	2.109422	1.489298	5.350140
C	2.314472	3.881328	-0.378819
C	2.940020	5.261805	-0.610938
C	4.057668	0.194067	-2.500270
C	5.256367	0.365483	-3.441370
C	3.555854	-2.602688	1.742353
C	4.461258	-3.621978	2.443693
N	-2.235511	-2.284029	-0.219484
C	-3.402082	-2.407233	0.522537
C	-3.998832	-1.199115	0.918633
C	-5.232032	-1.031121	1.521811
C	-5.925314	-2.205806	1.846805
C	-5.363269	-3.444998	1.520626
C	-4.136610	-3.563534	0.863876
O	-3.289692	-0.046357	0.504034
C	-4.139960	0.888104	-0.136545
C	-5.372358	1.157127	0.428237
C	-5.725822	0.409199	1.721159
C	-6.196928	2.043104	-0.280820
C	-5.753533	2.559644	-1.503827
C	-4.529194	2.200981	-2.071724
C	-3.674032	1.304988	-1.393898
C	-7.225370	0.459339	2.013351
C	-4.959105	1.048935	2.906820
C	-1.341370	-3.344147	-0.408283
C	0.463611	0.253186	3.862921
C	1.233867	3.568115	-1.424531
C	2.726101	0.397499	-3.237744
C	2.069946	-2.914644	1.982812
H	-3.772103	-4.546423	0.574105
H	-6.900796	-2.158634	2.326887
H	-7.180163	2.311513	0.100793
H	-4.257183	2.573888	-3.057179
H	-7.551491	1.498013	2.151491
H	-7.450561	-0.080573	2.941736
H	-7.811994	0.014827	1.199135
H	-5.276711	2.092605	3.035606
H	-3.875542	1.034988	2.738106
H	-5.174529	0.497447	3.832097
H	-5.913105	-4.355423	1.763450
H	-6.401165	3.244354	-2.053510
H	4.814443	2.481103	2.143122
H	5.878382	0.239409	0.859646
H	0.041882	3.258558	4.503751
H	-0.485515	5.242283	3.123555
H	0.558053	5.534123	0.900103
H	4.286172	-2.286058	-3.621057
H	4.186371	-4.490284	-2.503808
H	3.863667	-4.641158	-0.058364
H	3.112459	3.134979	-0.497434
H	2.526833	0.624852	3.440328
H	4.130371	0.976171	-1.731589
H	3.766150	-1.619076	2.186180
H	2.685423	1.391401	-3.703808
H	2.599954	-0.355052	-4.029138
H	1.872981	0.307965	-2.550047
H	5.252095	1.374502	-3.876526
H	6.207247	0.223548	-2.909748
H	5.222717	-0.352637	-4.272583
H	4.286276	-3.594545	3.527952
H	4.256121	-4.646856	2.104545
H	5.523558	-3.409864	2.260514
H	1.852872	-2.940050	3.060099
H	1.424845	-2.156740	1.513320
H	1.801215	-3.886711	1.548161
H	2.341902	0.560544	5.889303
H	2.997807	2.135081	5.377515
H	1.303865	1.998198	5.897617

H	0.662864	-0.683938	4.401281
H	-0.403334	0.742314	4.330229
H	0.199081	0.008625	2.823269
H	1.650578	3.607154	-2.440289
H	0.808895	2.566109	-1.267407
H	0.411322	4.294897	-1.365316
H	3.393399	5.305912	-1.610810
H	2.187486	6.060648	-0.554130
H	3.720899	5.478234	0.131048
C	-1.163644	0.220968	-3.777115
C	-0.389146	0.623256	-4.863020
C	-0.245615	1.978189	-5.164613
C	-0.878556	2.924523	-4.353088
C	-1.644527	2.528577	-3.260200
H	-1.301053	-0.841138	-3.566706
H	0.097970	-0.130556	-5.482938
H	0.354111	2.294408	-6.018281
H	-0.760232	3.988758	-4.563521
H	-2.096571	3.278849	-2.612644
C	-1.167941	-4.404674	0.504003
C	-0.207109	-5.388025	0.278528
C	0.627650	-5.335523	-0.840500
C	0.486534	-4.274078	-1.736101
C	-0.488818	-3.303350	-1.531111
H	-1.767175	-4.439269	1.411851
H	-0.097188	-6.196916	1.002964
H	1.383013	-6.103813	-1.005951
H	1.140676	-4.198551	-2.604981
H	-0.599129	-2.490466	-2.251077

122

RC (I’)

C	0.974800	3.579510	-0.912675
C	1.757000	3.286232	0.222983
C	1.687485	4.178040	1.312170
C	0.895420	5.322223	1.245672
C	0.131149	5.602518	0.110556
C	0.167843	4.711489	-0.963159
N	2.477753	2.085559	0.238381
C	3.623993	1.911157	1.001615
C	4.034736	0.580057	1.181019
C	5.209356	0.134796	1.759428
C	6.044626	1.124552	2.297561
C	5.671423	2.468871	2.190494
C	4.497705	2.874154	1.550912
C	5.493471	-1.373374	1.705467
C	5.083649	-1.829825	0.298677
C	3.923129	-1.291032	-0.224773
O	3.189203	-0.367345	0.557604
C	5.799236	-2.692093	-0.545240
C	5.334173	-2.919332	-1.845914
C	4.196435	-2.292439	-2.357604
C	3.452675	-1.411543	-1.543224
Al	1.642688	0.471230	-0.435081
N	2.393376	-0.583992	-1.874176
C	1.710054	-0.699371	-3.095075
C	1.280073	0.477181	-3.735651
C	0.528981	0.425315	-4.907955
C	0.196221	-0.804194	-5.477796
C	0.616949	-1.980065	-4.849505
C	1.358492	-1.934374	-3.671752
C	6.962253	-1.683499	1.995088
C	4.608404	-2.094657	2.753430
Au	-0.717433	0.184646	-0.036143
C	-2.787789	-0.090479	0.334812
N	-3.802726	0.810264	0.209146
C	-5.027943	0.257712	0.557677
C	-4.780899	-1.031055	0.915603
N	-3.412537	-1.220674	0.771882
C	-3.638317	2.172417	-0.228764
C	-3.405272	3.161974	0.741322
C	-3.294920	4.482485	0.294762
C	-3.409179	4.793341	-1.057092
C	-3.618495	3.787346	-1.995535
C	-3.737362	2.449387	-1.602028
C	-3.196749	2.816603	2.204074
C	-1.693104	2.823409	2.523707
C	-3.892572	1.348882	-2.634997
C	-2.544697	1.095744	-3.326763
C	-2.740999	-2.461824	1.054271
C	-2.236649	-2.669997	2.348818
C	-1.622753	-3.900392	2.606867
C	-1.511679	-4.867778	1.612183
C	-2.003170	-4.621839	0.332034
C	-2.631028	-3.410218	0.022658
C	-2.283103	-1.591874	3.415693
C	-0.881437	-0.990950	3.609740
C	-3.102433	-3.116035	-1.389813
C	-1.902242	-2.734064	-2.269813
C	-2.867822	-2.101359	4.738410
C	-3.898377	-4.274650	-2.001896
C	-4.999711	1.646595	-3.652945
C	-3.973489	3.740887	3.148953
H	4.281744	3.934288	1.438412
H	6.985661	0.856620	2.773878
H	6.719322	-3.163386	-0.205317
H	3.910269	-2.447144	-3.395859
H	7.137678	-2.765855	1.952262
H	7.229675	-1.348264	3.005192
H	7.630018	-1.192544	1.275491
H	4.784140	-3.178027	2.705253
H	3.542752	-1.910622	2.574688
H	4.861236	-1.740162	3.761987
H	6.332364	3.235020	2.598415
H	5.897871	-3.587327	-2.498903
H	-5.439330	-1.820566	1.254658
H	-5.947706	0.827290	0.518346
H	-1.212375	-4.097143	3.597342
H	-1.027303	-5.819832	1.833108
H	-1.889113	-5.379819	-0.442738
H	-3.683667	4.042789	-3.053313
H	-3.317508	5.829738	-1.384648
H	-3.100195	5.276885	1.014491
H	-3.771811	-2.245170	-1.347867
H	-2.937181	-0.785678	3.055452
H	-4.170294	0.425093	-2.108162
H	-3.567147	1.794499	2.367796
H	-2.620186	0.262703	-4.038710
H	-2.218874	1.988929	-3.879053
H	-1.762143	0.848441	-2.594659
H	-5.120083	0.794754	-4.336373
H	-5.962915	1.829665	-3.157351
H	-4.760308	2.527366	-4.264973
H	-3.847752	3.405661	4.187678
H	-3.609689	4.776161	3.092129
H	-5.047020	3.744863	2.915718
H	-1.521428	2.531623	3.569545
H	-1.147698	2.124182	1.871964
H	-1.265577	3.822329	2.363397
H	-2.935766	-1.277109	5.461611
H	-3.874142	-2.518821	4.596566
H	-2.236706	-2.881938	5.185422
H	-0.913350	-0.175881	4.346228
H	-0.176023	-1.754577	3.967096
H	-0.494866	-0.590009	2.660777
H	-2.232169	-2.463424	-3.282198
H	-1.353478	-1.878128	-1.849872
H	-1.198009	-3.573247	-2.352264
H	-4.283938	-3.983371	-2.988529
H	-3.271541	-5.165707	-2.145386

H	-4.750607	-4.556889	-1.368688
H	1.567884	1.441255	-3.312500
H	0.209914	1.354145	-5.382481
H	-0.385598	-0.847298	-6.398731
H	0.348590	-2.949301	-5.273021
H	1.639208	-2.858590	-3.169478
H	2.231536	3.954901	2.228128
H	0.862271	5.994741	2.104654
H	-0.492463	6.495544	0.068392
H	-0.437285	4.896830	-1.850857
H	1.006461	2.901287	-1.767094
C	1.293047	-3.476644	0.227057
O	1.230427	-4.040432	-0.797005
O	1.362347	-2.923056	1.254314

122

TSI (I')

C	-2.208515	-4.613868	1.249798
C	-1.576610	-3.375357	1.435405
C	-0.339156	-3.338315	2.093715
C	0.263829	-4.515541	2.538165
C	-0.369707	-5.744791	2.349391
C	-1.611175	-5.787347	1.707651
N	-2.136558	-2.177295	0.923866
C	-3.296005	-1.691422	1.498478
C	-3.771991	-0.477368	0.979958
C	-4.967297	0.148365	1.270155
C	-5.741811	-0.449843	2.279037
C	-5.273951	-1.611773	2.904333
C	-4.080643	-2.240621	2.534032
C	-5.318351	1.409307	0.465133
C	-4.842103	1.174112	-0.976307
C	-3.650872	0.497514	-1.149502
O	-2.937815	0.050450	-0.022473
C	-5.500023	1.565546	-2.154321
C	-4.938659	1.240019	-3.395153
C	-3.763026	0.493896	-3.517679
C	-3.090745	0.056451	-2.357255
Al	-1.335091	-1.117702	-0.472249
N	-2.011227	-0.803978	-2.254648
C	-1.280765	-1.081095	-3.438681
C	-1.226192	-2.382010	-3.957523
C	-0.469194	-2.659492	-5.096743
C	0.232784	-1.640554	-5.743322
C	0.176227	-0.339378	-5.236548
C	-0.569250	-0.062400	-4.093186
C	-6.815431	1.716582	0.521302
C	-4.528429	2.603976	1.055337
Au	0.764392	0.004005	0.038969
C	2.325306	1.327281	0.645569
N	3.678831	1.186652	0.580019
C	4.334805	2.298707	1.093613
C	3.365256	3.163722	1.498091
N	2.151627	2.551139	1.216014
C	4.340494	0.024996	0.043496
C	4.701728	-1.005202	0.928928
C	5.340664	-2.123213	0.384008
C	5.600643	-2.204185	-0.981630
C	5.223256	-1.170366	-1.832372
C	4.584585	-0.027448	-1.338022
C	4.345208	-0.950000	2.403821
C	3.041218	-1.726017	2.649313
C	4.135689	1.073172	-2.281269
C	2.925846	0.605314	-3.102851
C	0.863990	3.140029	1.481939
C	0.227885	2.845836	2.699318
C	-1.003258	3.464342	2.945060
C	-1.569617	4.328610	2.013195
C	-0.929799	4.573820	0.801218
C	0.301623	3.980242	0.505074
C	0.794513	1.848481	3.692977
C	-0.060741	0.570911	3.693989
C	0.945129	4.168172	-0.856464
C	0.224746	3.283903	-1.888260
C	0.925069	2.440372	5.102146
C	0.982890	5.634429	-1.301696
C	5.274310	1.562720	-3.185406
C	5.470950	-1.453706	3.313499
H	-3.773632	-3.166835	3.017500
H	-6.698386	-0.021393	2.572151
H	-6.443122	2.106889	-2.109891
H	-3.385997	0.214402	-4.500608
H	-7.040077	2.628062	-0.046887
H	-7.130694	1.893642	1.557363
H	-7.412394	0.892242	0.110307
H	-4.736089	3.514707	0.476462
H	-3.448425	2.418694	1.030538
H	-4.826231	2.771831	2.099725
H	-5.872692	-2.060084	3.698814
H	-5.452739	1.554604	-4.304780
H	3.423998	4.143980	1.953472
H	5.414726	2.368410	1.123593
H	-1.529504	3.255997	3.876866
H	-2.529597	4.800409	2.224377
H	-1.400925	5.227401	0.067147
H	5.420930	-1.253097	-2.901274
H	6.096348	-3.086973	-1.387140
H	5.631141	-2.946944	1.035570
H	1.985097	3.818190	-0.792749
H	1.802325	1.567910	3.356607
H	3.808758	1.928576	-1.673556
H	4.157937	0.100815	2.667506
H	2.570446	1.412883	-3.757592
H	3.185512	-0.256291	-3.733685
H	2.095241	0.303149	-2.448913
H	4.927315	2.398781	-3.808212
H	6.134507	1.906621	-2.594826
H	5.622826	0.769301	-3.860872
H	5.195331	-1.304450	4.366321
H	5.657365	-2.527451	3.173488
H	6.410973	-0.917953	3.124050
H	2.738727	-1.652319	3.703487
H	2.228466	-1.332159	2.022349
H	3.173382	-2.787949	2.398335
H	1.384703	1.706452	5.778488
H	1.549021	3.344671	5.099916
H	-0.056096	2.706763	5.519088
H	0.373691	-0.182242	4.366101
H	-1.085240	0.782312	4.031641
H	-0.120493	0.143132	2.681615
H	0.712721	3.363079	-2.869965
H	0.236724	2.228639	-1.575755
H	-0.825130	3.591406	-1.997714
H	1.523721	5.723768	-2.253764
H	-0.027394	6.036612	-1.459298
H	1.487997	6.265881	-0.558177
H	-1.782409	-3.172338	-3.454222
H	-0.434049	-3.678713	-5.484195
H	0.821719	-1.858547	-6.634842
H	0.729308	0.462233	-5.728125
H	-0.601597	0.946787	-3.681471
H	0.144232	-2.372386	2.238481
H	1.231875	-4.470073	3.039169
H	0.100410	-6.665287	2.696919
H	-2.111558	-6.743945	1.550469
H	-3.165528	-4.645598	0.727798
C	0.884928	-2.643142	-1.015696
O	-0.245464	-3.004477	-1.192941
O	2.058158	-2.660541	-0.991990

122

INT (I')

C	-0.897806	-4.375404	-1.865906
C	-1.055097	-3.679958	-0.660537
C	-0.439795	-4.181924	0.495672
C	0.319181	-5.349261	0.446879
C	0.480006	-6.033186	-0.760301
C	-0.130313	-5.539656	-1.914704
N	-1.794154	-2.464976	-0.619254
C	-2.922626	-2.419061	0.183284
C	-3.538611	-1.160718	0.278193
C	-4.765766	-0.876476	0.843281
C	-5.399882	-1.957003	1.483420
C	-4.794059	-3.216483	1.493869
C	-3.579292	-3.468853	0.850069
C	-5.325596	0.549873	0.763566
C	-4.743218	1.255223	-0.468474
C	-3.513087	0.866379	-0.960933
O	-2.792160	-0.179246	-0.378721
C	-5.357228	2.319946	-1.152420
C	-4.728390	2.894514	-2.260346
C	-3.507845	2.426412	-2.755181
C	-2.871022	1.349948	-2.112272
Al	-1.195343	-0.863914	-1.470152
N	-1.743700	0.643682	-2.503506
C	-0.963139	1.169485	-3.569241
C	-0.734088	0.411653	-4.724897
C	0.071307	0.910324	-5.748823
C	0.650071	2.175942	-5.641742
C	0.417779	2.940647	-4.496209
C	-0.377770	2.440683	-3.467907
C	-6.858832	0.528406	0.702904
C	-4.869832	1.320406	2.021639
Au	1.002173	-0.154998	-0.225301
C	1.830908	0.836418	1.435990
N	3.190229	0.816959	1.562581
C	3.610336	1.510233	2.684574
C	2.485059	1.977854	3.286965
N	1.411458	1.558536	2.509060
C	4.072667	0.166185	0.624838
C	4.454916	-1.162479	0.880885
C	5.307748	-1.769357	-0.045794
C	5.752875	-1.078521	-1.169843
C	5.350869	0.233658	-1.395524
C	4.497512	0.889340	-0.502317
C	3.917163	-1.941798	2.068088
C	2.802049	-2.895703	1.612731
C	4.043764	2.310455	-0.781971
C	3.208708	2.374940	-2.068653
C	0.057516	1.894399	2.869318
C	-0.648839	1.010913	3.701027
C	-1.891940	1.441269	4.178151
C	-2.394982	2.692317	3.835602
C	-1.689055	3.527020	2.973187
C	-0.441913	3.146217	2.468062
C	-0.125628	-0.374004	4.030955
C	-0.827677	-1.413361	3.141957
C	0.312113	4.015678	1.479240
C	-0.122904	3.658231	0.047714
C	-0.260514	-0.725024	5.516941
C	0.159702	5.516502	1.743299
C	5.234645	3.277964	-0.836071
C	5.017397	-2.696066	2.824297
H	-3.158502	-4.472795	0.834508
H	-6.365403	-1.820665	1.966602
H	-6.324551	2.698056	-0.827362
H	-3.067936	2.864962	-3.649217
H	-7.258015	1.549463	0.674910
H	-7.273637	0.046167	1.596227
H	-7.214348	-0.011620	-0.183887
H	-5.241478	2.354145	1.990942
H	-3.776969	1.349962	2.080551
H	-5.251579	0.830000	2.928073
H	-5.298451	-4.040835	2.000096
H	-5.218164	3.722383	-2.775201
H	2.340408	2.563668	4.185471
H	4.656768	1.602532	2.945287
H	-2.473299	0.786657	4.826970
H	-3.360417	3.012602	4.228009
H	-2.111830	4.491022	2.692972
H	5.693766	0.756584	-2.288518
H	6.411446	-1.573900	-1.883899
H	5.617844	-2.802860	0.107462
H	1.382297	3.775977	1.567031
H	0.944929	-0.401558	3.782663
H	3.394788	2.632347	0.044681
H	3.471421	-1.224289	2.772161
H	2.843278	3.397479	-2.236004
H	3.802484	2.078581	-2.944379
H	2.340749	1.704576	-2.015109
H	4.882179	4.306167	-0.997710
H	5.812754	3.253925	0.097930
H	5.914997	3.023143	-1.660621
H	4.595849	-3.178956	3.716535
H	5.464873	-3.484912	2.204214
H	5.821121	-2.019564	3.145159
H	2.378239	-3.432089	2.473453
H	1.989858	-2.352121	1.109899
H	3.186632	-3.637728	0.899601
H	0.206350	-1.699486	5.714143
H	0.228639	0.026114	6.151736
H	-1.313068	-0.796907	5.823501
H	-0.449134	-2.420189	3.366919
H	-1.913789	-1.410361	3.309572
H	-0.650245	-1.207027	2.077024
H	0.445041	4.252050	-0.682212
H	0.046005	2.594133	-0.168928
H	-1.193641	3.864453	-0.094482
H	0.813405	6.080398	1.064490
H	-0.868489	5.859467	1.563759
H	0.431128	5.774023	2.775839
H	-1.181417	-0.577687	-4.805891
H	0.246746	0.302465	-6.637400
H	1.279577	2.563020	-6.443478
H	0.870902	3.928094	-4.396763
H	-0.546479	3.026852	-2.565691
H	-0.553458	-3.635295	1.430716
H	0.800141	-5.718433	1.353931
H	1.081198	-6.942046	-0.801847
H	-0.008861	-6.063326	-2.863739
H	-1.369896	-3.985618	-2.766737
C	1.075132	-1.251385	-2.006650
O	-0.006235	-1.611515	-2.652812
O	2.226595	-1.493629	-2.336150

122

TSII (I')

C	-0.821877	-2.861858	-1.929086
C	-1.036013	-2.738934	-0.546319
C	-0.026737	-2.945933	0.409599
C	1.240871	-3.295819	-0.066384
C	1.486929	-3.430511	-1.430119
C	0.463652	-3.220973	-2.348933
N	-2.335828	-2.325709	-0.083681
C	-2.670809	-1.016305	0.088727
N	-3.957231	-1.057959	0.531676
C	-4.416024	-2.364264	0.635558
C	-3.387710	-3.167641	0.245127
Au	-1.492060	0.583386	-0.403670
C	-0.335050	2.109967	-1.100963

O	-0.741528	3.196699	-1.489053
C	-4.736921	0.119286	0.815360
C	-4.661329	0.679619	2.101128
C	-5.426366	1.824939	2.346310
C	-6.220196	2.383329	1.348219
C	-6.258520	1.813625	0.078632
C	-5.512589	0.668209	-0.219723
C	-3.735939	0.128258	3.170348
C	-4.455460	-0.109524	4.503442
C	-5.497489	0.105792	-1.630062
C	-6.909864	-0.151208	-2.169926
C	-0.269979	-2.736390	1.893827
C	0.201868	-3.928229	2.735921
C	-1.900145	-2.558673	-2.952875
C	-2.133454	-3.739787	-3.904313
Al	2.299450	1.030786	-0.464091
N	2.434862	1.873099	1.207534
C	3.232092	1.281987	2.179085
C	3.785693	0.044867	1.822911
C	4.732014	-0.679099	2.521936
C	5.103674	-0.136850	3.764252
C	4.531285	1.060480	4.205249
C	3.611769	1.779496	3.437218
C	5.265709	-1.989222	1.927980
C	5.220912	-1.885152	0.396342
C	4.265690	-1.088689	-0.205705
O	3.329825	-0.379933	0.562852
C	4.152544	-0.781833	-1.569969
C	5.044487	-1.461235	-2.418345
C	5.987644	-2.330969	-1.864054
C	6.100561	-2.536613	-0.485875
C	6.694950	-2.271012	2.409764
C	4.346508	-3.143255	2.393581
N	3.220450	0.202921	-1.880173
C	2.887914	0.441041	-3.241188
C	2.879154	1.748596	-3.742453
C	2.497114	1.999051	-5.059633
C	2.130731	0.947766	-5.901765
C	2.143631	-0.359594	-5.409220
C	2.514146	-0.611524	-4.090419
C	1.622628	2.984632	1.567226
C	1.680189	4.169865	0.826588
C	0.831986	5.233416	1.132339
C	-0.071656	5.133286	2.190448
C	-0.129799	3.952745	2.935509
C	0.706049	2.883576	2.624110
C	-2.527289	1.061828	3.342322
C	-4.701691	1.037152	-2.559245
C	0.378232	-1.424702	2.364580
C	-1.554868	-1.274195	-3.723070
H	3.213004	2.728842	3.790599
H	5.840926	-0.640992	4.385821
H	6.881210	-3.190008	-0.101882
H	5.018946	-1.286423	-3.492167
H	7.053433	-3.227301	2.010281
H	6.723408	-2.349863	3.503046
H	7.385432	-1.478194	2.095737
H	4.646439	-4.087915	1.920202
H	3.301892	-2.936634	2.134549
H	4.406778	-3.255273	3.484573
H	4.828940	1.461756	5.174803
H	6.678917	-2.845641	-2.532562
H	-3.309041	-4.244714	0.171525
H	-5.418137	-2.596980	0.971994
H	2.054064	-3.459101	0.638043
H	2.488761	-3.689455	-1.775234
H	0.666288	-3.318688	-3.415539
H	-6.866249	2.274341	-0.700348
H	-6.805488	3.279291	1.558482
H	-5.388785	2.293555	3.329813
H	-2.840979	-2.376959	-2.414588
H	-1.353548	-2.637960	2.047564
H	-4.973981	-0.860298	-1.607178
H	-3.355869	-0.842827	2.823360
H	-4.638676	0.607302	-3.568736
H	-5.187262	2.020344	-2.634433
H	-3.680340	1.190218	-2.181816
H	-6.854007	-0.607471	-3.167767
H	-7.473061	-0.827376	-1.512322
H	-7.480338	0.783106	-2.264688
H	-3.764916	-0.562608	5.228123
H	-4.819885	0.831498	4.938082
H	-5.315398	-0.781739	4.380012
H	-1.820707	0.645207	4.074005
H	-1.999057	1.205061	2.389066
H	-2.848544	2.050587	3.699332
H	-0.045786	-3.763123	3.793392
H	-0.277381	-4.861404	2.410748
H	1.289684	-4.066738	2.670126
H	0.143508	-1.237089	3.421507
H	1.471136	-1.468002	2.265588
H	0.013417	-0.572403	1.772292
H	-2.356042	-1.028350	-4.433833
H	-1.428342	-0.428052	-3.032384
H	-0.617408	-1.387808	-4.285219
H	-2.952199	-3.508328	-4.599463
H	-1.238080	-3.953203	-4.504779
H	-2.396616	-4.652927	-3.353071
H	3.160080	2.572670	-3.086363
H	2.486988	3.025171	-5.428898
H	1.833475	1.145542	-6.931855
H	1.850067	-1.189706	-6.053723
H	2.498257	-1.627311	-3.698083
H	0.642262	1.950429	3.184600
H	-0.845502	3.855910	3.752747
H	-0.735729	5.965167	2.425836
H	0.875366	6.143698	0.533852
H	2.378367	4.242678	-0.007428
O	0.989468	1.820273	-1.261528

122

PC (I’)

C	-3.576243	2.816919	0.115173
C	-3.754525	1.819505	-0.858522
C	-3.360712	1.971548	-2.198463
C	-2.779336	3.191562	-2.558861
C	-2.602151	4.205650	-1.622829
C	-2.995572	4.019344	-0.300613
N	-4.315507	0.555970	-0.447994
C	-5.664396	0.244817	-0.395981
C	-5.747312	-1.042954	0.041460
N	-4.443639	-1.477021	0.239765
C	-3.547194	-0.499475	-0.059986
Au	-1.512908	-0.451130	0.029143
C	0.486919	-0.158626	0.086838
O	0.943046	1.059586	0.143936
Al	2.695178	0.357505	0.118256
N	3.320614	0.784401	1.854268
C	2.658771	1.667110	2.749902
C	-4.069140	-2.794192	0.684601
C	-3.872026	-3.005913	2.058910
C	-3.500738	-4.294452	2.459111
C	-3.333387	-5.314093	1.526141
C	-3.526287	-5.066800	0.169051
C	-3.897681	-3.797059	-0.284963
C	-3.992197	-1.886190	3.076920
C	-2.598088	-1.469909	3.574088
C	-4.030643	-3.508171	-1.769459
C	-4.857326	-4.566945	-2.507480
C	-3.495091	0.858686	-3.221700

C	-4.349984	1.291605	-4.420311
C	-3.911435	2.589159	1.577800
C	-4.781748	3.710472	2.158269
O	1.438152	-1.057060	0.061070
N	3.301951	0.846805	-1.619111
C	4.512970	0.300570	-2.026694
C	5.150337	-0.529630	-1.092668
C	6.431924	-1.042490	-1.161338
C	7.133584	-0.777387	-2.347342
C	6.525430	-0.011652	-3.348722
C	5.246787	0.533900	-3.207614
C	6.951872	-1.816781	0.058737
C	6.450456	-1.074063	1.305065
C	5.176191	-0.546354	1.257425
O	4.401608	-0.706292	0.089030
C	4.554530	0.265565	2.216941
C	5.280472	0.433330	3.412687
C	6.554471	-0.130640	3.531700
C	7.158959	-0.854617	2.497724
C	8.476463	-1.933962	0.046583
C	6.332000	-3.237055	0.045835
C	2.494456	1.539130	-2.550962
C	-4.913991	-2.258545	4.244777
C	-2.636664	-3.348537	-2.398422
C	-2.109064	0.369646	-3.666273
C	-2.619399	2.403422	2.389712
H	4.861625	1.024402	4.225450
H	8.170546	-1.236900	2.619874
H	8.146765	-1.149339	-2.486766
H	4.833158	1.167411	-3.989967
H	8.810430	-2.476799	-0.846598
H	8.821772	-2.500475	0.920503
H	8.957485	-0.947382	0.056982
H	6.650108	-3.776033	-0.857048
H	5.236035	-3.193492	0.055621
H	6.664651	-3.798073	0.929789
H	7.104569	0.019416	4.461885
H	7.078478	0.191026	-4.267043
H	-6.599900	-1.683042	0.227738
H	-6.430786	0.958359	-0.669865
H	-3.332357	-4.498814	3.516671
H	-3.041710	-6.310884	1.858845
H	-3.375927	-5.871311	-0.550716
H	-2.440861	3.339312	-3.584410
H	-2.137985	5.145929	-1.922501
H	-2.829909	4.813679	0.427203
H	-4.554313	-2.548399	-1.882967
H	-4.435251	-1.015219	2.573810
H	-4.005182	0.011529	-2.741920
H	-4.486500	1.655614	1.654631
H	-4.988757	-4.273858	-3.557991
H	-4.360662	-5.546862	-2.499926
H	-5.851531	-4.686142	-2.055931
H	-2.723672	-3.082662	-3.461152
H	-2.061362	-2.561923	-1.889553
H	-2.069097	-4.286991	-2.323221
H	-5.021957	-1.403585	4.926159
H	-5.913366	-2.544517	3.889544
H	-4.508255	-3.097148	4.827203
H	-2.677011	-0.626732	4.274368
H	-2.103493	-2.303102	4.093091
H	-1.959211	-1.163595	2.732642
H	-2.207067	-0.466451	-4.372601
H	-1.540512	1.171228	-4.155885
H	-1.519280	0.029499	-2.803137
H	-4.459495	0.458545	-5.128396
H	-5.352701	1.609068	-4.102709
H	-3.885563	2.129512	-4.958899
H	-2.855871	2.172781	3.438046
H	-2.008675	1.586989	1.979489
H	-2.006429	3.315237	2.368492
H	-5.048197	3.480643	3.199006
H	-4.250989	4.672552	2.157542
H	-5.709809	3.835255	1.583968
C	1.913471	2.767033	-2.196089
C	1.067097	3.435460	-3.078571
C	0.796282	2.904162	-4.340738
C	1.369712	1.682370	-4.703004
C	2.202666	1.002229	-3.817505
H	2.123606	3.187422	-1.212470
H	0.618624	4.382467	-2.776038
H	0.141862	3.433381	-5.034300
H	1.154566	1.244597	-5.678997
H	2.624488	0.036299	-4.095441
C	1.397861	1.332862	3.262424
C	0.719678	2.213702	4.104594
C	1.295593	3.434366	4.460352
C	2.555938	3.771444	3.958575
C	3.230645	2.897923	3.106963
H	0.952477	0.376826	2.987783
H	-0.265043	1.941703	4.486956
H	0.766330	4.120817	5.122040
H	3.013264	4.725842	4.223415
H	4.206697	3.165708	2.700280

122

PC_CO (I')

C	-0.819612	3.670440	0.670008
C	-1.636507	3.316873	-0.416228
C	-1.392810	3.741552	-1.733872
C	-0.284363	4.568072	-1.945888
C	0.540306	4.944065	-0.888708
C	0.276938	4.497274	0.402871
N	-2.767718	2.460886	-0.178722
C	-2.696488	1.096075	-0.184028
N	-3.988261	0.696979	0.018828
C	-4.840397	1.787006	0.139033
C	-4.069592	2.900484	0.018403
Au	-1.099251	-0.001833	-0.491067
O	0.501582	-1.105908	-0.915609
C	-1.650164	-3.038285	0.552438
C	-4.411789	-0.676301	0.086582
C	-4.605294	-1.379761	-1.113270
C	-5.029590	-2.710310	-1.013908
C	-5.249955	-3.300726	0.225822
C	-5.043824	-2.576774	1.398390
C	-4.617317	-1.245898	1.356836
C	-4.368750	-0.755879	-2.476606
C	-5.666922	-0.698666	-3.294870
C	-4.318257	-0.471200	2.629098
C	-5.291343	-0.786669	3.769310
C	-2.245579	3.275040	-2.900182
C	-2.708363	4.436271	-3.788464
C	-1.071194	3.160451	2.076862
C	-1.235932	4.313618	3.076172
Al	2.014872	-1.033685	-0.173454
N	3.436673	-1.623085	-1.280111
C	4.430951	-0.677758	-1.484735
C	4.286148	0.518309	-0.759428
C	5.205039	1.539027	-0.622015
C	6.348884	1.441439	-1.428987
C	6.484629	0.344888	-2.288669
C	5.566374	-0.707866	-2.323886
C	4.901310	2.622970	0.426002
C	4.276773	1.905705	1.635052
C	3.377645	0.891111	1.374847
O	3.100974	0.578466	0.019551
C	2.807338	-0.010677	2.289193
C	3.119835	0.252612	3.638864
C	3.967578	1.316455	3.959683

C	4.560149	2.134013	2.990319
C	6.165275	3.388809	0.819335
C	3.866746	3.616251	-0.157688
N	2.106410	-1.063584	1.718500
C	1.400347	-2.020531	2.464303
C	1.388625	-3.346963	1.993906
C	0.655940	-4.333492	2.648042
C	-0.072672	-4.024316	3.798587
C	-0.071068	-2.709927	4.270442
C	0.647707	-1.714749	3.611769
C	3.495830	-2.944163	-1.758705
C	4.691350	-3.679404	-1.875338
C	4.664341	-5.010975	-2.287022
C	3.455652	-5.647159	-2.579506
C	2.263808	-4.930957	-2.443643
C	2.278714	-3.596317	-2.046237
C	-3.257023	-1.493206	-3.236207
C	-2.863149	-0.713216	3.061394
C	-1.492583	2.209652	-3.712811
C	0.041747	2.195193	2.507968
H	5.737638	-1.553532	-2.985646
H	7.127157	2.200855	-1.386723
H	5.251192	2.919951	3.288825
H	2.742334	-0.395369	4.426470
H	5.928620	4.155281	1.568097
H	6.585372	3.903855	-0.053700
H	6.931193	2.720351	1.233025
H	3.624825	4.383325	0.590689
H	2.937057	3.111159	-0.443105
H	4.281954	4.109823	-1.046901
H	7.358237	0.290593	-2.939709
H	4.200067	1.493702	5.010673
H	-4.320603	3.952537	0.050962
H	-5.903827	1.666107	0.299651
H	-0.056790	4.911743	-2.955181
H	1.403415	5.583844	-1.075809
H	0.940323	4.785509	1.218587
H	-5.208915	-3.058387	2.361435
H	-5.578993	-4.339083	0.281689
H	-5.185871	-3.290194	-1.924007
H	-2.011409	2.591938	2.069079
H	-3.147530	2.798208	-2.491890
H	-4.422055	0.599877	2.402316
H	-4.027340	0.276810	-2.324399
H	-2.622969	-0.108838	3.947968
H	-2.702087	-1.770964	3.307712
H	-2.155506	-0.460339	2.261191
H	-5.095013	-0.119860	4.619748
H	-6.336476	-0.650788	3.459101
H	-5.173942	-1.817689	4.130929
H	-5.486021	-0.203966	-4.259339
H	-6.051142	-1.707709	-3.501205
H	-6.450109	-0.141445	-2.762851
H	-3.066547	-1.001069	-4.200042
H	-2.323256	-1.491760	-2.657180
H	-3.536808	-2.536913	-3.436759
H	-3.375898	4.063441	-4.577457
H	-3.251730	5.193126	-3.206509
H	-1.859928	4.931804	-4.280543
H	-2.128621	1.826153	-4.523179
H	-0.583255	2.633838	-4.161992
H	-1.195884	1.367478	-3.071717
H	-0.156934	1.809748	3.517576
H	0.100923	1.340384	1.820072
H	1.022692	2.690413	2.521488
H	-1.468117	3.918253	4.074713
H	-0.314177	4.906508	3.158370
H	-2.047558	4.989974	2.775125
H	1.973532	-3.604929	1.106319
H	0.662546	-5.352427	2.258864
H	-0.639640	-4.797109	4.317558
H	-0.653128	-2.448366	5.155356
H	0.596563	-0.685534	3.962659
H	1.347117	-3.030885	-1.952969
H	1.308606	-5.412204	-2.659482
H	3.443266	-6.688313	-2.902577
H	5.603131	-5.561566	-2.365700
H	5.640713	-3.218895	-1.608404
O	-1.614899	-3.766723	-0.322810

95

IPrAuB(*o*-tol)$_2$ (Complex III)

C	1.802054	-2.995525	1.019870
C	2.255134	-2.374733	-0.155631
C	2.248288	-3.007946	-1.411705
C	1.799103	-4.331131	-1.460595
C	1.365666	-4.982858	-0.307964
C	1.359506	-4.319695	0.914344
N	2.726793	-1.016351	-0.079546
C	1.898495	0.065611	-0.091141
N	2.746365	1.126228	0.015730
C	4.071982	0.719125	0.092361
C	4.059246	-0.641322	0.033383
Au	-0.207523	0.064373	-0.112751
B	-2.281581	-0.011047	0.007105
C	-2.931194	-1.277981	0.665866
C	-3.791229	-1.051296	1.761884
C	-4.277498	-2.092537	2.552420
C	-3.946617	-3.408815	2.229772
C	-3.134724	-3.662901	1.121742
C	-2.618719	-2.625083	0.338784
C	-1.759869	-2.945944	-0.851287
C	2.291943	2.490372	0.079485
C	2.100199	3.067664	1.347028
C	1.646856	4.390153	1.386977
C	1.391909	5.095421	0.213551
C	1.573049	4.488571	-1.025334
C	2.024805	3.167289	-1.121484
C	-3.243338	1.128089	-0.472345
C	-2.994230	2.525044	-0.367419
C	-3.879590	3.427965	-0.967692
C	-4.997370	2.993396	-1.683864
C	-5.262683	1.627651	-1.786225
C	-4.404706	0.720231	-1.166484
C	-1.820697	3.054901	0.406647
H	4.889010	1.423681	0.181368
H	4.863264	-1.365828	0.059102
H	-2.891098	-4.695034	0.858392
H	-4.327468	-4.237197	2.829396
H	-4.924912	-1.879288	3.404747
H	-4.069093	-0.022987	2.005042
H	-6.139748	1.274051	-2.330664
H	-5.662792	3.721926	-2.150273
H	-3.685913	4.499333	-0.874578
H	-4.625783	-0.347713	-1.225082
H	-2.269201	-2.695268	-1.793855
H	-0.838337	-2.337747	-0.829366
H	-1.484640	-4.007340	-0.878123
H	-1.666869	4.125734	0.223074
H	-0.901063	2.506941	0.139977
H	-1.954841	2.909103	1.488970
C	2.288190	2.270498	2.625874
H	1.477123	4.870341	2.350706
H	1.035189	6.124812	0.266176
H	1.348423	5.044431	-1.935931
C	2.153209	2.497403	-2.476859
C	2.618763	-2.256522	-2.678489
H	1.774739	-4.855680	-2.415326
H	1.016151	-6.014314	-0.368261
H	0.997017	-4.832974	1.805365

C	1.761978	-2.284269	2.359983
C	0.761400	2.269088	-3.087384
C	3.063321	3.287048	-3.426709
H	2.609798	1.509595	-2.325401
C	0.921568	1.807566	3.158356
H	2.866967	1.368833	2.381203
C	3.074312	3.038838	3.693985
C	1.347792	-1.673560	-3.321682
H	3.261756	-1.411113	-2.394224
C	3.403665	-3.108895	-3.680215
C	0.323449	-2.191713	2.889819
C	2.697399	-2.956437	3.374888
H	2.117944	-1.256192	2.209775
H	0.847120	1.737557	-4.045358
H	0.133144	1.670393	-2.410727
H	0.251531	3.225805	-3.269506
H	3.173387	2.749151	-4.378458
H	2.645667	4.278373	-3.651819
H	4.063077	3.431227	-2.994535
H	1.050916	1.180071	4.051530
H	0.300654	2.672762	3.431732
H	0.378381	1.225859	2.398631
H	3.248458	2.394953	4.566853
H	4.048770	3.372236	3.311871
H	2.525573	3.924443	4.043246
H	3.725634	-2.487688	-4.527075
H	4.296815	-3.551938	-3.219034
H	2.790767	-3.924107	-4.088955
H	1.605667	-1.074599	-4.206632
H	0.673069	-2.482592	-3.636285
H	0.800701	-1.034355	-2.614087
H	0.305097	-1.630620	3.834588
H	-0.326367	-1.676982	2.168572
H	-0.100494	-3.188186	3.076840

H 2.685529 -2.405045
4.325432optim_NHCreAlAuB.xyz

H	2.382184	-3.989074	3.581350
H	3.732319	-2.986509	3.006772

98

RC (III)

C	3.655329	2.286566	1.341034
C	2.517420	2.219943	0.505703
C	1.754260	3.407366	0.334918
C	2.125095	4.568318	1.022608
C	3.223731	4.590731	1.883846
C	3.997699	3.441014	2.043986
B	2.138334	0.802036	-0.044176
C	3.294285	-0.061620	-0.656209
C	4.084815	0.559984	-1.647359
C	5.023483	-0.144572	-2.400045
C	5.229069	-1.499959	-2.139708
C	4.492436	-2.128001	-1.133075
C	3.528125	-1.437777	-0.390313
C	2.778260	-2.150386	0.698148
C	0.583954	3.450116	-0.604839
Au	0.195447	0.058510	-0.067517
C	-1.751398	-0.732768	-0.256087
N	-2.168627	-2.009334	-0.022924
C	-3.507685	-2.179923	-0.348497
C	-3.949416	-0.974799	-0.802439
N	-2.864745	-0.110766	-0.736588
C	-1.300467	-3.053438	0.456597
C	-0.572595	-3.800523	-0.484665
C	0.264623	-4.808172	0.007593
C	0.372109	-5.046614	1.374071
C	-0.351593	-4.277985	2.282464
C	-1.207500	-3.262594	1.843400
C	-0.631663	-3.512313	-1.973913
C	-0.998735	-4.761022	-2.786374
C	-1.937962	-2.373672	2.833540
C	-2.575956	-3.157781	3.985549
C	-2.883430	1.265979	-1.156568
C	-2.555647	1.560731	-2.490903
C	-2.575208	2.906640	-2.872474
C	-2.901874	3.905901	-1.959941
C	-3.205595	3.581598	-0.640847
C	-3.200167	2.251529	-0.207283
C	-2.128902	0.487268	-3.475913
C	-2.938959	0.536428	-4.777895
C	-3.450269	1.912419	1.250321
C	-4.736156	2.550737	1.787648
C	-0.619635	0.589447	-3.748627
C	-2.227088	2.306259	2.092648
C	-0.981663	-1.289364	3.356997
C	0.692132	-2.897520	-2.453831
H	-4.919718	-0.656760	-1.162220
H	-4.013904	-3.129342	-0.228998
H	4.668584	-3.184794	-0.917991
H	5.967818	-2.065967	-2.709640
H	5.603063	0.363629	-3.172680
H	3.941248	1.626273	-1.837284
H	4.870413	3.448365	2.699131
H	3.479957	5.507910	2.416718
H	1.534699	5.477087	0.882894
H	4.272456	1.392302	1.450419
H	3.102660	-1.814190	1.693736
H	1.700530	-1.919010	0.637289
H	2.914273	-3.237290	0.638631
H	-0.029145	4.346682	-0.449627
H	-0.047197	2.553903	-0.479101
H	0.914447	3.441609	-1.654482
H	-2.319263	3.176234	-3.897404
H	-2.907088	4.949394	-2.277113
H	-3.436877	4.375645	0.069372
H	-0.243837	-4.464912	3.350781
H	1.035077	-5.832763	1.737367
H	0.851046	-5.404546	-0.691786
H	-3.565427	0.822340	1.329406
H	-2.312301	-0.491634	-3.011787
H	-2.749628	-1.864751	2.294301
H	-1.419566	-2.765564	-2.144154
H	-2.374882	2.021250	3.143535
H	-1.318819	1.809473	1.724442
H	-2.057296	3.391584	2.051584
H	-4.905073	2.238941	2.827574
H	-4.677378	3.647968	1.778223
H	-5.610194	2.252934	1.192360
H	-0.295941	-0.218954	-4.418874
H	-0.373653	1.549842	-4.223918
H	-0.047233	0.513265	-2.811795
H	-2.639417	-0.288641	-5.438694
H	-4.016476	0.448348	-4.582598
H	-2.768946	1.475788	-5.322121
H	-3.164567	-2.479629	4.618312
H	-3.242680	-3.947229	3.612985
H	-1.817222	-3.627240	4.626785
H	-1.510193	-0.600500	4.030851
H	-0.147944	-1.745872	3.909222
H	-0.558929	-0.708869	2.523370
H	0.635961	-2.662288	-3.525839
H	0.917173	-1.970540	-1.907562
H	1.529662	-3.592017	-2.298586
H	-1.089836	-4.504764	-3.850977
H	-0.227649	-5.538967	-2.696712
H	-1.953215	-5.190488	-2.452314
O	2.317020	0.127034	3.529977
C	1.601863	1.053561	3.556186
O	0.879881	1.971697	3.609756

98

TSI (III)

C	0.679237	3.799423	0.330472
C	0.109068	3.154342	-0.780820
C	0.705445	3.130164	-2.052508
C	1.918535	3.811374	-2.201735
C	2.500388	4.477659	-1.127642
C	1.890991	4.466305	0.124536
N	-1.150869	2.475296	-0.614427
C	-1.271584	1.183950	-0.196603
N	-2.609648	0.939503	-0.256832
C	-3.309645	2.050491	-0.703123
C	-2.385133	3.024327	-0.927683
Au	0.254517	-0.121758	0.378235
B	1.749391	-1.638576	0.313622
C	1.130623	-2.924658	-0.351346
C	1.774236	-3.321019	-1.546831
C	1.302437	-4.363287	-2.342439
C	0.173659	-5.073797	-1.937182
C	-0.474541	-4.711128	-0.756446
C	-0.035061	-3.643517	0.035512
C	-0.822541	-3.295639	1.264027
C	-3.211913	-0.336371	0.031824
C	-3.305688	-1.271578	-1.012035
C	-3.900832	-2.502280	-0.713886
C	-4.381800	-2.773669	0.563424
C	-4.270408	-1.824222	1.576241
C	-3.681461	-0.578923	1.333765
C	-2.803379	-0.980526	-2.414666
C	-1.719915	-1.977162	-2.844010
C	-3.510510	0.436277	2.449912
C	-2.308511	0.061440	3.331302
C	0.105467	2.347414	-3.208163
C	0.835371	1.005185	-3.379358
C	-0.002988	3.794625	1.686701
C	0.985557	3.744785	2.854575
C	3.316198	-1.471429	0.320316
C	4.063080	-2.625566	0.641967
C	5.449409	-2.609410	0.763001
C	6.138094	-1.417411	0.534696
C	5.427558	-0.267589	0.194461
C	4.029092	-0.267193	0.088772
C	3.346794	1.015248	-0.297493
C	-3.967372	-0.948347	-3.416511
C	-4.782220	0.607705	3.289247
C	0.093644	3.145470	-4.517337
C	-0.946332	5.002976	1.815326
H	-2.488187	4.040536	-1.285588
H	-4.385785	2.043500	-0.818252
H	-1.363758	-5.261751	-0.441626
H	-0.203886	-5.904122	-2.535992
H	1.823474	-4.627370	-3.263970
H	2.671698	-2.784486	-1.858395
H	5.988911	-3.518868	1.031194
H	7.225274	-1.381183	0.620996
H	5.968695	0.663224	0.011132
H	3.525138	-3.557943	0.819978
H	-0.251258	-3.487243	2.180000
H	-1.064105	-2.220012	1.273535
H	-1.761340	-3.860174	1.298453
H	4.053685	1.854108	-0.293540
H	2.520010	1.256676	0.386030
H	2.910754	0.951540	-1.303598
H	2.418951	3.811254	-3.169890
H	3.447588	5.000751	-1.264028
H	2.370610	4.975862	0.959130
H	-4.640505	-2.057402	2.574314
H	-4.841992	-3.739317	0.775768
H	-3.984509	-3.258048	-1.495200
H	-0.612612	2.881212	1.744520
H	-0.940522	2.120735	-2.958248
H	-3.289193	1.409920	1.989335
H	-2.343632	0.017091	-2.412205
H	0.433917	3.631181	3.797548
H	1.667908	2.892166	2.758978
H	1.575619	4.668852	2.930305
H	-1.470058	4.977226	2.781122
H	-0.376150	5.941617	1.760165
H	-1.700644	5.017527	1.017565
H	0.374700	0.418156	-4.185984
H	1.892276	1.167767	-3.634686
H	0.794329	0.409894	-2.455895
H	-0.425806	2.574833	-5.299092
H	-0.419839	4.108478	-4.394047
H	1.111388	3.346614	-4.879014
H	-4.638907	1.410186	4.025555
H	-5.645800	0.865628	2.661375
H	-5.027160	-0.307590	3.845428
H	-2.162959	0.810591	4.122023
H	-2.465320	-0.916862	3.807121
H	-1.384859	0.005563	2.738958
H	-1.347381	-1.720291	-3.845265
H	-0.873384	-1.970274	-2.146654
H	-2.110332	-3.002907	-2.881793
H	-3.598327	-0.691993	-4.419332
H	-4.460854	-1.928468	-3.479358
H	-4.724992	-0.206842	-3.127312
C	1.451008	-0.768896	2.376628
O	1.549037	-1.968471	2.577255
O	1.618868	0.320094	2.869632

98

INT (III)

C	3.571167	-1.090331	-0.364785
C	3.171582	-0.549555	0.874684
C	3.016352	-1.432917	1.970699
C	3.248146	-2.802158	1.778364
C	3.610807	-3.315999	0.535893
C	3.773144	-2.453697	-0.548035
B	2.990272	1.009485	0.924971
O	1.975238	1.647330	1.595616
C	0.706332	1.151653	2.000342
O	0.345833	1.493180	3.109485
C	2.644841	-0.960867	3.352628
C	4.039568	1.945041	0.218969
C	3.698060	3.147553	-0.451242
C	4.717887	3.936908	-0.996596
C	6.061787	3.577280	-0.880717
C	6.408211	2.394307	-0.228639
C	5.399133	1.588693	0.297505
C	2.266664	3.587874	-0.619124
Au	-0.454887	0.189711	0.631562
C	-1.842499	-0.635597	-0.619842
H	4.451272	4.854709	-1.525407
H	6.834622	4.217866	-1.308541
H	7.454130	2.098182	-0.138159
H	5.666943	0.654333	0.794484
H	4.063298	-2.840834	-1.524850
H	3.777459	-4.387803	0.417986
H	3.148351	-3.477423	2.631158
H	3.725792	-0.410500	-1.204360
H	1.623056	2.755200	-0.931652
H	1.852146	3.963540	0.326763
H	2.191212	4.385421	-1.369466
H	3.101621	-1.606131	4.114606
H	1.555607	-0.996803	3.502046
H	2.954923	0.073560	3.539393
N	-1.821536	-1.767494	-1.374139
C	-3.034818	-1.964180	-2.023018
C	-3.836476	-0.923643	-1.670584

N	-3.097309	-0.129436	-0.808351
C	-3.531673	1.137324	-0.271277
H	-4.849538	-0.675341	-1.957411
H	-3.204547	-2.813217	-2.672030
C	-0.680458	-2.626809	-1.570087
C	-0.478706	-3.698924	-0.687037
C	0.583320	-4.565720	-0.966418
C	1.397938	-4.365719	-2.074932
C	1.191513	-3.274442	-2.914216
C	0.149308	-2.373409	-2.677482
C	-1.326504	-3.890372	0.555494
H	0.777772	-5.405238	-0.299318
H	2.218399	-5.055533	-2.275272
H	1.858207	-3.113462	-3.760945
C	-0.021257	-1.130557	-3.533523
C	-3.118895	2.298544	-0.957046
C	-3.543902	3.532725	-0.457071
C	-4.338791	3.606694	0.682519
C	-4.715941	2.443711	1.344272
C	-4.323943	1.176813	0.889817
C	-2.269040	2.227843	-2.214537
H	-3.239937	4.449250	-0.961065
H	-4.654581	4.577745	1.065278
H	-5.322722	2.506758	2.249017
C	-4.818783	-0.023966	1.687013
C	-3.794210	-1.128815	1.970613
H	-5.096032	0.399699	2.664786
C	-6.106612	-0.608101	1.082422
C	-1.233323	3.353132	-2.295089
H	-1.706800	1.285219	-2.185836
C	-3.159727	2.206270	-3.467953
H	-0.552756	3.173250	-3.138321
H	-1.702082	4.333535	-2.456841
H	-0.634082	3.404059	-1.377409
H	-2.543643	2.112532	-4.373530
H	-3.866159	1.366119	-3.446730
H	-3.740708	3.136650	-3.543488
H	-6.527792	-1.367391	1.756283
H	-6.863488	0.171820	0.925638
H	-5.907156	-1.093551	0.117438
H	-4.157925	-1.755435	2.796274
H	-3.649892	-1.785519	1.103987
H	-2.816423	-0.717285	2.253542
C	-0.542115	-3.436876	1.797198
H	-2.204499	-3.237106	0.467367
C	-1.838295	-5.327384	0.707262
H	-1.050507	-0.766238	-3.402483
C	0.928138	-0.027583	-3.033326
C	0.177536	-1.393420	-5.029599
H	-2.501880	-5.398102	1.579934
H	-2.400673	-5.647803	-0.180291
H	-1.013861	-6.037273	0.861427
H	-1.166561	-3.523819	2.697289
H	0.358847	-4.050591	1.935863
H	-0.220955	-2.389653	1.695915
H	-0.047520	-0.482811	-5.601241
H	1.214600	-1.674830	-5.258230
H	-0.480638	-2.196528	-5.387650
H	0.772638	0.901187	-3.600400
H	0.765474	0.182057	-1.966174
H	1.975106	-0.337216	-3.158505

98

TSII (III)

C	1.518830	2.934080	-2.158466
C	1.607632	2.550415	-0.805326
C	0.778422	3.106976	0.193272
C	-0.143692	4.083404	-0.192893
C	-0.245107	4.493224	-1.516872
C	0.576212	3.921281	-2.480112
N	2.564336	1.537923	-0.408910
C	3.932047	1.731444	-0.277943
C	4.462080	0.537197	0.103525
N	3.400998	-0.350127	0.224103
C	2.221273	0.252800	-0.098334
Au	0.307369	-0.438975	-0.005448
C	-1.659870	-0.851600	0.229315
O	-2.243942	-1.135566	1.296196
B	-3.804516	-0.461481	-0.201834
C	-4.967487	-1.443036	-0.560567
C	-6.257750	-0.887684	-0.682481
C	-7.362471	-1.647154	-1.060104
C	-7.194205	-3.008951	-1.309862
C	-5.929447	-3.585494	-1.187696
C	-4.806612	-2.828972	-0.829078
C	3.517382	-1.735309	0.619729
C	3.403280	-2.717988	-0.383719
C	3.520366	-4.059737	-0.005517
C	3.742894	-4.414159	1.318938
C	3.843447	-3.423522	2.289593
C	3.730092	-2.062548	1.974981
C	-3.473431	-3.520461	-0.725147
C	3.165758	-2.384553	-1.843316
C	4.344315	-2.836643	-2.717181
C	3.857662	-1.091390	3.143773
C	5.304826	-0.604263	3.330695
C	0.881099	2.715603	1.655614
C	1.794956	3.698560	2.405212
C	2.346196	2.398951	-3.322791
C	3.655872	3.186233	-3.502865
O	-2.498299	-0.641388	-0.815313
C	-4.054401	0.961785	0.420998
C	-4.739746	1.189342	1.638612
C	-4.915828	2.504328	2.085733
C	-4.447854	3.594859	1.349539
C	-3.765093	3.377966	0.154294
C	-3.560887	2.071154	-0.288773
C	-5.229489	0.041979	2.481534
C	1.836574	-2.970148	-2.338998
C	2.872175	0.080703	3.167500
C	-0.484926	2.586380	2.339333
C	2.600367	0.889112	-3.345344
H	-5.425253	2.676025	3.036728
H	-4.607129	4.609389	1.718563
H	-3.380430	4.218965	-0.424179
H	-2.995904	1.901083	-1.207277
H	-8.345434	-1.184017	-1.153625
H	-8.046828	-3.626050	-1.597997
H	-5.806027	-4.653594	-1.378700
H	-6.386532	0.177475	-0.480323
H	-6.085033	-0.465520	2.013096
H	-4.439099	-0.711560	2.600331
H	-5.542145	0.388984	3.474681
H	-3.591603	-4.605397	-0.838343
H	-2.992328	-3.316058	0.239732
H	-2.783355	-3.164327	-1.502386
H	5.485100	0.240626	0.290256
H	4.399917	2.688723	-0.463004
H	3.430126	-4.835502	-0.766322
H	3.826691	-5.464421	1.600130
H	3.999309	-3.702588	3.332886
H	-0.803352	4.515951	0.557443
H	-0.976532	5.250308	-1.802526
H	0.482825	4.228059	-3.523187
H	3.623920	-1.708242	4.025281
H	3.089062	-1.297366	-1.935419
H	1.345974	1.725794	1.703730
H	1.730113	2.620585	-4.208015
H	4.176904	-2.536429	-3.760975
H	5.287486	-2.387213	-2.377121

H	4.463227	-3.929146	-2.695764
H	1.663514	-2.683481	-3.385668
H	1.834585	-4.067728	-2.282587
H	0.998561	-2.591761	-1.735270
H	5.394266	-0.052291	4.276860
H	6.008965	-1.446294	3.354795
H	5.606178	0.073604	2.521641
H	2.839982	0.506884	4.179493
H	3.176580	0.886632	2.489129
H	1.856658	-0.237712	2.897922
H	-0.350269	2.180728	3.351369
H	-0.994621	3.554528	2.438840
H	-1.146606	1.908591	1.785075
H	1.907390	3.390727	3.454212
H	2.795053	3.742874	1.951756
H	1.370676	4.712916	2.388547
H	2.898072	0.585124	-4.358171
H	3.417069	0.603928	-2.671151
H	1.702497	0.322413	-3.067002
H	4.143545	2.894224	-4.443567
H	3.468835	4.267600	-3.532788
H	4.360941	2.982179	-2.686219

98

PC (III)

C	-2.216171177	-2.933663905	-0.732276376
C	-2.517462536	-2.275949258	0.471693806
C	-2.223191127	-2.810169709	1.737413318
C	-1.613175617	-4.068618618	1.773913498
C	-1.308548092	-4.751210676	0.599250914
C	-1.605002339	-4.188699468	-0.638835652
N	-3.110540662	-0.963882623	0.406700594
C	-2.373245072	0.175958449	0.315566062
N	-3.293981254	1.175029796	0.282526592
C	-4.584872955	0.671284322	0.354087159
C	-4.468261065	-0.683879083	0.431240689
Au	-0.344142067	0.280138796	0.192512830
C	1.662042438	0.231938244	0.010091752
O	2.550026673	0.116132951	0.962415415
B	3.628215349	0.002278397	-0.206674765
O	2.330010559	0.176071360	-1.110956595
C	-2.943878585	2.569652475	0.192138242
C	-2.673069765	3.265030819	1.381909907
C	-2.316467977	4.613230934	1.265935280
C	-2.230057612	5.226402065	0.019304838
C	-2.492820473	4.503781868	-1.141869897
C	-2.854194929	3.153690506	-1.082884161
C	-2.700101101	2.590002534	2.741233213
C	-1.272920555	2.452251750	3.294647648
C	-3.061168609	2.349745208	-2.354135336
C	-1.702778896	2.023091952	-2.996794158
C	-2.477207080	-2.037816212	3.019345065
C	-1.147470891	-1.534745758	3.603849066
C	-2.485091105	-2.312537216	-2.090400830
C	-1.164362369	-1.986956193	-2.804141819
C	4.337533267	-1.434442064	-0.250805540
C	5.718497358	-1.459883165	0.019246736
C	6.461044253	-2.640379619	0.057119917
C	5.820616288	-3.853284492	-0.185326465
C	4.451805543	-3.859458514	-0.453328863
C	3.694649905	-2.679294951	-0.486960567
C	2.219669190	-2.803326390	-0.778497283
C	4.564612313	1.287015930	-0.285842139
C	4.787187821	2.075136777	0.852987988
C	5.629388803	3.189554322	0.826478361
C	6.264414724	3.540828076	-0.364008747
C	6.049153201	2.773546063	-1.510772594
C	5.211672539	1.651833294	-1.490190084
C	5.006380814	0.848084193	-2.748646840
C	-3.618135735	3.319819561	3.730052599
C	-3.993199693	3.052145849	-3.348711725
C	-3.267633036	-2.858032080	4.046200207
C	-3.385100756	-3.205912920	-2.954758081
H	-5.214940761	-1.464278374	0.500938882
H	-5.453724067	1.316463773	0.342789259
H	6.543917612	3.049066376	-2.445209144
H	6.925373398	4.408256933	-0.403520257
H	5.787183604	3.780318246	1.730453115
H	4.287213536	1.806974607	1.785562029
H	7.530959791	-2.609950065	0.268811671
H	6.378816059	-4.790799804	-0.167892173
H	3.948334125	-4.810376332	-0.643470726
H	6.228438745	-0.512182100	0.201277370
H	5.291369279	-0.203301850	-2.595747342
H	3.947807986	0.845727631	-3.045259276
H	5.600031797	1.251006143	-3.579090122
H	1.946668691	-3.853753804	-0.941481630
H	1.930985752	-2.227584370	-1.666491125
H	1.604082262	-2.426473474	0.051140654
H	-1.359920507	-4.513461171	2.736451002
H	-0.825800716	-5.727650307	0.649827954
H	-1.346864766	-4.726232041	-1.551625305
H	-2.403219003	4.992700309	-2.111856656
H	-1.944907718	6.276735857	-0.049613433
H	-2.091837213	5.186732351	2.165532686
H	-3.015361848	-1.362970303	-1.933451814
H	-3.082320126	-1.154508695	2.771192627
H	-3.534405051	1.396067629	-2.080429357
H	-3.099872894	1.574782318	2.610307266
H	-1.361237707	-1.494104816	-3.766172415
H	-0.543235185	-1.317699673	-2.192135049
H	-0.586962610	-2.901303395	-2.999602808
H	-3.603442763	-2.711191987	-3.911181260
H	-2.897639705	-4.165130802	-3.178179469
H	-4.337876022	-3.419774371	-2.451444768
H	-1.328476357	-0.926098139	4.500648092
H	-0.501660702	-2.377987786	3.886124157
H	-0.603297244	-0.922061043	2.871097743
H	-3.478130817	-2.247224615	4.934696653
H	-4.223948725	-3.203308962	3.630485631
H	-2.703050764	-3.740826726	4.376417394
H	-4.169149556	2.404931978	-4.218656070
H	-4.963787616	3.286552502	-2.891051053
H	-3.556354029	3.989891319	-3.718392051
H	-1.841299670	1.394698027	-3.887606776
H	-1.187319850	2.944582622	-3.302232194
H	-1.049698932	1.487962008	-2.292350235
H	-1.283953490	1.914753901	4.252869447
H	-0.632816080	1.897889846	2.593267768
H	-0.820211630	3.439839405	3.460559636
H	-3.652225027	2.776456769	4.684242003
H	-3.256144884	4.336086993	3.938410587
H	-4.642374665	3.398440017	3.340912871

98

TS_CO (III)

C	2.274771	1.798118	-1.371711
C	2.960275	0.596041	-1.604904
C	3.773534	0.508520	-2.757509
C	3.862863	1.600293	-3.629606
C	3.177696	2.791153	-3.373220
C	2.379932	2.892380	-2.234229
B	2.765741	-0.637882	-0.600628
O	1.594809	-1.231650	-0.433551
Au	-0.589366	-0.113551	-0.874458
C	0.163688	-0.392306	-2.665575
O	0.645577	-0.665156	-3.665733
C	4.510642	-0.768968	-3.070262
C	4.034689	-1.058595	0.286733

C	4.279210	-2.370434	0.761838
C	5.338473	-2.596604	1.649478
C	6.157093	-1.553913	2.091111
C	5.932039	-0.259168	1.624596
C	4.888062	-0.031704	0.725217
C	3.424839	-3.529448	0.319589
C	-1.730384	0.232542	0.754166
H	5.522761	-3.612672	2.008392
H	6.969533	-1.756751	2.791225
H	6.567448	0.564843	1.954839
H	4.713862	0.982215	0.355903
H	1.843636	3.817531	-2.017508
H	3.269337	3.632797	-4.061807
H	4.482668	1.517798	-4.526072
H	1.648536	1.883129	-0.480782
H	2.363371	-3.308826	0.483937
H	3.534004	-3.705341	-0.761750
H	3.704111	-4.451757	0.846281
H	5.138043	-0.663794	-3.964818
H	3.804482	-1.595576	-3.244383
H	5.147370	-1.073294	-2.227087
N	-2.898279	-0.387127	1.085245
C	-3.396929	0.094756	2.286669
C	-2.520811	1.042358	2.719013
N	-1.507149	1.106232	1.774469
C	-0.320810	1.916647	1.872471
H	-2.519190	1.666648	3.602807
H	-4.315666	-0.280966	2.717643
C	-3.473786	-1.453559	0.308469
C	-0.296179	3.153342	1.206771
C	0.888219	3.894142	1.282172
C	1.991566	3.409447	1.979970
C	1.929538	2.183131	2.635897
C	0.764413	1.408638	2.607959
C	-1.488337	3.677676	0.427217
H	0.951492	4.856580	0.773687
H	2.912880	3.991931	2.007198
H	2.802751	1.813262	3.173564
C	0.688411	0.101450	3.377332
C	-2.974432	-2.753124	0.501450
C	-3.518228	-3.768356	-0.294189
C	-4.513271	-3.492601	-1.227279
C	-4.992832	-2.193934	-1.384892
C	-4.483154	-1.140886	-0.618285
C	-1.905442	-3.076672	1.530360
H	-3.154490	-4.789735	-0.178643
H	-4.920869	-4.297948	-1.839298
H	-5.770569	-1.993869	-2.121767
C	-4.963399	0.283352	-0.833045
C	-1.170079	3.791488	-1.069585
H	-2.307161	2.952568	0.531302
C	-1.979710	5.015151	0.998158
H	-0.288128	-0.358521	3.171740
C	1.760268	-0.900130	2.934030
C	0.755154	0.373804	4.888897
H	-4.634298	0.884650	0.026376
C	-4.306391	0.880563	-2.088387
C	-6.492105	0.384607	-0.901485
C	-0.626410	-3.615686	0.875019
H	-1.635763	-2.146716	2.049418
C	-2.446630	-4.049489	2.589285
H	-6.794419	1.438279	-0.973149
H	-6.893199	-0.133157	-1.783603
H	-6.963439	-0.049599	-0.009414
H	-4.611594	1.928497	-2.218597
H	-3.210959	0.840595	-2.012519
H	-4.608020	0.320914	-2.985490
H	-2.061454	4.115490	-1.624565
H	-0.373075	4.526429	-1.249461
H	-0.840724	2.825544	-1.475799
H	-2.871587	5.355878	0.454157
H	-2.237454	4.924025	2.062056
H	-1.210027	5.793823	0.902521
H	1.660635	-1.829863	3.512503
H	1.663974	-1.137554	1.866683
H	2.774365	-0.510992	3.099970
H	0.644182	-0.565843	5.447679
H	1.722045	0.818442	5.163996
H	-0.036965	1.063970	5.211862
H	0.111958	-3.863255	1.650411
H	-0.830495	-4.529829	0.299604
H	-0.165319	-2.873460	0.209285
H	-1.684907	-4.228700	3.360516
H	-3.346347	-3.650891	3.077979
H	-2.706322	-5.019082	2.141621

98

PC_CO (III)

C	2.336780	-1.960107	-1.861092
C	2.791209	-0.634452	-1.675755
C	3.078873	0.135388	-2.814616
C	2.899332	-0.359851	-4.106957
C	2.459520	-1.672340	-4.279846
C	2.193638	-2.462808	-3.160791
B	2.975041	0.027954	-0.239876
O	1.946695	0.284269	0.576894
Au	-0.028611	0.136129	0.255517
C	-1.982773	0.107730	0.052494
C	2.002268	-2.835221	-0.679841
C	4.444010	0.391359	0.244885
C	5.512162	-0.380913	-0.247897
C	6.827886	-0.197201	0.178251
C	7.105301	0.806084	1.106363
C	6.067462	1.604485	1.590677
C	4.739816	1.413798	1.183798
C	3.674884	2.318182	1.749707
C	2.477820	-0.599945	3.436864
O	2.968676	-1.568560	3.101405
H	6.292465	2.401158	2.303952
H	8.127916	0.975997	1.447821
H	7.629674	-0.823265	-0.216373
H	5.295696	-1.153987	-0.988842
H	3.118495	0.267294	-4.972794
H	2.333553	-2.085050	-5.282194
H	1.866823	-3.496421	-3.298174
H	3.455209	1.153531	-2.683946
H	3.001356	2.687399	0.965584
H	3.033230	1.779436	2.460533
H	4.130665	3.173704	2.266221
H	1.926659	-3.890386	-0.973894
H	1.038904	-2.540220	-0.233073
H	2.754450	-2.746849	0.116253
N	-2.854537	-0.928069	0.220787
C	-4.165097	-0.519443	0.011827
C	-4.115936	0.804029	-0.299980
N	-2.777474	1.168251	-0.269824
C	-2.265561	2.490629	-0.519457
H	-4.898114	1.513323	-0.537080
H	-4.999188	-1.202890	0.102493
C	-2.441483	-2.264261	0.558642
C	-2.169193	-2.561937	1.903286
C	-1.718554	-3.855301	2.192321
C	-1.556869	-4.799341	1.182968
C	-1.850902	-4.475689	-0.139837
C	-2.302402	-3.197630	-0.483802
C	-2.342099	-1.544027	3.015689
H	-1.488407	-4.123119	3.223956
H	-1.198520	-5.799716	1.428573
H	-1.718383	-5.226162	-0.918674
C	-2.559437	-2.816671	-1.931135

C	-1.827409	2.804332	-1.816814
C	-1.317415	4.091080	-2.023854
C	-1.247543	5.011283	-0.981962
C	-1.678942	4.663468	0.295846
C	-2.195040	3.390075	0.558385
C	-1.843661	1.794151	-2.949751
H	-0.958994	4.370644	-3.014941
H	-0.843209	6.007566	-1.164794
H	-1.600849	5.387993	1.106717
C	-2.581835	2.987723	1.970721
H	-3.122195	2.032164	1.916198
C	-1.319050	2.754261	2.816829
C	-3.519056	4.003141	2.635391
C	-0.411345	1.389245	-3.329519
H	-2.356087	0.889972	-2.592737
C	-2.621129	2.313018	-4.166758
C	-0.996917	-1.212727	3.675450
H	-2.722647	-0.614699	2.570010
C	-3.375692	-2.018280	4.047498
H	-3.206845	-1.928315	-1.936823
C	-1.238777	-2.430451	-2.613959
C	-3.289833	-3.912027	-2.715220
H	-3.823010	3.640429	3.626962
H	-3.026012	4.974865	2.775897
H	-4.424440	4.165500	2.034927
H	-1.591202	2.405512	3.823060
H	-0.666878	2.001582	2.351731
H	-0.743252	3.684992	2.919559
H	-0.421867	0.619633	-4.113102
H	0.154834	2.252704	-3.706602
H	0.130037	0.982843	-2.464241
H	-2.658907	1.540167	-4.946827
H	-3.651530	2.584153	-3.899014
H	-2.139075	3.200189	-4.600701
H	-3.532031	-3.549314	-3.723340
H	-2.669316	-4.811234	-2.832026
H	-4.225480	-4.206016	-2.220484
H	-1.418422	-2.095162	-3.644917
H	-0.723520	-1.625292	-2.073956
H	-0.558318	-3.291500	-2.648914
H	-3.524534	-1.247495	4.816470
H	-4.345311	-2.226004	3.574660
H	-3.042610	-2.935757	4.552693
H	-1.129422	-0.448594	4.453786
H	-0.552746	-2.102531	4.143417
H	-0.287751	-0.824897	2.931321

98

PC (III) – higher energy conformer

C	-4.517128	0.093367	-0.122281
N	-3.349495	-0.653816	-0.197720
C	-2.252892	0.153037	-0.131905
N	-2.755661	1.417492	-0.023235
C	-4.142405	1.399285	-0.028731
Au	-0.270695	-0.305080	-0.133539
C	1.734480	-0.507658	-0.079896
O	2.565765	-0.353340	-1.077147
B	3.717416	-0.362922	0.023326
C	4.643867	-1.652759	-0.069675
C	5.387275	-2.106019	1.045912
C	6.222033	-3.221879	0.911714
C	6.342399	-3.896445	-0.305183
C	5.612291	-3.458102	-1.409007
C	4.771457	-2.349559	-1.280535
C	5.290357	-1.398228	2.372722
C	-3.279621	-2.096111	-0.294220
C	-1.929211	2.605736	0.048073
C	4.446451	1.062978	0.128241
C	5.790010	1.107511	-0.288569
C	6.536016	2.286260	-0.318003
C	5.938780	3.477879	0.085852
C	4.609214	3.463918	0.506635
C	3.848264	2.285768	0.535133
C	2.419323	2.394450	1.005390
O	2.476026	-0.588819	0.993449
H	6.791468	-3.566025	1.778552
H	7.003929	-4.760303	-0.387284
H	5.695740	-3.975903	-2.365978
H	4.198948	-2.011672	-2.146510
H	7.576049	2.270129	-0.647949
H	6.500718	4.413338	0.077972
H	4.139703	4.397279	0.826726
H	6.267982	0.176350	-0.598109
H	5.583579	-0.342220	2.277697
H	4.256790	-1.403852	2.747476
H	5.935620	-1.870773	3.124187
H	2.197312	3.420515	1.323410
H	2.211843	1.715171	1.840883
H	1.703951	2.138347	0.209647
H	-4.731013	2.305009	0.028463
H	-5.496253	-0.365793	-0.136273
C	-1.337307	3.065645	-1.146226
C	-0.529238	4.205515	-1.083209
C	-0.316177	4.867494	0.118409
C	-0.909681	4.391120	1.281113
C	-1.727058	3.252560	1.284740
C	-1.555337	2.395551	-2.488113
H	-0.056838	4.571867	-1.994606
H	0.323841	5.749533	0.153205
H	-0.729812	4.901474	2.228679
C	-2.324129	2.866049	2.632554
C	-2.958663	-2.815319	0.874583
C	-2.857576	-4.207397	0.781975
C	-3.073569	-4.862691	-0.423352
C	-3.401934	-4.128619	-1.557594
C	-3.513565	-2.731845	-1.531170
C	-2.744588	-2.152639	2.221118
H	-2.601763	-4.781100	1.672987
H	-2.982211	-5.947653	-0.482921
H	-3.567696	-4.642912	-2.505496
C	-3.909665	-2.068914	-2.845558
C	-2.359459	1.373391	2.967610
H	-1.649614	3.333358	3.366570
C	-3.704679	3.512236	2.839018
C	-0.230467	1.977836	-3.139907
H	-2.130958	1.480736	-2.321262
C	-2.381938	3.296411	-3.418172
C	-1.328697	-2.400534	2.757620
H	-2.853860	-1.072325	2.088971
C	-3.813372	-2.599755	3.229270
C	-3.178676	-0.775371	-3.213630
H	-3.633463	-2.809509	-3.611759
C	-5.434684	-1.890013	-2.946426
H	-2.578759	2.781654	-4.368993
H	-3.346608	3.560765	-2.963210
H	-1.847559	4.230460	-3.642790
H	-0.426844	1.442334	-4.079067
H	0.397807	2.848000	-3.375346
H	0.341065	1.312896	-2.477510
H	-4.046659	3.348795	3.870684
H	-3.669666	4.593751	2.654178
H	-4.453970	3.073028	2.166735
H	-2.499721	1.245809	4.049589
H	-3.198403	0.868814	2.473406
H	-1.428542	0.867446	2.680223
H	-1.190428	-1.878661	3.714589
H	-1.144074	-3.470772	2.925758
H	-0.572176	-2.028253	2.051591
H	-3.681197	-2.066060	4.180734
H	-4.825713	-2.393188	2.855620

H	-3.742298	-3.677037	3.434984
H	-3.313025	-0.574304	-4.285127
H	-3.583761	0.087793	-2.671788
H	-2.102154	-0.842769	-3.010745
H	-5.707742	-1.564301	-3.959981
H	-5.960572	-2.829422	-2.731772
H	-5.793957	-1.126473	-2.243755

71

ICyAuB(*o*-tol)$_2$

C	-4.090287	-1.012516	0.963645
C	-3.051379	-1.053562	0.007320
C	-2.848728	-2.274455	-0.691901
C	-3.640830	-3.386377	-0.384957
C	-4.628114	-3.331023	0.601390
C	-4.856407	-2.133666	1.280304
B	-2.139317	0.220198	-0.086690
C	-2.867770	1.603423	-0.218792
C	-3.882023	1.688327	-1.199023
C	-4.491344	2.895060	-1.539689
C	-4.126607	4.059173	-0.862106
C	-3.159761	3.996781	0.143447
C	-2.522288	2.795504	0.474218
C	-1.516934	2.778441	1.591594
C	-1.827986	-2.374799	-1.790846
Au	-0.065368	0.053191	-0.026358
C	2.041149	-0.143812	0.033635
N	2.976558	0.845719	0.030268
C	4.258042	0.324010	0.063658
C	4.124671	-1.033211	0.082769
N	2.766123	-1.296227	0.069022
C	2.623830	2.271846	0.026157
C	2.145042	-2.627760	0.043380
H	4.876685	-1.811606	0.105624
H	5.147053	0.941726	0.070954
H	-2.891258	4.906045	0.686744
H	-4.600217	5.011595	-1.105391
H	-5.256675	2.925234	-2.317020
H	-4.185223	0.774615	-1.715127
H	-5.636582	-2.070227	2.040763
H	-5.223641	-4.216957	0.827552
H	-3.479935	-4.318865	-0.931476
H	-4.285169	-0.070378	1.481029
H	-1.949247	2.352933	2.509991
H	-0.663491	2.129765	1.326000
H	-1.156308	3.788982	1.825746
H	-1.618579	-3.420017	-2.054135
H	-0.892157	-1.871603	-1.488969
H	-2.170293	-1.858959	-2.700853
C	3.263580	3.013334	-1.148449
H	1.529680	2.271757	-0.107932
C	2.822380	4.480886	-1.151523
H	4.361764	2.961328	-1.062514
H	2.985903	2.518061	-2.089988
C	3.139065	5.164046	0.181904
H	3.307617	5.011943	-1.982754
H	1.736255	4.529426	-1.336809
C	2.517125	4.402459	1.355867
H	4.233395	5.208461	0.316293
H	2.779117	6.203054	0.170446
C	2.952560	2.933751	1.366414
H	2.785404	4.876438	2.310747
H	1.417857	4.449033	1.278133
H	4.038484	2.868366	1.547651
H	2.452248	2.382340	2.175696
C	2.629777	-3.504160	1.198478
H	1.070837	-2.421511	0.179508
C	1.922140	-4.862818	1.166352
H	3.718275	-3.658566	1.112832
H	2.446989	-2.988948	2.152424
C	2.110811	-5.560626	-0.183371
H	2.296764	-5.494774	1.984008
H	0.845332	-4.712271	1.351376
C	1.643917	-4.669808	-1.337553
H	3.178387	-5.803480	-0.320375
H	1.565020	-6.515151	-0.196008
C	2.343245	-3.307354	-1.313708
H	1.823249	-5.161981	-2.304014
H	0.555001	-4.514178	-1.259534
H	3.422452	-3.439008	-1.499055
H	1.952795	-2.652604	-2.106223

74

ICyAuCO$_2$B(*o*-tol)$_2$

C	3.900982	2.628751	1.111629
C	3.899119	1.765007	0.006196
C	4.572398	2.177471	-1.168132
C	5.214788	3.421130	-1.193919
C	5.210991	4.264038	-0.080606
C	4.550144	3.865509	1.080442
B	3.184731	0.345758	0.094037
O	2.017753	0.327415	1.181012
C	1.192648	0.254436	0.169010
O	1.942500	0.259043	-0.901003
C	4.604862	1.293428	-2.388236
Au	-0.809796	0.001665	0.228214
C	-2.814766	-0.389863	0.229800
N	-3.856879	0.480407	0.176782
C	-5.059820	-0.198514	0.098754
C	-4.760546	-1.528555	0.106448
N	-3.383708	-1.625547	0.195516
C	-3.697135	1.942077	0.183002
C	-2.589493	-2.865994	0.161510
C	4.124551	-0.950001	0.177093
C	5.463554	-0.729777	0.550995
C	6.385071	-1.764952	0.709355
C	5.977415	-3.077890	0.485529
C	4.655837	-3.327195	0.115579
C	3.720321	-2.294350	-0.039145
C	2.314303	-2.678281	-0.431320
H	5.731003	3.732640	-2.105213
H	5.722895	5.226834	-0.122914
H	4.538947	4.513768	1.958263
H	3.381506	2.322698	2.021676
H	7.413561	-1.545001	0.999528
H	6.679757	-3.905611	0.596467
H	4.334342	-4.356645	-0.059143
H	5.793220	0.297084	0.718288
H	5.054681	0.316997	-2.155466
H	3.588998	1.091363	-2.756658
H	5.182300	1.755975	-3.198802
H	2.238736	-3.765659	-0.562090
H	2.004405	-2.192385	-1.364822
H	1.584866	-2.379591	0.336060
H	-5.409398	-2.393088	0.065271
H	-6.016082	0.305826	0.053434
C	-4.445460	2.584118	1.352663
H	-2.614839	2.094277	0.325039
C	-4.219865	4.099245	1.359393
H	-5.523700	2.375367	1.254468
H	-4.109690	2.131850	2.296867
C	-4.617769	4.730591	0.022271
H	-4.786068	4.552272	2.185315
H	-3.154650	4.304526	1.557177
C	-3.880308	4.067274	-1.144315
H	-5.705522	4.617300	-0.124013
H	-4.410767	5.810126	0.036764
C	-4.105170	2.552464	-1.160099
H	-4.202568	4.498881	-2.102360
H	-2.799904	4.269958	-1.055148

H	-5.170270	2.337278	-1.347110
H	-3.528749	2.078020	-1.967253
C	-2.338385	-3.318139	-1.280013
H	-1.622548	-2.582002	0.607598
C	-1.444674	-4.561594	-1.297925
H	-3.305713	-3.540564	-1.760389
H	-1.873205	-2.494201	-1.840443
C	-2.033465	-5.687118	-0.443184
H	-1.298549	-4.897717	-2.334096
H	-0.447633	-4.291343	-0.911493
C	-2.306460	-5.213779	0.987513
H	-2.977705	-6.032252	-0.897560
H	-1.353184	-6.550752	-0.432023
C	-3.210900	-3.976014	1.006462
H	-2.769336	-6.016394	1.578599
H	-1.350855	-4.969052	1.480440
H	-4.197540	-4.247629	0.596838
H	-3.367408	-3.619974	2.034827

41

[^{t}Bu$_3$PAu]·

Au	-1.537303191	-0.127728329	-0.018814744
P	0.834911329	0.004149610	-0.000118891
C	1.514070752	-1.126828234	1.394432393
C	0.700708247	-2.436671123	1.392092234
C	1.508764649	-0.583875578	-1.698649670
C	0.604919926	0.001117428	-2.802041581
C	1.369432224	1.819363904	0.320027000
C	0.444650843	2.406897203	1.405325215
C	2.972154375	-0.215227845	-1.977918232
C	1.346668461	-2.110673535	-1.785426427
C	2.835706322	1.999405717	0.734919582
C	1.100144092	2.639686406	-0.952438454
C	3.011826039	-1.446413012	1.296124315
C	1.227520215	-0.457111383	2.748593312
H	1.565579717	-2.415106229	-2.819925411
H	2.038151775	-2.651167546	-1.131105641
H	0.318390725	-2.419684644	-1.555547148
H	0.892971133	-0.459670592	-3.759300641
H	-0.451062463	-0.234329488	-2.608423192
H	0.699347290	1.084110055	-2.911526274
H	3.261778213	-0.649347958	-2.948059876
H	3.122585650	0.867762906	-2.049139104
H	3.657182281	-0.610160022	-1.219503545
H	0.989291893	-3.017757778	2.281307951
H	-0.376950515	-2.228468420	1.448972420
H	0.882478612	-3.059508997	0.513164704
H	1.452284303	-1.187355975	3.540525107
H	1.849753395	0.425063484	2.929752163
H	0.170004462	-0.176844053	2.842417204
H	3.297715858	-2.050771729	2.171683433
H	3.255243782	-2.033704539	0.403712965
H	3.634824960	-0.545050261	1.297354734
H	0.646621793	3.486456202	1.478084383
H	-0.611898692	2.269619276	1.135615879
H	0.604391030	1.970548675	2.394073585
H	3.038408644	3.077389555	0.837071909
H	3.055650997	1.534802226	1.702355353
H	3.534389501	1.598953101	-0.008143351
H	1.236202082	3.702925071	-0.703938805
H	1.790969740	2.402321811	-1.767876051
H	0.068757219	2.507776082	-1.305349846

66

[IPrAu]·

C	-3.056236240	1.177172272	0.822247193
C	-2.374390869	-0.046387840	0.937193763
C	-2.964758970	-1.287601184	0.649694441
C	-4.302980158	-1.279768841	0.239796430
C	-5.009022188	-0.086036340	0.122018741
C	-4.391431731	1.129236089	0.408125078
N	-0.997771936	-0.023024983	1.356978573
C	-0.573571317	-0.030026537	2.680330437
C	0.786376760	-0.003745523	2.649427222
N	1.149318916	0.016147283	1.308247662
C	0.056590077	0.004055717	0.495541405
Au	0.007932141	0.015834171	-1.577924658
C	2.504983019	0.017395037	0.825184899
C	3.117368087	1.250299637	0.548627800
C	4.431475949	1.217773160	0.068384426
C	5.093829099	0.008651385	-0.124190722
C	4.455950790	-1.197240888	0.156230371
C	3.142988876	-1.220543603	0.638329952
C	2.384847223	2.570223510	0.703847717
C	2.052282268	3.158845783	-0.675756197
C	2.425866095	-2.537268334	0.875912052
C	3.258981383	-3.518173383	1.709308397
C	-2.193765346	-2.592522355	0.728875752
C	-2.866828774	-3.597501002	1.673253380
C	-2.361152772	2.505866280	1.057379132
C	-3.193895837	3.466671558	1.913897410
C	3.168296164	3.568170069	1.566067969
C	2.003791220	-3.155718294	-0.466621392
C	-1.994303091	-3.182735826	-0.675039995
C	-1.980016163	3.141045463	-0.290115329
H	1.520688979	0.001875169	3.444975820
H	-1.270609795	-0.051728544	3.508412910
H	4.939076653	2.153563301	-0.166858853
H	6.116152750	0.005217762	-0.504184300
H	4.982325438	-2.136716771	-0.012715098
H	-4.795119683	-2.223033771	0.001047549
H	-6.049955155	-0.101523766	-0.203259472
H	-4.952680523	2.056924494	0.297032715
H	1.507824767	-2.325725975	1.441934735
H	1.431701042	2.370565334	1.212807685
H	-1.196604749	-2.373749727	1.135097844
H	-1.427522734	2.307441292	1.602293491
H	2.675465489	-4.425935364	1.915190893
H	4.171315284	-3.825727388	1.179791997
H	3.556977403	-3.074706959	2.669200428
H	1.433590096	-4.080526297	-0.300105407
H	1.376908623	-2.455858175	-1.039032733
H	2.886289991	-3.400230157	-1.075036584
H	2.583080253	4.487865702	1.703329527
H	3.393093606	3.150886989	2.557146054
H	4.119572176	3.848126468	1.092527593
H	1.476045477	4.088273884	-0.566571794
H	2.971336831	3.387708073	-1.234057802
H	1.458069703	2.446967732	-1.268540166
H	-1.394800067	-4.102228923	-0.621490212
H	-2.959816192	-3.430151238	-1.138753919
H	-1.473041470	-2.464305205	-1.325342617
H	-2.260697682	-4.511051825	1.746522362
H	-2.985311578	-3.180141198	2.682616156
H	-3.862202195	-3.885737448	1.307204699
H	-1.421244156	4.073903376	-0.130054438
H	-1.356426653	2.456034564	-0.883652327
H	-2.881187809	3.375111200	-0.874933874
H	-2.619529147	4.380616345	2.117938188
H	-4.118892274	3.767626429	1.402888578
H	-3.469470317	3.010836241	2.874683475

29

[B(*o*-tol)$_2$]·

B	-0.021498151	0.230184836	-0.187905022
C	1.429383972	-0.023942200	-0.198376934
C	2.068427373	-0.795798654	0.828459942
C	3.449202021	-0.957286697	0.793714161
C	4.233383425	-0.374206022	-0.209436975
C	3.619486641	0.379400390	-1.218462356
C	2.242488330	0.537850911	-1.230450301

C	1.235126424	-1.430955917	1.902787075
C	-1.440009080	0.622824164	-0.125941477
C	-1.790936091	1.998274444	-0.294609132
C	-3.118568736	2.394300925	-0.338281892
C	-4.141242698	1.450713489	-0.175165172
C	-3.815390536	0.102921145	0.020392255
C	-2.495242182	-0.334061004	0.040341653
C	-2.154595500	-1.779181817	0.256448124
H	3.928922807	-1.544961775	1.579391961
H	5.314792410	-0.510612833	-0.205220282
H	4.221586070	0.832194287	-2.007055802
H	1.772168645	1.108741363	-2.032230672
H	-3.362821213	3.447224440	-0.484006176
H	-5.185624487	1.762146186	-0.196139336
H	-4.613942124	-0.631077904	0.148054205
H	-1.001780013	2.743901050	-0.400592535
H	0.434934499	-0.746055921	2.230243453
H	0.743233616	-2.345713218	1.537241374
H	1.839867044	-1.704308894	2.776383080
H	-3.002934338	-2.435226421	0.024939307
H	-1.860852055	-1.968839227	1.300343484
H	-1.299001428	-2.073453867	-0.375146462

53

[Al(NON')]·

Al	0.034273963	-0.866771034	-1.068112143
N	1.779460574	-0.564760649	-0.368273418
C	2.656494307	-1.626654461	-0.050335398
N	-1.655575575	-0.802592874	-0.181396488
C	-2.124662417	0.469292735	0.138228785
C	-1.270596628	1.525721988	-0.210492483
C	-1.389381067	2.857457527	0.138866377
C	-2.550637918	3.195362197	0.849881995
C	-3.474372369	2.196168098	1.176456588
C	-3.280606292	0.852050295	0.846590028
C	-0.242924336	3.802550850	-0.254108782
C	1.067918394	3.033291682	-0.032302597
C	1.077306343	1.688648545	-0.343826611
O	-0.095434342	1.099054160	-0.873150411
C	2.099371512	0.761520341	-0.096767484
C	3.302557860	1.320694510	0.376610885
C	3.365706431	2.690435098	0.647507641
C	2.268889578	3.544415947	0.483929925
C	-0.277016398	5.099197488	0.556515659
C	-0.371749314	4.143775284	-1.760481936
C	-2.447661353	-1.960263215	-0.081955429
H	4.168705067	0.686090296	0.553981090
H	2.351319030	4.593695603	0.760208901
H	-2.728711012	4.222296531	1.162881637
H	-3.996935500	0.101790104	1.174495899
H	-1.217375609	5.635226087	0.377899345
H	0.539618067	5.762772633	0.246345606
H	-0.182249581	4.906098983	1.632777346
H	-1.319728707	4.666645015	-1.945662929
H	-0.349095289	3.238558048	-2.379691313
H	0.457988779	4.794303565	-2.067689099
H	4.302952529	3.102286557	1.023594338
H	-4.371329814	2.469385261	1.733630182
C	-1.816658462	-3.163555485	0.284504270
C	-2.521945353	-4.365920233	0.301951511
C	-3.878970147	-4.390704000	-0.023696151
C	-4.515653723	-3.199165470	-0.382200390
C	-3.812741593	-1.996643031	-0.419440358
H	-0.763486373	-3.142923815	0.577349592
H	-2.008962599	-5.285247623	0.586831261
H	-4.434954449	-5.327946029	-0.000870391
H	-5.572362711	-3.206814578	-0.653033667
H	-4.312987032	-1.085299588	-0.744087911
C	3.640360099	-3.763141304	-0.671982266
C	4.270943294	-3.850978624	0.569954083
C	4.088288729	-2.826266207	1.502989052
C	3.283546960	-1.728904147	1.204142867
H	2.375944332	-2.577220103	-1.965471928
H	3.775117066	-4.553024257	-1.412043166
H	4.898718426	-4.709094362	0.810247722
H	4.565770241	-2.888334635	2.481770550
H	3.119178669	-0.952458820	1.950761572
C	2.848093010	-2.658511385	-0.984903603

71

[(tBu3P)AuOB(o-tol)2]

C	-5.018036333	-0.622448393	0.142730181
C	-4.121626717	0.461849846	0.067164296
C	-4.669173933	1.759513253	-0.125904418
C	-6.058652571	1.906665567	-0.235359720
C	-6.922039394	0.812844037	-0.159952857
C	-6.399108464	-0.465332607	0.032037137
B	-2.570458841	0.147361909	0.171186658
O	-1.661305483	1.133798084	0.091137851
AU	0.325853721	0.662630653	0.043745355
P	2.513373496	0.138771541	-0.018675826
C	2.922376376	-0.640595180	-1.722401605
C	1.766684965	-1.578512115	-2.121845170
C	-3.809097755	2.992065590	-0.219026588
C	-2.096105766	-1.367003852	0.369494243
C	-1.852688430	-1.865272708	1.660000844
C	-1.394357065	-3.167116815	1.872762072
C	-1.166420375	-4.002089059	0.779130009
C	-1.400179882	-3.525340636	-0.512526180
C	-1.867766759	-2.223935820	-0.731343030
C	-2.131356011	-1.735313617	-2.132128958
C	3.574638978	1.712469947	0.248525092
C	2.957647186	2.866100262	-0.566999088
C	2.856252125	-1.130695079	1.375938958
C	2.081550581	-0.695374903	2.636501381
C	3.466805003	2.133836506	1.723594486
C	5.054863544	1.553748916	-0.124332211
C	2.257226045	-2.486205057	0.965894085
C	4.340658109	-1.314131862	1.718459708
C	2.947171878	0.471340581	-2.784849090
C	4.250707769	-1.407999022	-1.766677262
H	-6.472497341	2.906805056	-0.384379798
H	-7.999304702	0.962360581	-0.250451876
H	-7.061094245	-1.330330215	0.094466535
H	-4.608140624	-1.623425118	0.293102407
H	-1.213813223	-3.526563285	2.887135887
H	-0.805081866	-5.020728936	0.928152418
H	-1.219852578	-4.177886872	-1.370287585
H	-2.026288577	-1.217098418	2.523075213
H	-3.201145262	3.122555520	0.685996423
H	-3.089676023	2.912683036	-1.044817141
H	-4.430009815	3.885775366	-0.366216857
H	-1.825508011	-2.475137747	-2.882750556
H	-1.593296479	-0.796647004	-2.332613031
H	-3.200636208	-1.518868597	-2.277797003
H	1.963193939	-1.943106371	-3.141566987
H	1.657518558	-2.446933454	-1.469228550
H	0.809935903	-1.040698863	-2.127523810

H	3.003227747	-0.008453355	-3.773261067
H	2.029961752	1.074466071	-2.758389031
H	3.813982587	1.133568310	-2.692744339
H	4.415532984	-1.753372157	-2.798708278
H	5.108002095	-0.786737196	-1.485202606
H	4.241372687	-2.295971842	-1.125306388
H	2.299565985	-3.150103582	1.842169834
H	1.202702061	-2.396744115	0.676897587
H	2.814079234	-2.973654932	0.159249999
H	2.219793300	-1.474754623	3.401192950
H	2.425442684	0.251888857	3.058177400
H	1.007051064	-0.613267571	2.425037757
H	4.423399317	-2.108707146	2.475634067
H	4.939100631	-1.619514301	0.852930340
H	4.787967000	-0.410482308	2.147129014
H	5.577960947	2.488237801	0.129901246
H	5.544210760	0.741179437	0.423742304
H	5.198896039	1.383747573	-1.196996873
H	3.496390003	3.790221964	-0.308260528
H	3.036303186	2.726351630	-1.647599917
H	1.897827459	3.003035577	-0.313887979
H	3.937682018	3.122631187	1.827261922
H	2.419373733	2.227615215	2.039565441
H	3.985548827	1.450464995	2.403315789

96

[(IPr)AuOB(o-tol)2]

C	1.912897144	-3.432207199	-0.175015594
C	1.955951266	-2.370004606	-1.094273540
C	1.409859338	-2.444496030	-2.386069464
C	0.810630958	-3.653395352	-2.756976992
C	0.759136306	-4.728564238	-1.874262534
C	1.302911450	-4.617986281	-0.596970086
N	2.563053192	-1.132663528	-0.679192432
C	1.870113771	-0.114584169	-0.092970647
N	2.815707270	0.839079333	0.151874606
C	4.071319090	0.419984905	-0.267023181
C	3.911005719	-0.823877628	-0.794166020
AU	-0.038680441	-0.086736836	0.374789437
O	-1.947320269	-0.159570474	1.000078879
B	-3.069670264	0.108169479	0.319915171
C	-4.476706551	-0.241903402	0.969764756
C	-5.619780715	0.408875292	0.467114976
C	-6.895443250	0.197106504	0.989886802
C	-7.056538557	-0.709396400	2.037078160
C	-5.942943950	-1.379586315	2.545932847
C	-4.654079008	-1.157968840	2.041355377
C	-3.504210785	-1.913498027	2.656632046
C	2.524543987	2.103998989	0.773354111
C	2.395889010	2.149339488	2.171272476
C	2.078299502	3.386939994	2.743158591
C	1.899108581	4.516857968	1.951749267
C	2.040113011	4.438857990	0.567718230
C	2.358255104	3.228187065	-0.055554842
C	2.588204279	0.927787677	3.051940905
C	3.797229687	1.103055193	3.982841474
C	2.449329688	3.123568167	-1.568350340
C	3.146991106	4.328548013	-2.209037452
C	1.404121989	-1.259263529	-3.334020654
C	2.028618481	-1.606344265	-4.691813602
C	2.437824070	-3.295659970	1.242987278
C	3.385929365	-4.438506644	1.625817460
C	-3.036077046	0.756868119	-1.135306102
C	-2.553775254	2.063897761	-1.377615420
C	-2.505895113	2.548455567	-2.690761903
C	-2.914593841	1.763087671	-3.770109263
C	-3.398671700	0.475472381	-3.540813103
C	-3.465888033	-0.006626495	-2.232778940
C	-2.102064700	2.940647993	-0.237239707
C	1.313040122	0.602425803	3.842108907
C	1.051185772	2.910135462	-2.168962420
C	-0.022348929	-0.713747791	-3.493048130
C	1.267393168	-3.182129428	2.233453238
H	-6.074800877	-2.095573600	3.360695617
H	-8.045820734	-0.900776360	2.456367250
H	-7.756350958	0.727323686	0.579536818
H	-5.494667916	1.105246549	-0.364683937
H	-3.730203111	-0.145679919	-4.374618136
H	-2.863965993	2.161229107	-4.784884291
H	-2.144747479	3.563921278	-2.870343609
H	-3.864878614	-1.008946293	-2.056154537
H	-2.893188413	-2.410839360	1.891750017
H	-2.819328587	-1.233025121	3.180200064
H	-3.873568850	-2.664527013	3.367781405
H	-1.985910051	3.984046970	-0.558260252
H	-1.134826367	2.599114956	0.164737342
H	-2.815810435	2.910830445	0.597997848
H	4.620047056	-1.511574843	-1.236184998
H	4.949622591	1.041739030	-0.152603457
H	1.966049294	3.462628888	3.825139650
H	1.646140294	5.470085544	2.417290527
H	1.892831761	5.332296835	-0.038205596
H	0.367244655	-3.748361704	-3.748380435
H	0.282754766	-5.660368444	-2.181337985
H	1.241578677	-5.462756937	0.089219608
H	3.012178400	-2.360522603	1.304026170
H	2.013499013	-0.461709429	-2.886348329
H	2.794567541	0.067341437	2.401088899
H	3.050026222	2.235103095	-1.810277626
H	3.793448223	-4.268500467	2.631807979
H	2.866357481	-5.406573317	1.638619637
H	4.225814805	-4.514265629	0.921943952
H	1.643625307	-3.028492662	3.254709094
H	0.613003857	-2.338303882	1.972166714
H	0.660318452	-4.098532057	2.225615396
H	-0.027608761	0.176961430	-4.135594594
H	-0.682580809	-1.465261014	-3.947985714
H	-0.453970974	-0.433649643	-2.522636989
H	2.056985101	-0.713835072	-5.331995297
H	3.054618273	-1.982110842	-4.577462058
H	1.443609331	-2.373468040	-5.217956505
H	3.283714090	4.150454821	-3.284271273
H	2.551984299	5.246310320	-2.103888998
H	4.133592597	4.508874859	-1.761176551

H	1.115925115	2.775563627	-3.257703293
H	0.556338895	2.027917816	-1.742258623
H	0.410122166	3.779530979	-1.968199543
H	3.951245654	0.193750974	4.580248513
H	4.715014114	1.300763961	3.412344788
H	3.644445918	1.940972904	4.677595309
H	1.457331375	-0.311825154	4.434235266
H	1.052865659	1.416309325	4.533140001
H	0.464843224	0.442647419	3.161937987
